# Comparative Study of Iminodibenzyl and Diphenylamine Derivatives as Hole Transport Materials in Inverted Perovskite Solar Cells

**DOI:** 10.1002/chem.202404251

**Published:** 2025-01-28

**Authors:** Mauricio Caicedo‐Reina, Juan S. Rocha‐Ortiz, Jianchang Wu, Andreas J. Bornschlegl, Salvador Leon, Anastasia Barabash, Jose Dario Perea, Yunuo Wang, Vanessa Arango‐Marín, Alejandro Ortiz, Larry Lüer, Jens A. Hauch, Braulio Insuasty, Christoph J. Brabec

**Affiliations:** ^1^ Department of Chemistry Grupo de Investigación de Compuestos Heterocíclicos Universidad del Valle Calle 13 #100-00 760032 Cali Colombia; ^2^ Department of High Throughput Methods in Photovoltaics Forschungszentrum Jülich GmbH Helmholtz-Institute Erlangen-Nürnberg (HI ERN) Immerwahrstraße 2 91058 Erlangen Germany; ^3^ Department of Materials Science and Engineering Institute of Materials for Electronics and Energy Technology (i-MEET) Friedrich-Alexander-Universität Erlangen-Nürnberg Martensstraße 7 91058 Erlangen Germany; ^4^ Department of Chemical and Environmental Engineering ETSIIM Universidad Politécnica de Madrid José Gutiérrez Abascal 2 28006 Madrid Spain; ^5^ Departament of biochemical Engineering Universidad Icesi Calle 18 # 22-135 760031 Cali Colombia

**Keywords:** Iminodibenzyl, Fluorene-based derivatives, Photostability, Hole transport materials, Perovskite solar cells

## Abstract

Perovskite solar cells (PSCs) have recently achieved over 26 % power conversion efficiency, challenging the dominance of silicon‐based alternatives. This progress is significantly driven by innovations in hole transport materials (HTMs), which notably influence the efficiency and stability of PSCs. However, conventional organic HTMs like Spiro‐OMeTAD and PTAA, although highly efficient, suffer from thermal degradation, moisture ingress, and high cost. This study explores the potential of iminodibenzyl, a moiety known for its strong electron‐donating capabilities in pharmaceutical applications, as a novel HTM. A series of fluorene‐based derivatives incorporating iminodibenzyl (**TMF‐2** and **TDF‐2**) and diphenylamine (**TMF‐1** and **TDF‐1**) units were synthesized and characterized. The new HTMs demonstrated commendable optical, electrochemical, and thermal properties, as well as enhanced photostability. Among them, **TDF‐2** achieved a power conversion efficiency (PCE) of 19.38 %, the highest of the new materials. Although these efficiencies are slightly lower than the benchmark PTAA (20.20 %), the study underscores the potential of iminodibenzyl to enhance photostability and increase HOMO levels, making it a promising candidate for future HTM development in PSCs.

## Graphical abstract

This study introduces novel fluorene‐based hole transport materials (HTMs) for perovskite solar cells (PSCs), integrating iminodibenzyl and diphenylamine moieties. These materials exhibit remarkable photostability, optical, and thermal properties. Among them, TDF‐2 achieved a power conversion efficiency (PCE) of 19.38 %. The findings highlight iminodibenzyl's potential to address stability and cost challenges, paving the way for next‐generation HTMs in PSCs.

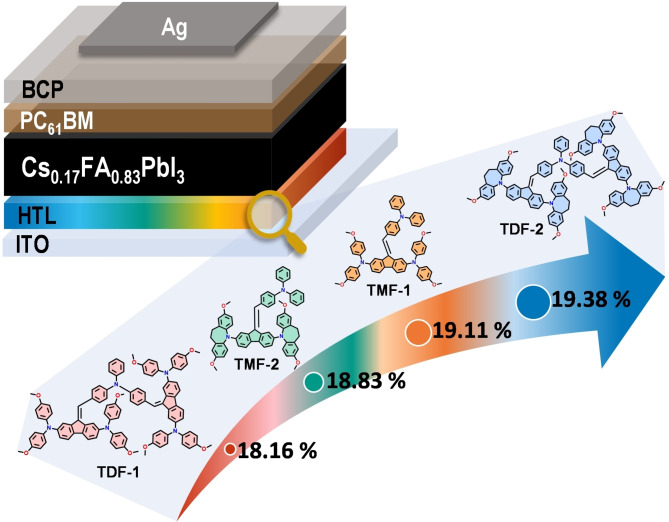

## Introduction

As the global quest for sustainable energy solutions intensifies, halide perovskite solar cells (PSCs) have emerged as a leading contender in the field. Demonstrating a remarkable trajectory, PSCs have advanced from less than 4 % power conversion efficiency in 2009 to over 20 % by 2015, showing that this type of materials are unique by featuring the fastest efficiency improvement realized for any solar cell technology.[^1^, ^2^, ^3^] The n‐i‐p structure typically outperforms p‐i‐n due to lower open‐circuit voltage (Voc) and fill factor (FF) in the latter, largely caused by carrier recombination at the interfaces between the perovskite and the transport layers. However, the p‐i‐n PSCs present benefits over n‐i‐p PSCs including lower‐temperature sintering (suitable for tandem cells and flexible devices), improved stability due to dopant‐free hole transport layers (HTLs), reduced hysteresis in current‐voltage characteristics, and potentially lower costs from using inexpensive back electrodes like Ag, Al, or Cu instead of Au. Some common HTLs are Spiro‐OMeTAD for n‐i‐p and PTAA for p‐i‐n PSCs.[^4^, ^5^] Recent advancements in the inverted configuration have led to significant efficiency improvements, with certified values now surpassing 26 %.^[6]^ This astounding progress not only challenges the dominance of silicon‐based alternatives but also underscores the pivotal role of innovative material development in driving the evolution of these devices.[^7^, ^8^, ^9^] Central to this evolution is the innovation in hole transport materials (HTMs), key components that greatly influence the efficiency and stability of PSCs.^[10]^ The organic HTMs offer advantages such as solution processing, tunable properties through molecular design, and extensive libraries of building blocks. Although PTAA in inverted architectures has achieved high efficiencies, it still presents several challenges that impact device performance. These challenges include thermal degradation, moisture ingress, mechanical failure, sub‐optimal conductivity, and significant recombination at the perovskite/HTL interface.^[11]^ Furthermore, their application beyond research settings is limited due to their prohibitively high cost.

However, polycyclic aromatic hydrocarbons (PAHs) have been extensively utilized as a building block for organic semiconductors due to its low‐cost, structurally versatile, and broadly tunable optoelectronic properties.[^12^, ^13^, ^14^, ^15^, ^16^] Additionally, the triarylamine (TAA) core, with its distinctive non‐planar, propeller‐like shape and sterically hindered *sp* ^2^ nitrogen, enhances electronic properties such as hyperconjugation. These features contribute to PTAA′s high hole mobility, low oxidation potential, and reversible redox behavior, making it essential in organic photovoltaics, including light‐emitting diodes and solar cells. ^[17–24]^ The importance of this core is underscored by molecules achieving similar or superior performance than PTAA, with one achieving a record 26.2% efficiency.^[25]^ That research has shown that the combination of TAA^[26]^ and fluorene leads to enhanced performance, surpassing that of the reference materials, while significantly reducing the harsh or costly conditions required for their synthesis. ^[27,28]^

In this work we combined the features of these two units and ventured beyond conventional boundaries, exploring the potential of iminodibencyl, a compound predominantly known in pharmaceutical applications.[^29^, ^30^] Although its application in HTM development has been limited thus far,^[28]^ previous studies have demonstrated that its strong electron‐donating capabilities can significantly enhance photovoltaic performance, particularly in sensitizers.[^32^, ^33^] This research conducts a comparative analysis examining the impact of incorporating iminodibencyl versus diphenylamine into the HTM structure. The aim of this synthesis is to pioneer a new class of materials for use in photovoltaic technology, potentially broadening the horizons of sustainable energy sources.

## Results and Discussion

### Synthesis

**Figure 1:**
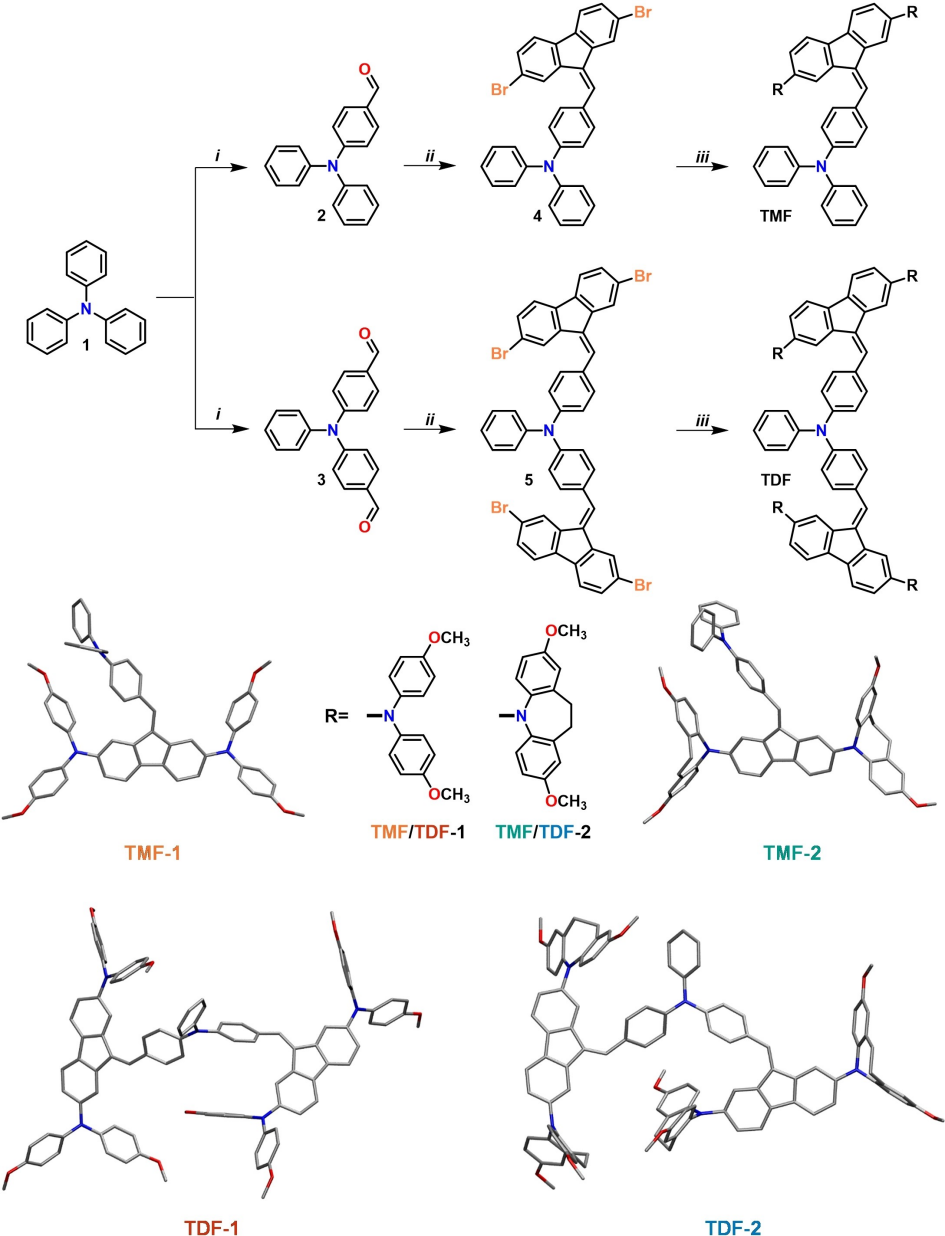
Figure caption. General procedure for the synthesis of **TMF** and **TDF** series and their DFT optimized geometries. Reagents and conditions: *i*) POCl_3_, DMF, 80 °C, 8–12 h, *ii*) NaOH (40 %), TBAB, toluene, 100 °C, 2 h, *iii*) **7** or **8**, Pd_2_(dba)_3_, XPhos, *t*‐BuOK, toluene, 90 °C, 18 h.

The detailed synthetic route for the preparation of series **TMF** and **TDF** is depicted in Figure 1 and Scheme S1. Initially, Aldehydes **2** and **3** were synthesized through a Vilsmeier‐Haack reaction using triphenylamine (**1**) as the starting material. Subsequently, these aldehydes underwent a condensation reaction with 2,7‐dibromofluorene in a basic aqueous‐toluene medium, with tetrabutylammonium bromide serving as a phase transfer catalyst, resulting in the formation of the vinylenes **4** and **5**. These compounds were then subjected to a Buchwald‐Hartwig amination reaction with 4,4′‐dimethoxydiphenylamine (**7**), which was facilitated by the use of Pd_2_(dba)_3_ as catalyst and XPhos as ligand, culminating in the synthesis of **TMF‐1** and **TDF‐1**. A parallel amination of vinylenes (**4**) and (**5**) with dimethoxyiminodibenzyl (**8**), under similar conditions, afforded **TMF‐2** and **TDF‐2**. All the detailed information about the synthetic procedures and characterization are available in the supporting information. A detailed cost analysis for the products of the TMF and TDF series is presented in Tables S1‐S8, based on the methods employed in previous literature reports.[^34^, ^35^]

The four target compounds from the **TMF** and **TDF** series were subjected to ab initio DFT quantum chemical calculations. Geometry optimizations were performed at the B3LYP/6‐31G(d) level, in THF solution. The resulting conformations, displayed in Figure 1, do not exhibit significant differences regarding the geometry of the common units of the different compounds.

### Optical, Electrochemical and Thermal Properties

**Figure 2:**
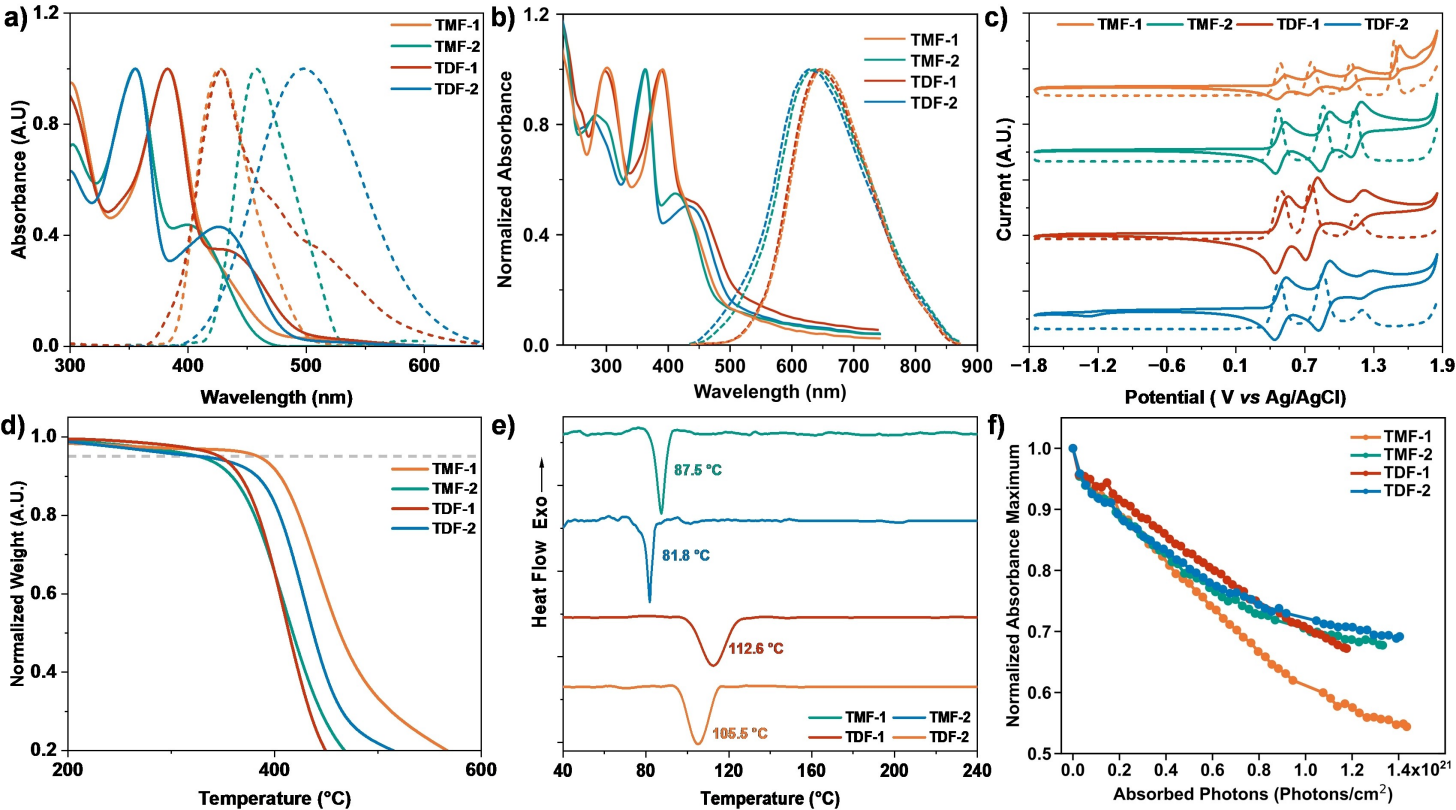
Normalized UV‐Vis (solid line) and fluorescence spectra (dashed line) in **a)** THF solutions at 1×10^−5^ M and in **b)** thin film on a quartz substrate, **c)** cyclic (solid line) and differential pulse (dashed line) voltammograms of the anodic scan at 100 mV s^−1^ in dry 0.1 M TBAPF_6_ CH_2_Cl_2_ solution at 21±1 °C (V *vs* Ag/AgCl), **d)** thermogravimetric analysis curves at 10 °C min^−1^ of heating rate and **e)** differential scanning calorimetry curves under nitrogen at a heating rate of 20 °C min^−1^ for the series **TMF** and **TDF**. **f)** Evolution of main absorbance peaks (see Figure S13) of the thin films on quartz substrates during UVC degradation over the number of absorbed UVC photons.

The effect of different chemical structures on their optical properties was studied by analyzing the absorption and fluorescence spectra of fluorene derivatives, presented in Figure 2a. These derivatives were dissolved in THF at a concentration of 1×10^−5^ M. Table 1 summarizes the photophysical parameters. The optical bandgap was determined from the wavelength at the intercept between the fluorescence and absorption spectra (λ_onset_), using the formula E_0–0_=1240/λ_onset_.

**Table 1:** Optical, electrochemical and thermal properties of **TMF** and **TDF** derivatives.

	λ max, abs. [nm]		λ max, em. (λ ex) [nm]		*E* ^ox^_1_^[b]^ [V]	*E*_HOMO_^[c]^ [eV]	*E*_0–0_^[d]^ [eV]	*E*_LUMO_^[e]^ [eV]	*T*_dec_^[f]^ [°C]
	THF	Film	THF	Film^[a]^					
TMF‐1	383	301, 390	427 (383)	650	0.44	−4.84	3.06	−1.78	384
TMF‐2	355	363	457 (399)	638	0.42	−4.82	2.84	−1.98	327
TDF‐1	383	296, 387	428 (382)	650	0.45	−4.85	3.06	−1.79	347
TDF‐2	356	362	498 (425)	628	0.43	−4.83	2.72	−2.11	329

[a] All films were exited with a laser at 402 nm. [b] Determined from differential pulse voltammetry peaks versus silver/silver chloride reference electrode (Ag/AgCl). [c] E_HOMO_ was calculated by E_HOMO_ (eV)*=*−(E^ox^_1_+4.4). [d] The optical bandgap was calculated from the onset of the lower energy absorption edge (*E*_0–0_ (eV)=1240/λ_onset_). [e] E_LUMO_ was calculated by E_LUMO_ (eV)=E_HOMO_+*E*_0–0_. [f] Decomposition temperature determined from TGA (5 % weight loss under a N_2_ atmosphere).

In the UV‐Vis spectra (Figure 2a), **TMF‐1** displays a prominent absorption band at 382 nm, accompanied by a notable, more energetic shoulder near 430 nm. In contrast, **TMF‐2** exhibits a sharp absorption at 355 nm, alongside a moderately intense band centered at 400 nm. This variation suggests a possible correlation: the shoulder observed in **TMF‐1** at 430 nm may correspond to the 400 nm transition seen in **TMF‐2**. This alignment hints at the involvement of the arylamine fragment. The underlying cause appears to be a state of greater rigidity in **TMF‐2**, which restricts the rotation of the arylamine phenyls within the iminodibenzyl structure. This restriction results in the splitting of these bands. Conversely, such splitting is absent in **TMF‐1**, where the band manifests instead as a shoulder.

Turning to the **TDF** series, their spectral characteristics show a notable resemblance to their monovinyl analogs. Specifically, **TDF‐1** is characterized by a narrow yet intense absorption band at 383 nm, along with a less intense band at 434 nm that slightly merges with the main band. In the case of **TDF‐2**, the spectrum reveals a smaller, somewhat broader band at 427 nm, accompanied by a more distinct band at 356 nm. This series also demonstrates the band splitting characteristic of the iminodibenzyl derivative, a pattern that becomes evident when contrasted with the diphenylamine variant. Moreover, a notable feature across the **TDF** series is the increase in conjugation compared to the **TMF** series, manifesting as a bathochromic shift in the abortion bands. Overall, for all compounds examined, the absorption behavior aligns with that of previously reported fluorene‐triarylamine derivatives,[^36^, ^37^] which are typically characterized by two main bands: the less energetic one exhibiting lower absorbance than its counterpart.

When comparing the UV‐Vis spectra of derivatives in THF solution to those of films on quartz substrates (Figure 2b), a notable bathochromic shift is observed, although the band shapes remain similar. The quartz films, benefiting from a broader optical window, reveal an additional band below 300 nm for all derivatives. In the case of the diphenyl derivatives, this extra band exhibits strong absorption, mirroring the same band observed in the solution. Specifically, for **TMF‐1**, this refers to the bands at 301 nm and 390 nm, and for **TDF‐1**, at 296 nm and 387 nm. Interestingly, in the compound **TDF‐2**, which is presumed to have higher conjugation due to the addition of another fluorene with its donor units, the bands exhibit a hypsochromic shift. For derivatives containing the iminodibenzyl unit, the additional highest energetic band displays lower absorption compared to the subsequent lower energetic band. This pattern is also evident in the iminodibenzyl analogs; for **TMF‐2**, the two highest energetic bands are at longer wavelengths compared to those in **TDF‐2**. However, it's noteworthy that the last band in **TDF‐2**, at 431 nm, shows a more pronounced red shift compared to **TMF‐2**, which is at 412 nm.

The emission maxima in solution for the **TMF** and **TDF** series cover a range of wavelengths from 427 to 498 nm. Notably, derivatives featuring diphenylamine exhibit smaller Stokes shifts compared to those with iminodibenzyl. In general, the order of emission wavelengths is as follows: **TDF‐2** (498 nm)>**TMF‐2** (458 nm)>**TDF‐1** (428 nm)>**TMF‐1** (427 nm). When examining the emission properties in thin films, the derivatives with diphenylamine (**TMF‐1** and **TDF‐1**) show an emission peak at 650 nm. In contrast, those containing iminodibenzyl exhibit a noticeable difference; **TMF‐2** emits at 638 nm, which is 10 nm shorter than the emission of **TDF‐2** at 628 nm.

The electrochemical properties of the derivatives were investigated through the application of cyclic voltammetry (CV) and differential pulse voltammetry (DPV), as depicted in Figure 2b. It was found that each derivative exhibited multiple oxidation potentials, a phenomenon indicative of the existence of several electron‐donating units. These compounds are characterized by a high degree of reversibility in their oxidation reactions, with each showing three oxidation potentials. Notably, **TMF‐1** is an exception, exhibiting a fourth oxidation potential that appears to be irreversible. Interestingly, none of the compounds exhibit reduction potentials, which aligns with the absence of an electron‐accepting moiety in their structures. Among these compounds, the iminodibenzyl derivatives stand out due to their relatively lower first oxidation potential, especially when compared to the diphenylamine variants, which are about 20 mV higher. This observation suggests that incorporating iminodibenzyl into the structure lowers the oxidation potential. However, adding more electron‐donating units does not further decrease the oxidation potential; rather, it results in a slight increase. This phenomenon could be attributed to suboptimal electronic communication among the donor units, potentially leading to a distorted molecular geometry.

The HOMO and LUMO levels were determined using data from cyclic voltammetry (CV) and optical band gaps (E_0–0_) in solution. The HOMO level values were calculated using the formula E_HOMO_=4.4+E^ox^_1_, where 4.4 eV is the energy level of ferrocene in vacuo. Through these calculations, the HOMO energy levels for **TMF‐1**–**2** and **TMD‐1**–**2** were estimated to be −4.84, −4.82, −4.85, and −4.83 eV, respectively. Due to the alignment of these HOMO levels with the valence band edge of the double‐cation Cs _0.17_FA_0.83_PbI_3_ perovskite (approximately −5.68 eV), efficient hole extraction from the perovskite to the derivatives is anticipated. Furthermore, the LUMO energy levels were determined using the formula E_LUMO_=E_HOMO_+E_0–0_. As a result, the LUMO energy levels for the compounds **TMF‐1**–**2** and **TMD‐1**–**2** were found to be −1.78, −1.98, −1.79, and −2.11 eV, respectively. It was observed that the LUMO levels of the compounds exhibited a difference greater than 1 eV compared to the conduction band of the perovskite, indicative of their potential to effectively block back‐electron transfer.^[23]^

To gain deeper insight into the electrical properties of the series, work function (WF) measurements were performed, as shown in **Figure** **S19**. The WF values for **TMF‐1**, **TMF‐2**, **TDF‐1**, and **TDF‐2** were determined to be 4547, 4441, 4464, and 4513 meV, respectively. These WF measurements are consistent with the calculated HOMO energy levels, reinforcing the hypothesis that the HTMs could effectively extract holes from the perovskite. This is attributed to the favorable energy offset between the HOMO levels of the HTMs and the valence band of the perovskite. Although the WF values are similar, slight variations could impact hole extraction dynamics and, consequently, device performance. ^[38,39]^

The thermal properties of the synthesized molecules were meticulously assessed using thermogravimetric analysis (TGA) and differential scanning calorimetry (DSC), under a nitrogen atmosphere, as depicted in Figures 2c–d. Across the board, all compounds exhibited commendable thermal stability, maintaining their integrity at temperatures exceeding 300 °C. Notably, **TMF‐1** stood out for its exceptional thermal resilience, only beginning to degrade at a remarkable 384 °C, whereas **TMF‐2** demonstrated a somewhat lower thermal resistance, with degradation initiating at 327 °C. It was observed that derivatives incorporating iminodibenzyl tended to exhibit lower thermal resistance. In addition, DSC analysis revealed interesting morphological characteristics, particularly in **TMD‐1**, which exhibited a glass transition temperature of 112.6 °C. This was closely followed by **TMD‐2** at 105.5 °C, contrasting with the **TMF‐1**–**2** materials, which displayed lower glass transition temperatures of 87.5 °C and 81.8 °C, respectively.

The thin film samples were subjected to UVC irradiation (254 nm, 4.9 eV) in a nitrogen atmosphere for 50 hours to assess their response to high energy light irradiation. To minimize potential variation in degradation due to differences in light exposure and material absorbance at UVC wavelengths, the analysis focused on the number of absorbed UVC photons per square centimeter versus absorbance (details on the calculation are given in Figure S20), as depicted in Figure 2e (the progression of changes in the absorption spectra of the respective compounds can be seen in Figure S21). Initially, **TDF‐1** appeared to be the most photostable material. However, **TDF‐2** maintained relative stability up to 8×10^20^ absorbed photons, surpassing **TDF‐1**, which continued to degrade at a constant rate. Beyond the threshold of 1×10^21^ photons, **TMF‐2** began to slightly outperform **TDF‐1** in terms of photostability. In contrast, **TMF‐1** exhibited a high rate of photodegradation, rendering it the most vulnerable material to UVC light among the tested compounds. The degradation order can thus be summarized as **TDF‐2**>**TMF‐2**>**TDF‐1**>**TMF‐1**. This pattern suggests that the inclusion of iminodibenzyl significantly enhances photostability compared to diphenylamine within the same core molecular structure. Furthermore, the addition of a second fluorene moiety appears to bolster the photostability of the arylamines present in the molecules. These findings align with established models of semiconductor resistance under high‐energy radiation conditions, as discussed in the literature. ^[40]^

The contact angles of 12 μL water droplets on films of the **TMF** and **TDF** series over ITO substrates were measured to assess the hydrophobicity of the materials, as shown in Figure S22. All ITO substrates coated with different HTMs exhibited contact angles above 67.78°, which was the recorded value for the uncoated ITO substrate. The contact angles were observed to follow the trend: **TMF‐2** (81.79°)>**TDF‐2** (79.52°)>**TMF‐1** (78.80°)>**TDF‐1** (77.81°). These results indicate that materials with iminodibenzyl as end‐capping moieties are more hydrophobic than those with diphenylamine. Interestingly, the molecules with only one unit of fluorene exhibit better hydrophobicity. When compared with the reference material, the polymer PTAA has shown the highest hidrophovicity with a contact angle of 86.44°.

Ab initio DFT quantum chemical calculations have been performed on the four compounds under study. Firstly, geometry optimizations were performed at the B3LYP/6‐31G(d) level, in THF solution. The optimized geometries are displayed in Figure 1. Inspection of the geometries reveal some differences in the torsions around the aromatic‐N bonds in the arylamine phenyl units. More specifically, in **TMF‐1** and **TDF‐1** the dihedrals associated with these bonds present values within a short range of 130°–140° (the same as in the triarylamine units), while for **TMF‐2** these torsions take values between 100° and 120°, and in **TDF‐2** a disparity of values is adopted (from 100° to 160°). These differences reflect the restrictions on rotation generated by the iminodibenzyl moieties in **TMF‐2** and **TDF‐2**, that prevent these torsions to adopt more energetically favorable values as in **TMF‐1** and **TDF‐1**. As mentioned previously, such restrictions may explain the features of the corresponding UV‐Vis spectra.

Regarding the charge distribution in these compounds, the Mulliken population analysis reveals almost identical atomic charge values in the four compounds, suggesting that the electron donor character of the substituents does not lead to significant differences in those charge distributions.

**Figure 3:**
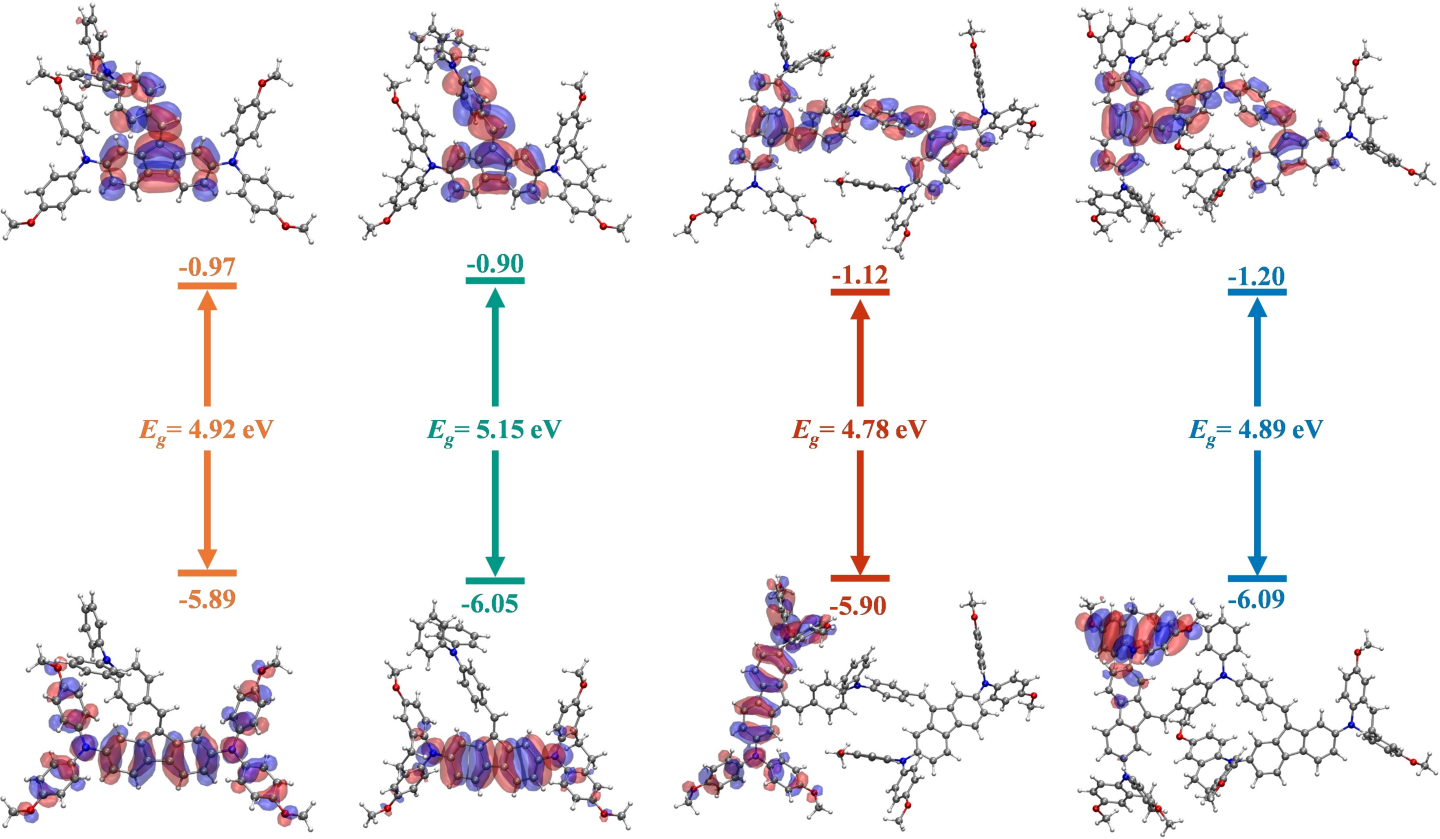
Frontier molecular orbitals diagram calculated at the CAM‐B3LYP/6‐311G(d,p) level in THF and plotted versus vacuum before contact for the TMF and TDF series proposed as HTMs.

The frontier molecular orbitals with their theoretical energy values are displayed in Figure 3 and Figure S23. For **TMF‐1** and **TDF‐1**, the HOMO is distributed through the fluorene and diphenylamine units, illustrating the conjugation of these moieties. Conversely, the HOMO for **TMF‐2** and **TDF‐2** are solely located on the fluorene or one iminodibenzyl unit, respectively. The LUMO or the four compounds involve the fluorene units and part of the tribenzyl units.

The vertical ionization energy for the four compounds have been computed with different combinations of functionals and basis sets in THF solution. In general, the four compounds present similar values for the computed ionization energy. More specifically, calculations with the CAM‐B3LYP functional and the 6‐311G(d,p) basis set provide slightly lower values for the iminodibenzyl derivatives (5.080 eV for **TMF‐2**, 5.052 eV for **TDF‐2**) than for the corresponding diphenylamine counterparts (5.081 eV for **TMF‐1**, 5.090 eV for **TDF‐1**), in agreement with experimental observations (Figure S24). However, the results at other levels of calculation do not follow the same trend, for instance at the DEF2/TZVPP level (**TMF‐1**: 4.560 eV; **TMF‐2**: 4.683 eV; **TDF‐1**: 4.563 eV; **TDF‐2**: 4.753 eV). It should be noted that previous works on several NFA materials have found significant deviations between DFT calculated and experimental ionization energies.^[41]^

### Photovoltaic Properties of TMF and TDF Series

An important characteristic of materials intended as Hole Transport Materials (HTMs) in inverted architecture or p‐i‐n structures is their orthogonal processability with the perovskite layer. To gain insight into this property, each HTM solution, at a concentration of 3 mg/mL in chlorobenzene, was deposited onto indium tin oxide (ITO)/glass substrates. A volume of 70 μL of the solution was applied to the ITO surface and then spin‐coated at 5000 rpm for 30 seconds before being annealed at 100°C for 10 minutes. Subsequently, 70 μL of a solvent mixture consisting of dimethylformamide (DMF) and dimethyl sulfoxide (DMSO) in a 4:1 volume ratio was layered over the initial HTM coating, spin‐coated under the same conditions, and similarly annealed. The procedure is graphically represented in **Figure** **S26**. Scanning electron microscopy (SEM) images, shown in **Figures** **S27–S30**, indicated that the addition of the solvent mixture slightly washed away some of the film, although the overall film morphology was preserved, effectively maintaining coverage of the ITO surface (**Figure** **S31**). It was noted that before the washing step, more agglomeration occurred within the films; after washing, the film distribution appeared more uniform, likely due to the redissolution of the film. Darker regions in the images were identified as areas with higher material accumulation. These observations suggest that the TMF and TDF series exhibit effective orthogonal processability, making them suitable for use in inverted perovskite solar cells.

**Figure 4:**
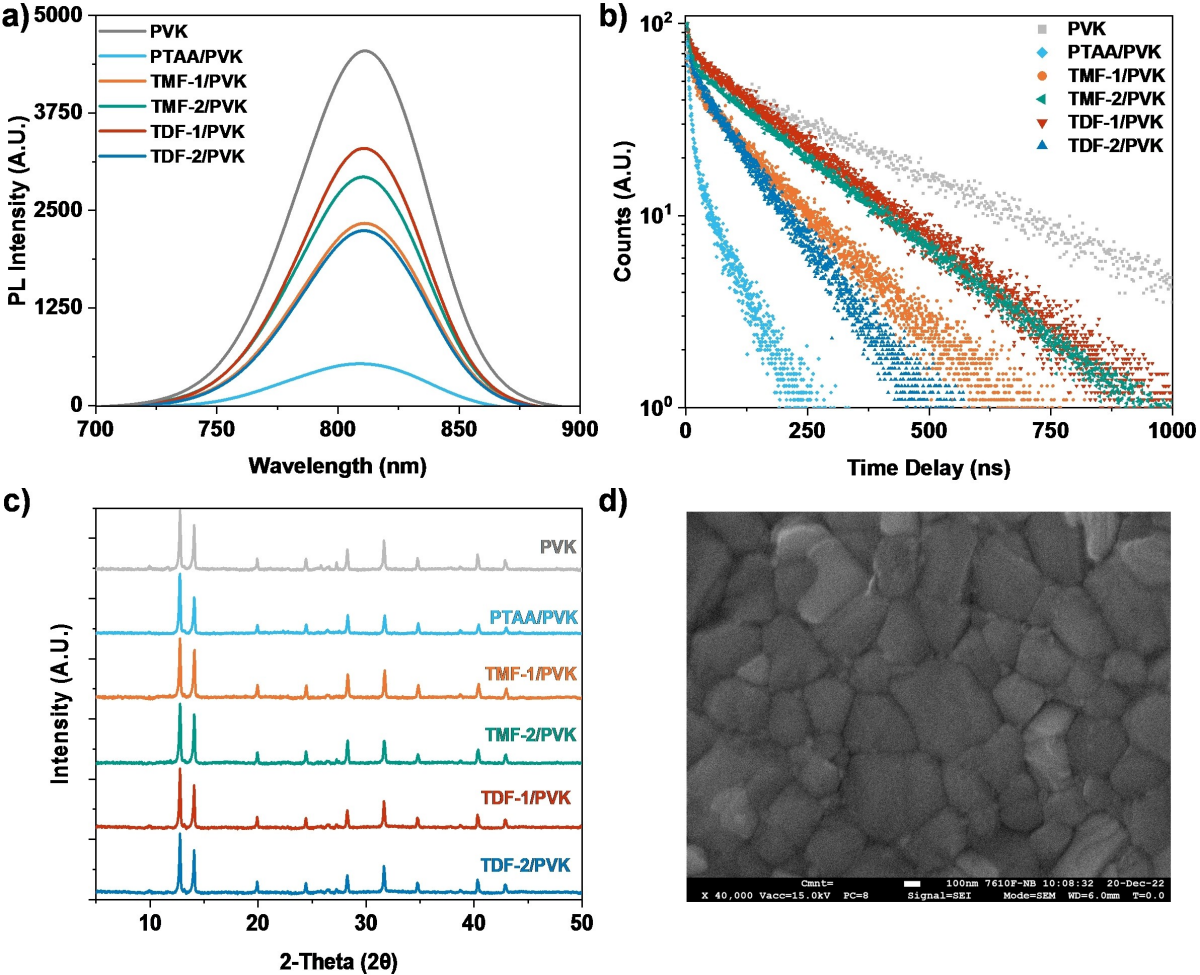
a) Steady‐state photoluminescence spectra, b) time‐resolved photoluminescence decay, the black line is the fitting result of each decay curve. c) XRD patterns of a bare Cs_0.17_FA_0.83_ PbI_3_ perovskite film and bilayered perovskite films with TDF and TPF series on glass substrate. d) Surface topographic SEM images of the perovskite films deposited on **TDF‐2**.

To explore the potential application of these derivatives in inverted planar perovskite solar cells, they were spin‐coated onto glass substrates. Subsequently, a perovskite layer of Cs_0.17_FA_0.83_ PbI_3_ was prepared on top using the antisolvent quenching method. The efficiency of hole extraction and collection from the perovskite layer to the compounds was investigated. This was accomplished using steady‐state photoluminescence (STPL) and time‐resolved photoluminescence (TrPL) analyses, the results of which are presented in Figure 4a–b.

Investigating the photoluminescence characteristics of bare perovskite films on glass substrates reveals a pronounced fluorescence emission at 811 nm under excitation with a 402 nm laser. This inherent luminescence characteristic undergoes a notable attenuation upon the incorporation of **TDF‐2**, evidenced by a 50.71 % reduction in photoluminescence (PL) intensity, indicative of substantial quenching phenomena. This phenomenon was comparatively analyzed with other derivatives, such as **TMF‐1**, which exhibited a 48.65 % reduction in PL intensity. Following this, **TMF‐2** and **TDF‐1** showed reductions of 35.64 % and 27.52 %, respectively. Interestingly, these findings somewhat mirror the trends observed in the oxidation potentials, with the notable exception that **TDF‐2** demonstrated slightly superior quenching compared to **TMF‐1**, potentially due to enhanced morphological stability.[^42^, ^43^, ^44^] Despite these findings, it is critical to note that these reductions, while significant, do not parallel the efficiency demonstrated by the reference standard PTAA, which shows an 88.23 % quenching in PL intensity.

To shed light on the dynamics of carrier extraction from the perovskite to the layers of the new materials, time‐resolved photoluminescence (TRPL) spectra were used (as illustrated in Figure 4b). These spectra are interpreted using the biexponential decay model developed by Kirchartz et al.[^45^, ^46^, ^47^] In this model, the first decay component (τ_1_) indicates the efficiency of carrier extraction by the hole transport material (HTM), while the second decay component (τ_2_) is associated with slower non‐radiative recombination processes, predominantly Shockley‐Read‐Hall (SRH) recombination, providing insight into recombination losses at the interfaces. The bare perovskite layer displays extended decay times (τ_1_=8.53 ns, τ_2_=378.94 ns), a characteristic attributable to the lack of a charge extraction interface. In contrast, the bilayer configurations of perovskite with the investigated derivatives demonstrate a consistent reduction in charge carrier lifetimes. The most notable reduction is observed with **TDF‐2**, which presents the shortest decay times in the series (τ_1_=6.50 ns, τ_2_=152.36 ns), in concordance with the trend illustrated in the STPL. The PTAA/perovskite bilayer shows the shortest decay times (τ_1_=5.37 ns, τ_2_=68.54 ns), indicating efficient carrier extraction and reduced recombination losses, potentially due to less effective passivation or less defect density at the interface.

Given that photoluminescence PL intensity and carrier lifetime are influenced by factors such as thin film trap density, interfacial defects, and energy level mismatches, the film morphology of perovskite on the derivatives was analyzed.^[48]^ X‐ray diffraction (XRD) patterns of HTM/perovskite films, presented in Figure 4c, were obtained under optimal device conditions. The analysis of these patterns reveals nearly equivalent intensities across all distinctive peaks in the perovskite/HTM films, suggesting a similar crystallinity across the perovskites on different HTMs. Distinct diffraction peaks at 14.09°, 19.98°, 24.40°, 28.35°, 31.76°, 34.97°, 40.53°, and 43.14° are identified, corresponding to the (111), (120), (012), (222), (231), (030), (240), and (333) planes of the perovskite structure. Additionally, peaks observed at 12.89° and a minor one at 38.80° are assignable to the (001) and (003) lattice planes of PbI_2_, respectively. Together with the on‐top SEM images (Figure 4d; Figure S32–S36, Supporting Information) of the PTAA/and HTM/perovskite bilayers, this reveals homogeneity in the perovskite morphology regardless of the HTM. Therefore, the lower PL intensity and shorter carrier lifetimes observed in previous experiments could be attributed to efficient charge separation from the perovskite layer to the derivatives. Nevertheless, it is important to note that these measurements are performed under open‐circuit conditions, meaning that no actual charge carriers are extracted from the semidevice. Under these conditions, a partial polarization of the charge distribution occurs, and the reduction in PL intensity and lifetime for a bilayer compared to a bare film could be influenced by the enhancement of nonradiative losses. ^[46,49]^

**Figure 5:**
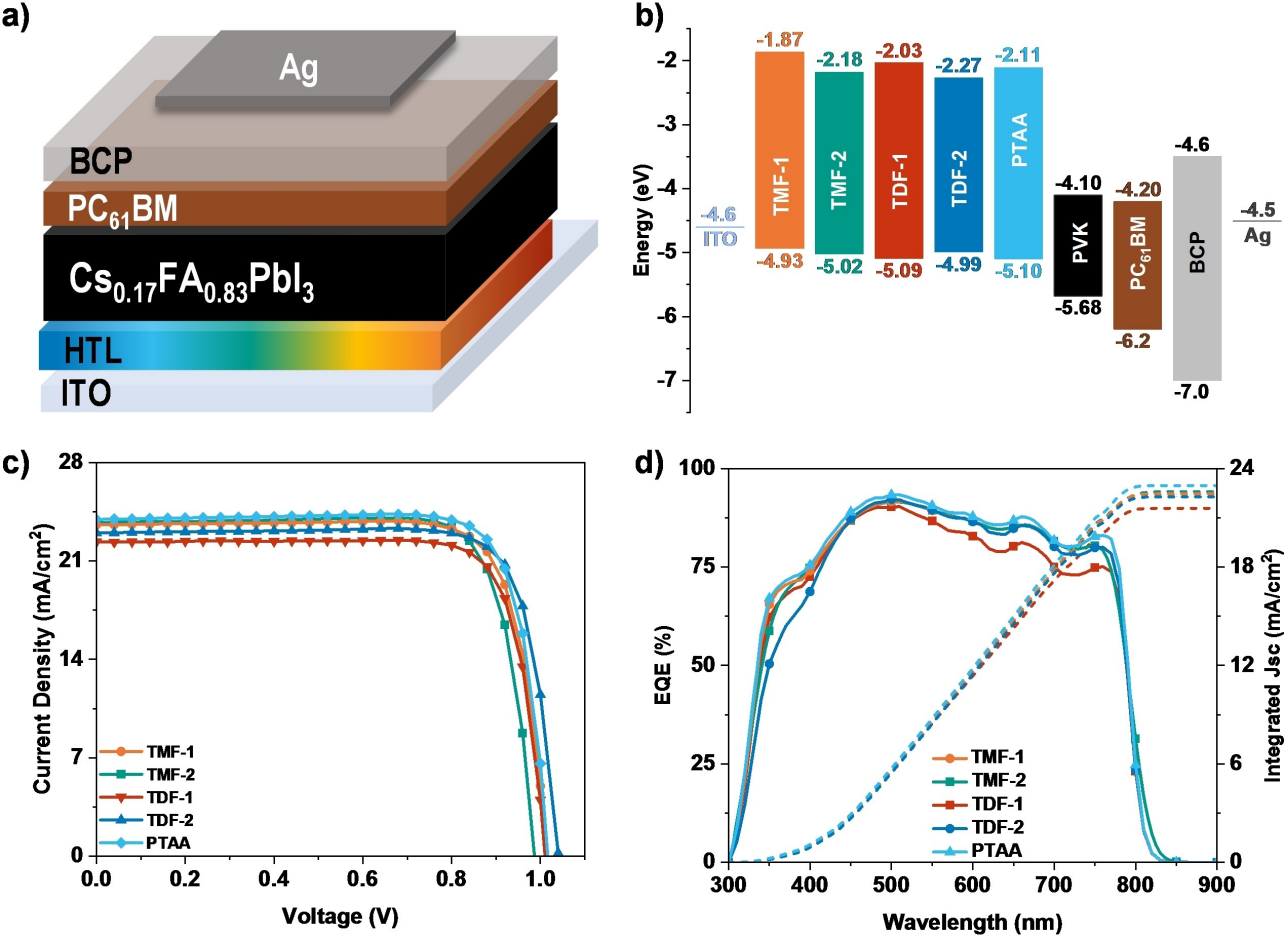
a) Schematic representation of planar *p*‐*i*‐*n* PSC device geometry and b) their corresponding energy diagrams utilized in this work. c) *J*‐*V* characteristic curves under standard AM 1.5G illumination conditions with HTMs and d) EQE spectra of devices from (c) and the integrated *J*_SC_ obtained from EQE.

The photovoltaic performance of the derivatives as hole transport materials (HTMs) was evaluated through the preparation of solution‐processed perovskite solar cells (PSCs), which were compared to those using the widely recognized reference HTM, PTAA. These PSCs were fabricated in an inverted planar p‐i‐n configuration, following the architecture of Glass/ITO/HTM/Perovskite/PC_61_BM/BCP/Ag (100 nm), with Cs_0.17_FA_0.83_PbI_3_ serving as the light‐harvesting material, as depicted in Figure 5a. The HTMs were deposited by spin‐coating from a chlorobenzene solution at a concentration of 2 mg mL^−1^, without the inclusion of additives or dopants. Further details on device fabrication are provided in the Supplementary Information. Illustrated in Figure 5b is the schematic of the energy levels of the various components within the PSCs. It was observed that the new HTMs displayed a favorable band alignment between their HOMO energy levels and the valence band edge of the double‐cation perovskite, suggesting potential for effective hole‐extraction and efficient electron‐blocking. Additionally, good solubility of all derivatives in chlorobenzene was noted, which is advantageous for achieving films with desirable morphology and uniform surface coverage.

Under standard AM 1.5 G illumination conditions, the photocurrent density‐voltage (*J–V*) characteristics of the devices were measured at an intensity of 100 mW cm^−2^. The resulting champion performance curves and associated data are showcased in Figure 5c, Figure S37a–e (Supporting Information), and Table 2. Devices based on **TDF‐2** achieved an open‐circuit voltage (V_OC_) of 1.04 V, a short‐circuit current (J_SC_) of 23.00 mA cm^−2^, and a fill factor (FF) of 0.81, culminating in the highest power conversion efficiency (PCE) of 19.38 %. In contrast, devices utilizing **TMF‐1** exhibited slightly lower efficiencies, with a peak PCE of 19.11 %. Devices incorporating **TMF‐2** reached a PCE of 18.83 %, and those with **TDF‐1** showed the lowest efficiency at 18.16 %. It was observed that these PCE values align with the quenching sequence **TDF‐2**>**TMF‐1**>**TMF‐2**>**TDF‐1**. However, consistent with expectations from previous experiments, none of the new materials outperformed the established benchmark set by PTAA, which recorded an open‐circuit voltage (V_OC_) of 1.02 V, a short‐circuit current (J_SC_) of 23.71 mA cm^−2^, a fill factor (FF) of 0.83, and a PCE of 20.20 %. The calculated J_SC_ values integrated from the external quantum efficiencies (EQEs), displayed consistency with the experimental observations across all cases, as illustrated in Figure 5d. Interestingly, these performance trends correspond to the WF values observed in **Figure** **S19**. **TMF‐1** and **TDF‐2** exhibited the highest WF values, which are consistent with their superior performance. However, **TMF‐1**, having a WF value closest to that of the ITO layer, might experience increased recombination with the ITO, potentially contributing to its slightly lower PCE compared to **TDF‐2**.

**Table 2:** Photovoltaic parameters of the champion solar cells containing the **TMF** and **TDF** series and **PTAA**.

	Scan Direction	V_OC_ [V]	J_SC_ [mA cm^−2^]	FF	PCE [%]
TMF‐1	Reverse Forward	1.01 1.01	23.57 22,91	0.80 0.80	19.11 18.50
TMF‐2	Reverse Forward	0.99 0.99	23.74 23.04	0.80 0.79	18.83 18.06
TDF‐1	Reverse Forward	1.01 1.00	22.33 21.48	0.80 0.82	18.16 17.72
TDF‐2	Reverse Forward	1,04 1.04	23.00 23.16	0.81 0.80	19.38 19.20
PTAA	Reverse Forward	1.02 1.03	23.71 22.74	0.83 0.82	20.20 19.30

*J–V* curves of hole‐only devices of the **TMF‐1**–**2** and **TDF‐1**–**2** were measured in the dark, and the hole mobility (*μ*_h_) was determined using the space‐charge limited current method (SCLC) (Figure S37f in the Supporting Information). The hole mobility values were determined to be 5.08×10^−5^, 4.84×10^−5^, 4.20×10^−5^, and 5.52×10^−5^ cm^2^ V^−1^ s^−1^, respectively, following the same tendency as the PL and PCE. However, the PTAA presented the highest value between the materials studied (2.60×10^−4^ cm^2^ V^−1^ s^−1^). These results also help attribute the effects observed in the PL measurements shown in **Figure** **4a‐b** to the extraction capabilities of these materials.

In literature, there are three accepted methods for analyzing the hysteresis index (HI): based on the relationship between Voc and Jsc[^50^, ^51^], PCE[^52^, ^53^], and area under the J‐V curve^[54]^,. **Figure** **S38** illustrates these methods for the curves shown in **Figure** **S37**. Under all three methods, devices based on **TDF‐2** exhibit the smallest HI, suggesting minimal ion migration and reduced charge trapping at the HTM/perovskite interface. In contrast, devices based on PTAA consistently show the highest HI across all three methods, indicating potential issues such as slower ionic movement or charge trapping.

The long‐term dark stability of PSCs based on **TMF‐1**–**2** and **TDF‐1**–**2** was evaluated under standard AM 1.5 G simulated sunlight, using non‐encapsulated devices maintained in ambient air with a relative humidity of 27.5 % ±2.5 % (Figure S39). After 264 hours, the PTAA‐ and **TDF‐02**‐based devices retained 96 % and 94 % of their initial PCE, respectively. **TMF‐1** followed closely, maintaining 92 % of its initial performance, while **TMF‐2** and **TDF‐1** showed a decrease to approximately 80 % of their original PCE. This degradation trend persisted over 400 hours, suggesting that significant further changes were unlikely and that the aging results could be extrapolated. Notably, the stability trend mirrors the efficiency trend, indicating that these results may be attributable to parallel factors.

**Figure 6:**
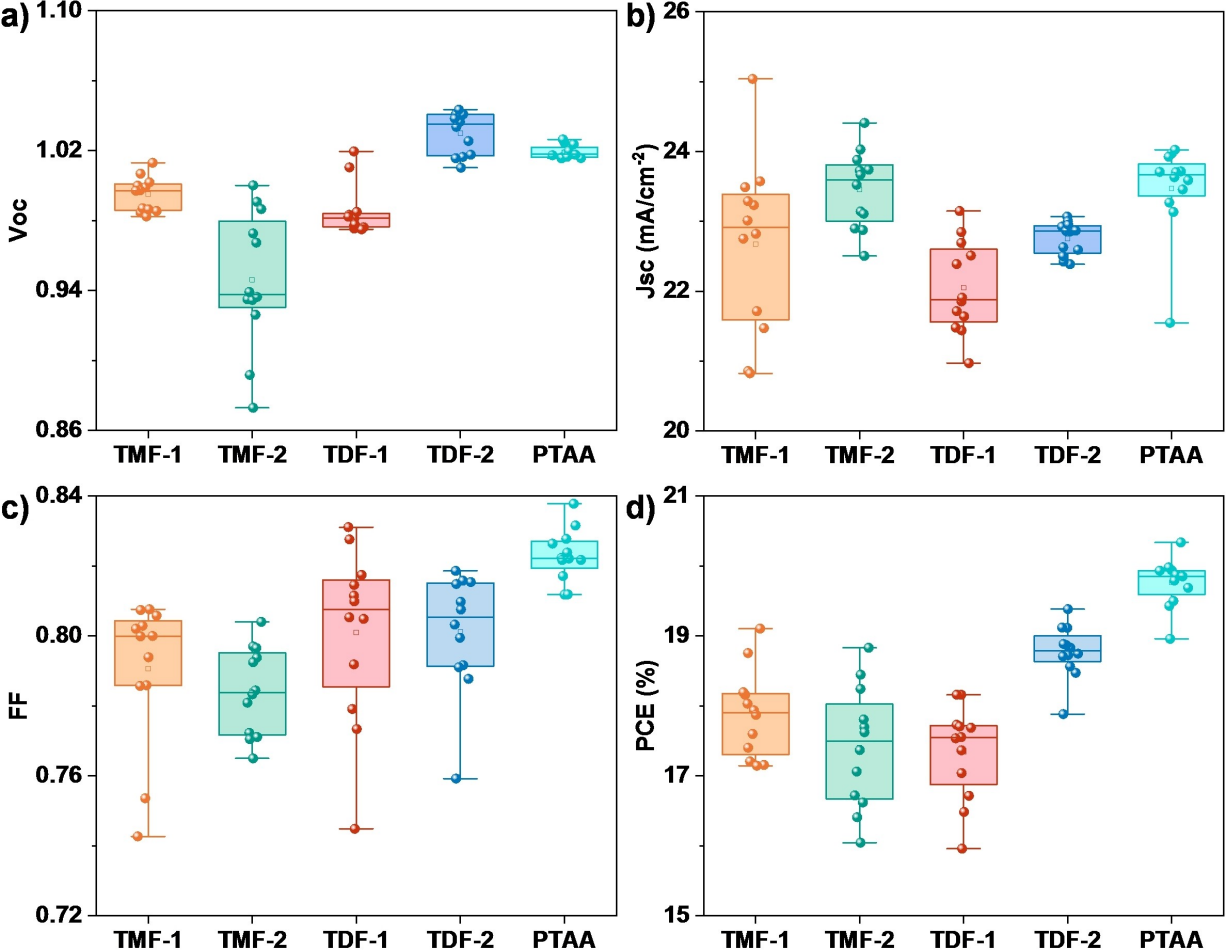
Statistical distribution of a) open circuit voltage (V_OC_), b) short circuit current density (*J*_SC_), c) Fill Factor (FF) and d) power conversion efficiency (PCE) for 12 devices incorporating **TMF‐1**–**2** and **TDF‐1**–**2** as HTMs in planar PSCs.

Figure 6 presents the statistical distribution of various photovoltaic parameters across 12 planar devices for each derivative, alongside the reference PTAA. The distribution plots clearly indicate that in terms of open‐circuit voltage (Voc), **TDF‐2** surpasses not only the other derivatives but also the reference PTAA. Interestingly, **TMF‐2** exhibits higher values for short‐circuit current (Jsc), closely rivaling those of PTAA. Regarding the fill factor (FF), none of the materials exceed the performance of the benchmark PTAA, with the derivatives displaying comparable FF values among themselves. The average power conversion efficiency (PCE) values align with those observed in the individual ′champion’ devices. Notably, **TDF‐2** demonstrates greater consistency in its PCE data, suggesting more uniform performance across multiple devices.

## Conclusions

In this study, four new hole transport materials (HTMs), **TMF‐1**–**2** and **TDF‐1**–**2**, featuring triphenylamine and fluorene as the main core, and dimethoxydiphenylamine and dimethoxy iminodibenzyl as end‐capping groups, were synthesized using Vielsmeyer‐Hack, Knoevenagel, and Buchwald‐Hartwig reactions. Their optical, electronic, and thermal properties were comprehensively characterized through UV‐Vis spectroscopy, fluorescence, cyclic and differential pulse voltammetry, thermogravimetric analysis (TGA), differential scanning calorimetry (DSC), and photodegradation studies, supplemented by theoretical HOMO and LUMO mapping. It was discovered that the iminodibenzyl moiety offers lower oxidation potentials, enhanced photostability, and a narrower bandgap compared to diphenylamine, making it a promising donor unit for HTMs. However, its inclusion was observed to reduce thermal resistance. Although none of the synthesized HTMs outperformed the reference material PTAA in terms of PCE (20.20 %) for Cs_0.17_FA_0.83_PbI_3_‐based perovskite solar cells (PSCs), all the materials demonstrated power conversion efficiency (PCE) values that were relatively close to one another. Notably, **TDF‐2**, containing iminodibenzyl, exhibited the most promising performance with a PCE of 19.38 %, while **TDF‐1**, with the lowest efficiency, recorded a PCE of 18.16 %. These findings highlight the potential of iminodibenzyl as a noteworthy contributor to the development of photovoltaic materials.

## Supporting Information Summary

The authors have cited additional references within the Supporting Information.[^55^, ^56^, ^57^, ^58^, ^59^, ^60^]

## Conflict of Interests

The authors declare no conflict of interest.

1

## Supporting information

As a service to our authors and readers, this journal provides supporting information supplied by the authors. Such materials are peer reviewed and may be re‐organized for online delivery, but are not copy‐edited or typeset. Technical support issues arising from supporting information (other than missing files) should be addressed to the authors.

Supporting Information

## Data Availability

The data that support the findings of this study are available from the corresponding author upon reasonable request.
