# Supplementary material for: Comparative Study of Iminodibenzyl and Diphenylamine Derivatives as Hole Transport Materials in Inverted Perovskite Solar Cells
Source: Chemistry. 2025 Jan 28;31(13):e202404251. doi: 10.1002/chem.202404251 (PMC11874900; doi:10.1002/chem.202404251)
Supplement: Supplementary file 1 — Supporting Information [file CHEM-31-e202404251-s001.pdf]

Chemistry–A European Journal

Supporting Information

Comparative Study of Iminodibenzyl and Diphenylamine Derivatives as Hole Transport Materials in Inverted Perovskite Solar Cells

Mauricio Caicedo-Reina, Juan S. Rocha-Ortiz,* Jianchang Wu, Andreas J. Bornschlegl, Salvador Leon, Anastasia Barabash, Jose Dario Perea, Yunuo Wang, Vanessa Arango-Marín, Alejandro Ortiz, Larry Lüer, Jens A. Hauch, Braulio Insuasty, and Christoph J. Brabec*

Comparative Study of Iminodibenzyl and Diphenylamine Derivatives as Hole Transport Materials in Inverted Perovskite Solar Cells

Mauricio Caicedo-Reina,[a] Juan S. Rocha-Ortiz,*[b-c] Jianchang Wu,[b-c] Andreas J. Bornschlegl,[c] Salvador Leon,[d] Anastasia Barabash,[c] Jose Dario Perea,[e] Yunuo Wang,[c] Vanessa Arango-Marín,[c] Alejandro Ortiz,[a] Larry Lüer,[c] Jens A. Hauch,[b] Braulio Insuasty,[a] Christoph J. Brabec*[b-c].

Dedication In memory of Prof. Luz Marina Jaramillo Gomez, whose teachings and guidance were invaluable

[a] M. Caicedo-Reina, A. Ortiz, B. Insuasty.
Department of Chemistry, Grupo de Investigación de Compuestos Heterocíclicos
Universidad del Valle
Calle 13 #100-00, 760032 Cali, Colombia

[b] J. S. Rocha-Ortiz, J. Wu, V. Arango-Marín, J. Hauch, C. J. Brabec.
Department of High Throughput Methods in Photovoltaics
Forschungszentrum Jülich GmbH, Helmholtz-Institute Erlangen-Nürnberg (HI ERN)
Immerwahrstraße 2, 91058 Erlangen, Germany
E-mail: j.rocha.ortiz@fz-juelich.de; c.brabec@fz-juelich.de

[c] J. S. Rocha-Ortiz, J. Wu, A. J. Bornschlegl, A. Barabash, V. Arango-Marín, Y. Wang, L. Lüer, C. J. Brabec.
Department of Materials Science and Engineering, Institute of Materials for Electronics and Energy Technology (i-MEET)
Friedrich-Alexander-Universität Erlangen-Nürnberg
Martensstraße 7, 91058 Erlangen, Germany

[d] S. Leon
Department of Chemical and Environmental Engineering, ETSIIM,
Universidad Politécnica de Madrid
José Gutiérrez Abascal 2, 28006 Madrid, Spain.

[e] J. D. Perea
Departament of biochemical Engineering
Universidad Icesi
Calle 18 # 122-135, 760031 Cali, Colombia.

Abstract: Perovskite solar cells (PSCs) have recently achieved over 26% power conversion efficiency, challenging the dominance of silicon-based alternatives. This progress is significantly driven by innovations in hole transport materials (HTMs), which notably influence the efficiency and stability of PSCs. However, conventional organic HTMs like PTAA, although highly efficient, suffer from thermal degradation, moisture ingress, and high cost. This study explores the potential of iminodibenzyl, a moiety known for its strong electron-donating capabilities in pharmaceutical applications, as a novel HTM. A series of fluorene-based derivatives incorporating iminodibenzyl (**TMF-2** and **TDF-2**) and diphenylamine (**TMF-1** and **TDF-1**) units were synthesized and characterized. The new HTMs demonstrated commendable optical, electrochemical, and thermal properties, as well as enhanced photostability. Among them, **TDF-2** achieved a power conversion efficiency (PCE) of 19.38%, the highest of the new materials. Although these efficiencies are slightly lower than the benchmark PTAA (20.20%), the study underscores the potential of iminodibenzyl to enhance photostability and increase HOMO levels, making it a promising candidate for future HTM development in PSCs.

Introduction

As the global quest for sustainable energy solutions intensifies, halide perovskite solar cells (PSCs) have emerged as a leading contender in the field. Demonstrating a remarkable trajectory, PSCs have advanced from less than 4 % power conversion efficiency in 2009 to over 20 % by 2015, showing that this type of materials are unique by featuring the fastest efficiency improvement realized for any solar cell technology.[1–3] PSCs are primarily configured as either conventional (n-i-p) or inverted (p-i-n) structures. The n-i-p structure typically outperforms p-i-n due to lower open-circuit voltage (VOC) and fill factor (FF) in the latter, largely caused by carrier recombination at the interfaces between the perovskite and the transport layers. However, the p-i-n PSCs present benefits over n-i-p PSCs including lower-temperature sintering (suitable for tandem cells and flexible devices), improved stability due to dopant-free hole transport layers (HTLs), reduced hysteresis in current-voltage characteristics, and potentially lower costs from using inexpensive back electrodes like Ag, Al, or Cu instead of Au. Some common HTLs are Spiro-OMeTAD for n-i-p and PTAA for p-i-n PSCs. [4,5]

Recent advancements in the inverted configuration have led to significant efficiency improvements, with certified values now surpassing 26%.[6] This astounding progress not only challenges

WILEY-VCH

the dominance of silicon-based alternatives but also underscores the pivotal role of innovative material development in driving the evolution of these devices.[7–9] Central to this evolution is the innovation in organic hole transport materials (HTMs), key components that greatly influence the efficiency and stability of inverted PSCs.[10] The organic HTM present advantages such as solution processing, tunable properties based on molecular design, and extended libraries with building blocks. Some organic HTM, like PTAA and Poly-TPD have achieved the highest efficiencies, they still present several challenges that affect device performance. These challenges include thermal degradation, moisture ingress, mechanical failure, sub-optimal conductivity, and significant recombination at the perovskite/HTL interface.[11] Furthermore, their application beyond research settings is limited due to their prohibitively high cost.

PTAA's structure includes polycyclic aromatic hydrocarbons (PAHs), widely used in organic semiconductors for their low cost, structural versatility, and tunable optoelectronic properties.[12–16] Additionally, the triarylamine (TAA) core, with its distinctive non-planar, propeller-like shape and sterically hindered *sp*² nitrogen, enhances electronic properties such as hyperconjugation. These features contribute to PTAA's high hole mobility, low oxidation potential, and reversible redox behavior, making it essential in organic photovoltaics, including light-emitting diodes and solar cells.[17–24] The importance of this core is underscored by molecules achieving similar or superior performance than PTAA, with one achieving a record 26.2% efficiency.[25] That research has shown that the combination of TAA [26] and fluorene leads to enhanced performance, surpassing that of the reference materials, while significantly reducing the harsh or costly conditions required for their synthesis.[27,28]

In this work we combined the features of these two units and ventured beyond conventional boundaries, exploring the potential of iminodibencyl, a compound predominantly known in pharmaceutical applications.[29,30] Although its application in HTM development has been limited thus far,[31] previous studies have demonstrated that its strong electron-donating capabilities can significantly enhance photovoltaic performance, particularly in sensitizers.[32,33] This research conducts a comparative analysis examining the impact of incorporating iminodibencyl versus diphenylamine into the HTM structure. The aim of this synthesis is to pioneer a new class of materials for use in photovoltaic technology, potentially broadening the horizons of sustainable energy sources.

Results and Discussion

Synthesis

The detailed synthetic route for the preparation of series **TMF** and **TDF** is depicted in **Figure 1** and **Scheme S1**. Initially, Aldehydes **2** and **3** were synthesized through a Vilsmeier-Haack reaction using triphenylamine (**1**) as the starting material. Subsequently, these aldehydes underwent a condensation reaction with 2,7-dibromofluorene in a basic aqueous-toluene medium, with tetrabutylammonium bromide serving as a phase transfer catalyst, resulting in the formation of the vinylenes **4** and **5**. These compounds were then subjected to a Buchwald-Hartwig amination reaction with 4,4′-dimethoxydiphenylamine **(7)**, which was facilitated by the use of $Pd_2(dba)_3$ as catalyst and XPhos as ligand, culminating in the synthesis of **TMF-1** and **TDF-1**. A parallel amination of vinylenes (**4**) and (**5**) with dimethoxyiminodibenzyl (**8**), under similar conditions, afforded **TMF-2** and **TDF-2**. All the detailed information about the synthetic procedures and characterization are available in the supporting information. A detailed cost analysis for the products of the TMF and TDF series is presented in **Tables S1-S8**, based on the methods employed in previous literature reports.[34,35]

The four target compounds from the **TMF** and **TDF** series were subjected to ab initio DFT quantum chemical calculations. Geometry optimizations were performed at the B3LYP/6-31G(d) level, in THF solution. The resulting conformations, displayed in **Figure 1**, do not exhibit significant differences regarding the geometry of the common units of the different compounds.

Optical, electrochemical and thermal properties

The effect of different chemical structures on their optical properties was studied by analyzing the absorption and fluorescence spectra of fluorene derivatives, presented in **Figure 2a**. These derivatives were dissolved in THF at a concentration of 1×10^{-5} M. **Table 1** summarizes the photophysical parameters. The optical bandgap was determined from the wavelength at the intercept between the fluorescence and absorption spectra (λ_{onset}), using the formula $E_{0\text{-}0} = 1240/\lambda_{onset}$.

In the UV-Vis spectra (**Figure 2a**), **TMF-1** displays a prominent absorption band at 382 nm, accompanied by a notable, more energetic shoulder near 430 nm. In contrast, **TMF-2** exhibits a sharp absorption at 355 nm, alongside a moderately intense band centered at 400 nm. This variation suggests a possible correlation: the shoulder observed in **TMF-1** at 430 nm may correspond to the 400 nm transition seen in **TMF-2**. This alignment hints at the involvement of the arylamine fragment. The underlying cause appears to be a state of greater rigidity in **TMF-2**, which restricts the rotation of the arylamine phenyls within the iminodibenzyl structure. This restriction results in the splitting of these bands. Conversely, such splitting is absent in **TMF-1**, where the band manifests instead as a shoulder.

Turning to the **TDF** series, their spectral characteristics show a notable resemblance to their monovinyl analogs. Specifically, **TDF-1** is characterized by a narrow yet intense absorption band at 383 nm, along with a less intense band at 434 nm that slightly merges with the main band. In the case of **TDF-2**, the spectrum reveals a smaller, somewhat broader band at 427 nm, accompanied by a more distinct band at 356 nm. This series also demonstrates the band splitting characteristic of the iminodibenzyl derivative, a pattern that becomes evident when contrasted with the diphenylamine variant. Moreover, a notable feature across the **TDF** series is the increase in conjugation compared to the **TMF** series, manifesting as a bathochromic shift in the abortion bands. Overall, for all compounds examined, the absorption behavior aligns with that of previously reported fluorene-triarylamine derivatives,[36,37] which are typically

WILEY-VCH

characterized by two main bands: the less energetic one exhibiting lower absorbance than its counterpart.

When comparing the UV-Vis spectra of derivatives in THF solution to those of films on quartz substrates (**Figure 2b**), a notable bathochromic shift is observed, although the band shapes remain similar. The quartz films, benefiting from a broader optical window, reveal an additional band below 300 nm for all derivatives. In the case of the diphenyl derivatives, this extra band exhibits strong absorption, mirroring the same band observed in the solution. Specifically, for **TMF-1**, this refers to the bands at 301 nm and 390 nm, and for **TDF-1**, at 296 nm and 387 nm. Interestingly, in the compound **TDF-2**, which is presumed to have higher conjugation due to the addition of another fluorene with its donor units, the bands exhibit a hypsochromic shift. For derivatives containing the iminodibenzyl unit, the additional highest energetic band displays lower absorption compared to the subsequent lower energetic band. This pattern is also evident in the iminodibenzyl analogs; for **TMF-2**, the two highest energetic bands are at longer wavelengths compared to those in **TDF-2**. However, it's noteworthy that the last band in **TDF-2**, at 431 nm, shows a more pronounced red shift compared to **TMF-2**, which is at 412 nm.

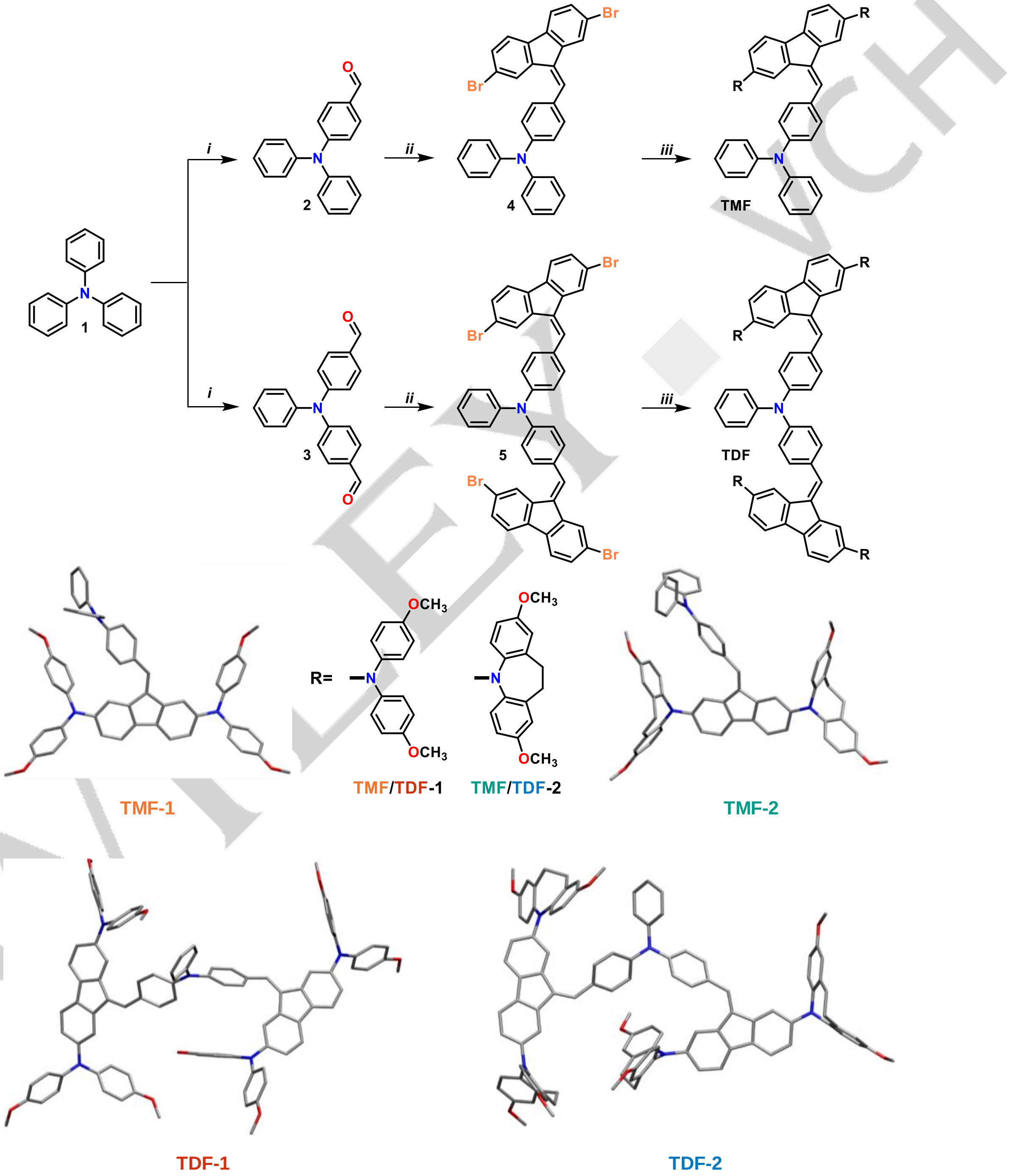

Figure 1. Figure caption. General procedure for the synthesis of **TMF** and **TDF** series and their DFT optimized geometries**.** Reagents and conditions: *i*) $POCl_3$, DMF, 80 °C, 8-12 h, *ii*) NaOH (40%), TBAB, toluene, 100 °C, 2 h, *iii*) **7** or **8**, $Pd_2(dba)_3$, XPhos, *t*-BuOK, toluene, 90 °C, 18 h.

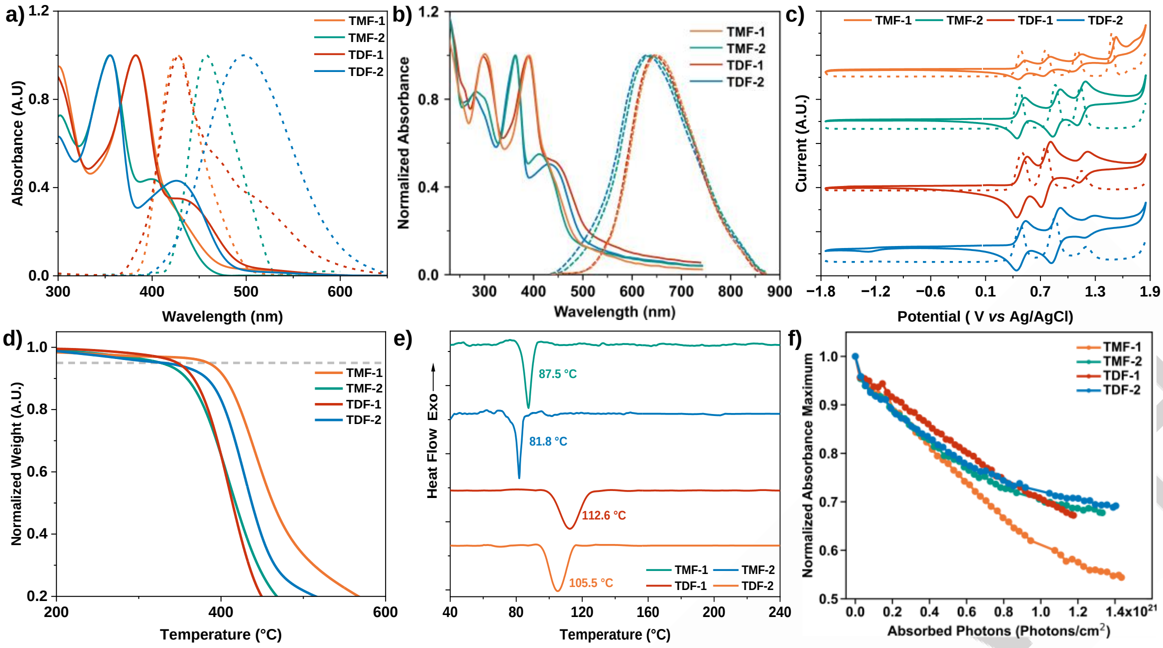

Figure 2. Normalized UV-Vis (solid line) and fluorescence spectra (dashed line) in **a)** THF solutions at 1×10^{-5} M and in **b)** thin film on a quartz substrate, **c)** cyclic (solid line) and differential pulse (dashed line) voltammograms of the anodic scan at 100 mV s^{-1} in dry 0.1 M $TBAPF_6$ CH_2Cl_2 solution at 21±1 °C (V *vs* Ag/AgCl), **d)** thermogravimetric analysis curves at 10 °C min^{-1} of heating rate and **e)** differential scanning calorimetry curves under nitrogen at a heating rate of 20 °C min^{-1} for the series **TMF** and **TDF**. **f)** Evolution of main absorbance peaks (see **Figure S21**) of the thin films on quartz substrates during UVC degradation over the number of absorbed UVC photons.

The emission maxima in solution for the **TMF** and **TDF** series cover a range of wavelengths from 427 to 498 nm. Notably, derivatives featuring diphenylamine exhibit smaller Stokes shifts compared to those with iminodibenzyl. In general, the order of emission wavelengths is as follows: **TDF-2** (498 nm) > **TMF-2** (458 nm) > **TDF-1** (428 nm) > **TMF-1** (427 nm). When examining the emission properties in thin films, the derivatives with diphenylamine (**TMF-1** and **TDF-1**) show an emission peak at 650 nm. In contrast, those containing iminodibenzyl exhibit a noticeable difference; **TMF-2** emits at 638 nm, which is 10 nm shorter than the emission of **TDF-2** at 628 nm.

Table 1. Optical, electrochemical and thermal properties of **TMF** and **TDF** derivatives.

	**λ max, abs. [nm]**		**λ max, em. (λ ex) [nm]**		E^{ox}_1 [b] [V]	E_{HOMO} [c] [eV]	$E_{0\text{-}0}$ [d] [eV]	E_{LUMO} [e] [eV]	T_{dec} [f] [ºC]
	THF	Film	THF	Film[a]					
**TMF-1**	383	301, 390	427 (383)	650	0.44	-4.84	3.06	-1.78	384
**TMF-2**	355	363	457 (399)	638	0.42	-4.82	2.84	-1.98	327
**TDF-1**	383	296, 387	428 (382)	650	0.45	-4.85	3.06	-1.79	347
**TDF-2**	356	362	498 (425)	628	0.43	-4.83	2.72	-2.11	329

[a] All films were exited with a laser at 402 nm. [b] Determined from differential pulse voltammetry peaks versus silver/silver chloride reference electrode (Ag/AgCl). [c] E_{HOMO} was calculated by E_{HOMO} (eV) = - (E^{ox}_1 + 4.4). [d] The optical bandgap was calculated from the onset of the lower energy absorption edge ($E_{0\text{-}0}$ (eV) = 1240/ λ_{onset}). [e] E_{LUMO} was calculated by E_{LUMO} (eV) = E_{HOMO} + $E_{0\text{-}0}$. [f] Decomposition temperature determined from TGA (5% weight loss under a N_2 atmosphere).

The electrochemical properties of the derivatives were investigated through the application of cyclic voltammetry (CV) and differential pulse voltammetry (DPV), as depicted in **Figure 2b**. It was found that each derivative exhibited multiple oxidation potentials, a phenomenon indicative of the existence of several electron-donating units. These compounds are characterized by a high degree of reversibility in their oxidation reactions, with each showing three oxidation potentials. Notably, **TMF-1** is an exception, exhibiting a fourth oxidation potential that appears to be irreversible. Interestingly, none of the compounds exhibit reduction potentials, which aligns with the absence of an electron-accepting moiety in their structures. Among these compounds, the iminodibenzyl derivatives stand out due to their relatively lower first oxidation potential, especially when compared to the diphenylamine variants, which are about 20 mV higher. This observation suggests that incorporating iminodibenzyl into the

structure lowers the oxidation potential. However, adding more electron-donating units does not further decrease the oxidation potential; rather, it results in a slight increase. This phenomenon could be attributed to suboptimal electronic communication among the donor units, potentially leading to a distorted molecular geometry.

The HOMO and LUMO levels were determined using data from cyclic voltammetry (CV) and optical band gaps (E_{0-0}) in solution. The HOMO level values were calculated using the formula $E_{HOMO} = 4.4 + E^{ox}_1$, where 4.4 eV is the energy level of ferrocene in vacuo. Through these calculations, the HOMO energy levels for **TMF-1-2** and **TDF-1-2** were estimated to be −4.84, −4.82, -4.85, and −4.83 eV, respectively. Due to the alignment of these HOMO levels with the valence band edge of the double-cation $Cs_{0.17}FA_{0.83}PbI_3$ perovskite (approximately −5.68 eV), efficient hole extraction from the perovskite to the derivatives is anticipated. Furthermore, the LUMO energy levels were determined using the formula $E_{LUMO} = E_{HOMO} + E_{0-0}$. As a result, the LUMO energy levels for the compounds **TMF-1-2** and **TDF-1-2** were found to be −1.78, −1.98, -1.79, and −2.11 eV, respectively. It was observed that the LUMO levels of the compounds exhibited a difference greater than 1 eV compared to the conduction band of the perovskite, indicative of their potential to effectively block back-electron transfer.[23]

To gain deeper insight into the electrical properties of the series, work function (WF) measurements were performed, as shown in **Figure S19**. The WF values for **TMF-1**, **TMF-2**, **TDF-1**, and **TDF-2** were determined to be 4547, 4441, 4464, and 4513 meV, respectively. These WF measurements are consistent with the calculated HOMO energy levels, reinforcing the hypothesis that the HTMs could effectively extract holes from the perovskite. This is attributed to the favorable energy offset between the HOMO levels of the HTMs and the valence band of the perovskite. Although the WF values are similar, slight variations could impact hole extraction dynamics and, consequently, device performance.[38,39]

The thermal properties of the synthesized molecules were meticulously assessed using thermogravimetric analysis (TGA) and differential scanning calorimetry (DSC), under a nitrogen atmosphere, as depicted in **Figures 2c-d**. Across the board, all compounds exhibited commendable thermal stability, maintaining their integrity at temperatures exceeding 300 °C. Notably, **TMF-1** stood out for its exceptional thermal resilience, only beginning to degrade at a remarkable 384 °C, whereas **TMF-2** demonstrated a somewhat lower thermal resistance, with degradation initiating at 327 °C. It was observed that derivatives incorporating iminodibenzyl tended to exhibit lower thermal resistance. In addition, DSC analysis revealed interesting morphological characteristics, particularly in **TDF-1**, which exhibited a glass transition temperature of 112.6 °C. This was closely followed by **TDF-2** at 105.5 °C, contrasting with the **TMF-1-2** materials, which displayed lower glass transition temperatures of 87.5 °C and 81.8 °C, respectively.

The thin film samples were subjected to UVC irradiation (254 nm, 4.9 eV) in a nitrogen atmosphere for 50 hours to assess their response to high energy light irradiation. To minimize potential variation in degradation due to differences in light exposure and material absorbance at UVC wavelengths, the analysis focused on the number of absorbed UVC photons per square centimeter versus absorbance (details on the calculation are given in **Figure S20**), as depicted in **Figure 2e** (the progression of changes in the absorption spectra of the respective compounds can be seen in **Figure S21**). Initially, **TDF-1** appeared to be the most photostable material. However, **TDF-2** maintained relative stability up to 8×10^{20} absorbed photons, surpassing **TDF-1**, which continued to degrade at a constant rate. Beyond the threshold of 1×10^{21} photons, **TMF-2** began to slightly outperform **TDF-1** in terms of photostability. In contrast, **TMF-1** exhibited a high rate of photodegradation, rendering it the most vulnerable material to UVC light among the tested compounds. The degradation order can thus be summarized as **TDF-2** > **TMF-2** > **TDF-1** > **TMF-1**. This pattern suggests that the inclusion of iminodibenzyl significantly enhances photostability compared to diphenylamine within the same core molecular structure. Furthermore, the addition of a second fluorene moiety appears to bolster the photostability of the arylamines present in the molecules. These findings align with established models of semiconductor resistance under high-energy radiation conditions, as discussed in the literature.[40]

The contact angles of 12 µL water droplets on films of the **TMF** and **TDF** series over ITO substrates were measured to assess the hydrophobicity of the materials, as shown in **Figure S22**. All ITO substrates coated with different HTMs exhibited contact angles above 67.78°, which was the recorded value for the uncoated ITO substrate. The contact angles were observed to follow the trend: **TMF-2** (81.79°) > **TDF-2** (79.52°) > **TMF-1** (78.80°) > **TDF-1** (77.81°). These results indicate that materials with iminodibenzyl as end-capping moieties are more hydrophobic than those with diphenylamine. Interestingly, the molecules with only one unit of fluorene exhibit better hydrophobicity. When compared with the reference material, the polymer PTAA has shown the highest hydrophobicity with a contact angle of 86.44°.

Ab initio DFT quantum chemical calculations have been performed on the four compounds under study. Firstly, geometry optimizations were performed at the B3LYP/6-31G(d) level, in THF solution. The optimized geometries are displayed in **Figure 1**. Inspection of the geometries reveal some differences in the torsions around the aromatic-N bonds in the arylamine phenyl units. More specifically, in **TMF-1** and **TDF-1** the dihedrals associated with these bonds present values within a short range of 130°-140° (the same as in the triarylamine units), while for **TMF-2** these torsions take values between 100° and 120°, and in **TDF-2** a disparity of values is adopted (from 100° to 160°). These differences reflect the restrictions on rotation generated by the iminodibenzyl moieties in **TMF-2** and **TDF-2**, that prevent these torsions to adopt more energetically favorable values as in **TMF-1** and **TDF-1**. As mentioned previously, such restrictions may explain the features of the corresponding UV-Vis spectra.

Regarding the charge distribution in these compounds, the Mulliken population analysis reveals almost identical atomic charge values in the four compounds, suggesting that the electron donor character of the substituents does not lead to significant differences in those charge distributions.

The frontier molecular orbitals with their theoretical energy values are displayed in **Figure 3 and Figure S23**. For **TMF-1** and **TDF-1**, the HOMO is distributed through the fluorene and

WILEY-VCH

diphenylamine units, illustrating the conjugation of these moieties. Conversely, the HOMO for **TMF-2** and **TDF-2** are solely located on the fluorene or one iminodibenzyl unit, respectively. The LUMO or the four compounds involve the fluorene units and part of the tribenzyl units.

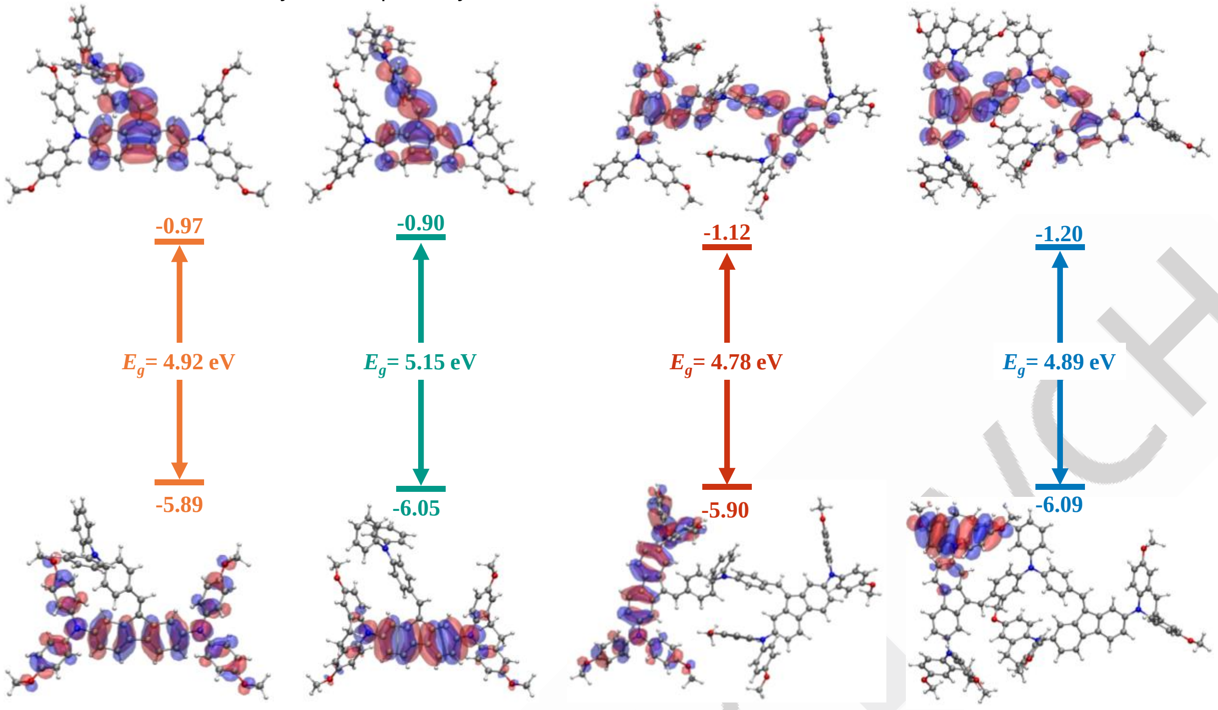

Figure 3. Frontier molecular orbitals diagram calculated at the CAM-B3LYP/6-311G(d,p) level in THF and plotted versus vacuum before contact for the TMF and TDF series proposed as HTMs.

The vertical ionization energy for the four compounds have been computed with different combinations of functionals and basis sets in THF solution. In general, the four compounds present similar values for the computed ionization energy. More specifically, calculations with the CAM-B3LYP functional and the 6-311G(d,p) basis set provide slightly lower values for the iminodibenzyl derivatives (5.080 eV for **TMF-2**, 5.052 eV for **TDF-2**) than for the corresponding diphenylamine counterparts (5.081 eV for **TMF-1**, 5.090 eV for **TDF-1**), in agreement with experimental observations (**Figure S24**). However, the results at other levels of calculation do not follow the same trend, for instance at the DEF2/TZVPP level (**TMF-1**: 4.560 eV; **TMF-2**: 4.683 eV; **TDF-1**: 4.563 eV; **TDF-2**: 4.753 eV). It should be noted that previous works on several NFA materials have found significant deviations between DFT calculated and experimental ionization energies.[41]

Photovoltaic Properties of TMF and TDF series

An important characteristic of materials intended as Hole Transport Materials (HTMs) in inverted architecture or p-i-n structures is their orthogonal processability with the perovskite layer. To gain insight into this property, each HTM solution, at a concentration of 3 mg/mL in chlorobenzene, was deposited onto indium tin oxide (ITO)/glass substrates. A volume of 70 µL of the solution was applied to the ITO surface and then spin-coated at 5000 rpm for 30 seconds before being annealed at 100°C for 10 minutes. Subsequently, 70 µL of a solvent mixture consisting of dimethylformamide (DMF) and dimethyl sulfoxide (DMSO) in a 4:1 volume ratio was layered over the initial HTM coating, spin-coated under the same conditions, and similarly annealed. The procedure is graphically represented in **Figure S26**. Scanning electron microscopy (SEM) images, shown in **Figures S27–S30**, indicated that the addition of the solvent mixture slightly washed away some of the film, although the overall film morphology was preserved, effectively maintaining coverage of the ITO surface (**Figure S31**). It was noted that before the washing step, more agglomeration occurred within the films; after washing, the film distribution appeared more uniform, likely due to the redissolution of the film. Darker regions in the images were identified as areas with higher material accumulation. These observations suggest that the TMF and TDF series exhibit effective orthogonal processability, making them suitable for use in inverted perovskite solar cells.

To explore the potential application of these derivatives in inverted planar perovskite solar cells, they were spin-coated onto glass substrates. Subsequently, a perovskite layer of $Cs_{0.17}FA_{0.83}PbI_3$ was prepared on top using the antisolvent quenching method. The efficiency of hole extraction and collection from the perovskite layer to the compounds was investigated. This was accomplished using steady-state photoluminescence (STPL) and time-resolved photoluminescence (TrPL) analyses, the results of which are presented in **Figure 4a-b**.

Investigating the photoluminescence characteristics of bare perovskite films on glass substrates reveals a pronounced fluorescence emission at 811 nm under excitation with a 402 nm laser. This inherent luminescence characteristic undergoes a notable attenuation upon the incorporation of **TDF-2**, evidenced by a 50.71% reduction in photoluminescence (PL) intensity, indicative of substantial quenching phenomena. This phenomenon was comparatively analyzed with other derivatives, such as **TMF-1**, which exhibited a 48.65% reduction in PL

intensity. Following this, **TMF-2** and **TDF-1** showed reductions of 35.64% and 27.52%, respectively. Interestingly, these findings somewhat mirror the trends observed in the oxidation potentials, with the notable exception that **TDF-2** demonstrated slightly superior quenching compared to **TMF-1**, potentially due to enhanced morphological stability.[42–44] Despite these findings, it is critical to note that these reductions, while significant, do not parallel the efficiency demonstrated by the reference standard PTAA, which shows an 88.23% quenching in PL intensity.

To shed light on the dynamics of carrier extraction from the perovskite to the layers of the new materials, time-resolved photoluminescence (TRPL) spectra were used (as illustrated in **Figure 4b**). These spectra are interpreted using the biexponential decay model developed by Kirchartz et al.[45–47] In this model, the first decay component (τ_1) indicates the efficiency of carrier extraction by the hole transport material (HTM), while the second decay component (τ_2) is associated with slower non-radiative recombination processes, predominantly Shockley-Read-Hall (SRH) recombination, providing insight into recombination losses at the interfaces. The bare perovskite layer displays extended decay times (τ_1 = 8.53 ns, τ_2 = 378.94 ns), a characteristic attributable to the lack of a charge extraction interface. In contrast, the bilayer configurations of perovskite with the investigated derivatives demonstrate a consistent reduction in charge carrier lifetimes. The most notable reduction is observed with **TDF-2**, which presents the shortest decay times in the series (τ_1 = 6.50 ns, τ_2 = 152.36 ns), in concordance with the trend illustrated in the STPL. The PTAA/perovskite bilayer shows the shortest decay times (τ_1 = 5.37 ns, τ_2 = 68.54 ns), indicating efficient carrier extraction and reduced recombination losses, potentially due to more effective passivation or less defect density at the interface.

Given that photoluminescence PL intensity and carrier lifetime are influenced by factors such as thin film trap density, interfacial defects, and energy level mismatches, the film morphology of perovskite on the derivatives was analyzed.[48] X-ray diffraction (XRD) patterns of HTM/perovskite films, presented in **Figure 4c**, were obtained under optimal device conditions. The analysis of these patterns reveals nearly equivalent intensities across all distinctive peaks in the perovskite/HTM films, suggesting a similar crystallinity across the perovskites on different HTMs. Distinct diffraction peaks at 14.09°, 19.98°, 24.40°, 28.35°, 31.76°, 34.97°, 40.53°, and 43.14° are identified, corresponding to the (111), (120), (012), (222), (231), (030), (240), and (333) planes of the perovskite structure. Additionally, peaks observed at 12.89° and a minor one at 38.80° are assignable to the (001) and (003) lattice planes of PbI_2, respectively. Together with the on-top SEM images (**Figure 4d**; **Figure S32–S36**, Supporting Information) of the PTAA/ and HTM/perovskite bilayers, this reveals homogeneity in the perovskite morphology regardless of the HTM. Therefore, the lower PL intensity and shorter carrier lifetimes observed in previous experiments could be attributed to efficient charge separation from the perovskite layer to the derivatives. Nevertheless, it is important to note that these measurements are performed under open-circuit conditions, meaning that no actual charge carriers are extracted from the semidevice. Under these conditions, a partial polarization of the charge distribution occurs, and the reduction in PL intensity and lifetime for a bilayer compared to a bare film could be influenced by the enhancement of nonradiative losses.[49,50]

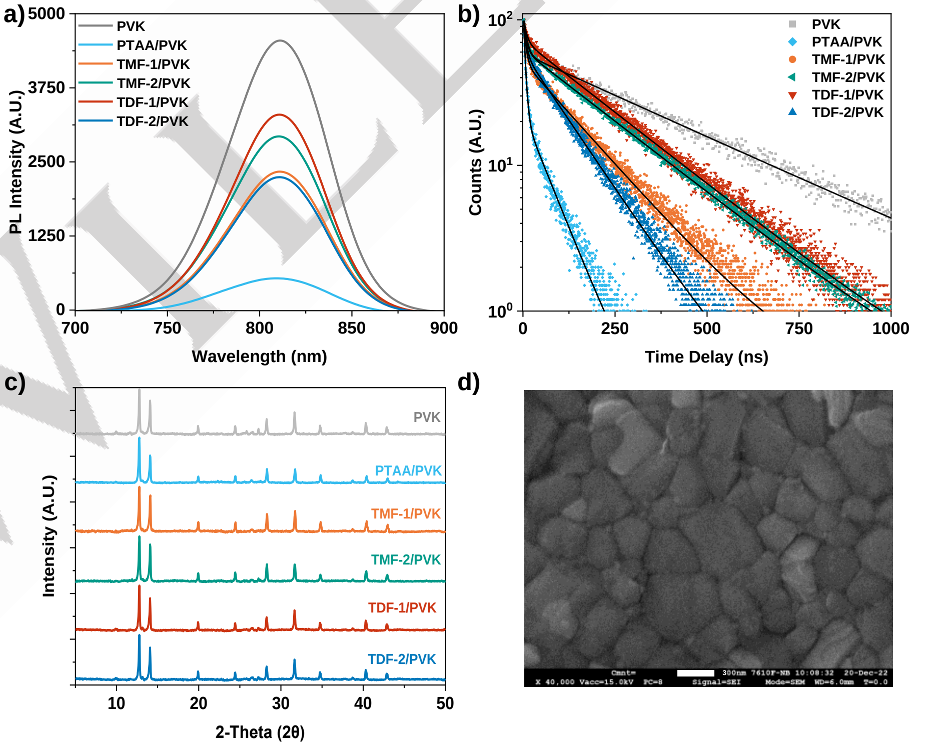

Figure 4. a) Steady-state photoluminescence spectra, b) time-resolved photoluminescence decay, The black line is the fitting result of each decay curve. c) XRD patterns of a bare $Cs_{0.17}FA_{0.83}PbI_3$ perovskite film and bilayered perovskite films with TDF and TPF series on glass substrate. d) Surface topographic SEM images of the perovskite films deposited on **TDF-2**.

The photovoltaic performance of the derivatives as hole transport materials (HTMs) was evaluated through the preparation of solution-processed perovskite solar cells (PSCs), which were compared to those using the widely recognized reference HTM, PTAA. These PSCs were fabricated in an inverted planar p-i-n configuration, following the architecture of Glass/ITO/HTM/Perovskite/$PC_{61}BM$/BCP/Ag (100 nm), with $Cs_{0.17}FA_{0.83}PbI_3$ serving as the light-harvesting material, as depicted in **Figure 5a**. The HTMs were deposited by spin-coating from a chlorobenzene solution at a concentration of 2 mg mL^{-1}, without the inclusion of additives or dopants. Further details on device fabrication are provided in the Supplementary Information. Illustrated in **Figure 5b** is the schematic of the energy levels of the various components within the PSCs. It was observed that the new HTMs displayed a favorable band alignment between their HOMO energy levels and the valence band edge of the double-cation perovskite, suggesting potential for effective hole-extraction and efficient electron-blocking. Additionally, good solubility of all derivatives in chlorobenzene was noted, which is advantageous for achieving films with desirable morphology and uniform surface coverage.

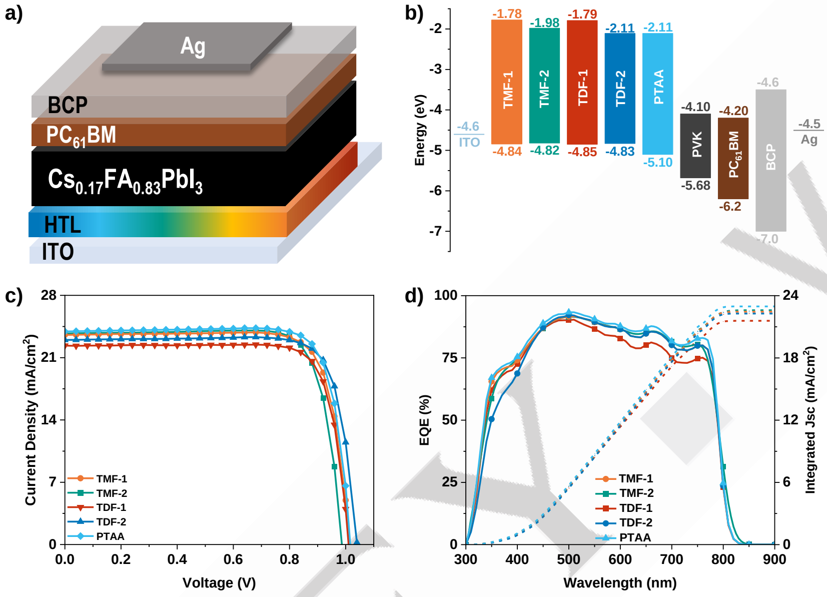

Figure 5. a) Schematic representation of planar *p-i-n* PSC device geometry and b) their corresponding energy diagrams utilized in this work. c) *J-V* characteristic curves under standard AM 1.5G illumination conditions with HTMs and d) EQE spectra of devices from (c) and the integrated J_{SC} obtained from EQE.

Under standard AM 1.5G illumination conditions, the photocurrent density-voltage (*J*–*V*) characteristics of the devices were measured at an intensity of 100 mW cm^{-2}. The resulting champion performance curves and associated data are showcased in **Figure 5c**, **Figure S37a-e** (Supporting Information), and **Table 2**. Devices based on **TDF-2** achieved an open-circuit voltage (V_{OC}) of 1.04 V, a short-circuit current (J_{SC}) of 23.00 mA cm^{-2}, and a fill factor (FF) of 0.81, culminating in the highest power conversion efficiency (PCE) of 19.38%. In contrast, devices utilizing **TMF-1** exhibited slightly lower efficiencies, with a peak PCE of 19.11%. Devices incorporating **TMF-2** reached a PCE of 18.83%, and those with **TDF-1** showed the lowest efficiency at 18.16%. It was observed that these PCE values align with the quenching sequence **TDF-2** > **TMF-1** > **TMF-2** > **TDF-1**. However, consistent with expectations from previous experiments, none of the new materials outperformed the established benchmark set by PTAA, which recorded an open-circuit voltage (V_{OC}) of 1.02 V, a short-circuit current (J_{SC}) of 23.71 mA cm^{-2}, a fill factor (FF) of 0.83, and a PCE of 20.20%. The calculated J_{SC} values integrated from the external quantum efficiencies (EQEs), displayed consistency with the experimental observations across all cases, as illustrated in **Figure 5d**. Interestingly, these performance trends correspond to the WF values observed in **Figure S19**. **TMF-1** and **TDF-2** exhibited the highest WF values, which are consistent with their superior performance. However, **TMF-1**, having a WF value closest to that of the ITO layer, might experience increased recombination with the ITO, potentially contributing to its slightly lower PCE compared to **TDF-2**.

J–*V* curves of hole-only devices of the **TMF-1-2** and **TDF-1-2** were measured in the dark, and the hole mobility (μ_h) was determined using the space-charge limited current method (SCLC) (**Figure S37f** in the Supporting Information). The hole mobility values of were determined to be 5.08×10^{-5}, 4.84×10^{-5}, 4.20×10^{-5}, and 5.52×10^{-5} $cm^2\ V^{-1}\ s^{-1}$, respectively, following the same tendency as the PL and PCE. However, the PTAA presented the highest value between the materials studied (2.60×10^{-4} $cm^2\ V^{-1}\ s^{-1}$). These results also help attribute the effects observed in the PL measurements shown in **Figure 4a-b** to the extraction capabilities of these materials.

In literature, there are three accepted methods for analyzing the hysteresis index (HI): based on the relationship between Voc and Jsc[51,52], PCE[53,54], and area under the J-V curve[55]. **Figure S38** illustrates these methods for the curves shown in **Figure S37**. Under all three methods, devices based on **TDF-2** exhibit the smallest HI, suggesting minimal ion migration and reduced charge trapping at the HTM/perovskite interface. In contrast, devices based on PTAA consistently show the highest HI across all three methods, indicating potential issues such as slower ionic movement or charge trapping.

The long-term dark stability of PSCs based on **TMF-1-2** and **TDF-1-2** was evaluated under standard AM 1.5G simulated sunlight, using non-encapsulated devices maintained in ambient air with a relative humidity of 27.5% ± 2.5% (**Figure S39**). After 264 hours,

the PTAA- and **TDF-02**-based devices retained 96% and 94% of their initial PCE, respectively. **TMF-1** followed closely, maintaining 92% of its initial performance, while **TMF-2** and **TDF-1** showed a decrease to approximately 80% of their original PCE. This degradation trend persisted over 400 hours, suggesting that significant further changes were unlikely and that the aging results could be extrapolated. Notably, the stability trend mirrors the efficiency trend, indicating that these results may be attributable to parallel factors.

Table 2. Photovoltaic parameters of the champion solar cells containing the **TMF** and **TDF** series and **PTAA**.

	Scan Direction	V_{OC} [V]	J_{SC} [mA cm^{-2}]	FF	PCE [%]
**TMF-1**	Reverse	1.01	23.57	0.80	19.11
	Forward	1.01	22.91	0.80	18.50
**TMF-2**	Reverse	0.99	23.74	0.80	18.83
	Forward	0.99	23.04	0.79	18.06
**TDF-1**	Reverse	1.01	22.33	0.80	18.16
	Forward	1.00	21.48	0.82	17.72
**TDF-2**	Reverse	1.04	23.00	0.81	19.38
	Forward	1.04	23.16	0.80	19.20
**PTAA**	Reverse	1.02	23.71	0.83	20.20
	Forward	1.03	22.74	0.82	19.30

Figure 6 presents the statistical distribution of various photovoltaic parameters across 12 planar devices for each derivative, alongside the reference PTAA. The distribution plots clearly indicate that in terms of open-circuit voltage (Voc), **TDF-2** surpasses not only the other derivatives but also the reference PTAA. Interestingly, **TMF-2** exhibits higher values for short-circuit current (Jsc), closely rivaling those of PTAA. Regarding the fill factor (FF), none of the materials exceed the performance of the benchmark PTAA, with the derivatives displaying comparable FF values among themselves. The average power conversion efficiency (PCE) values align with those observed in the individual 'champion' devices. Notably, **TDF-2** demonstrates greater consistency in its PCE data, suggesting more uniform performance across multiple devices.

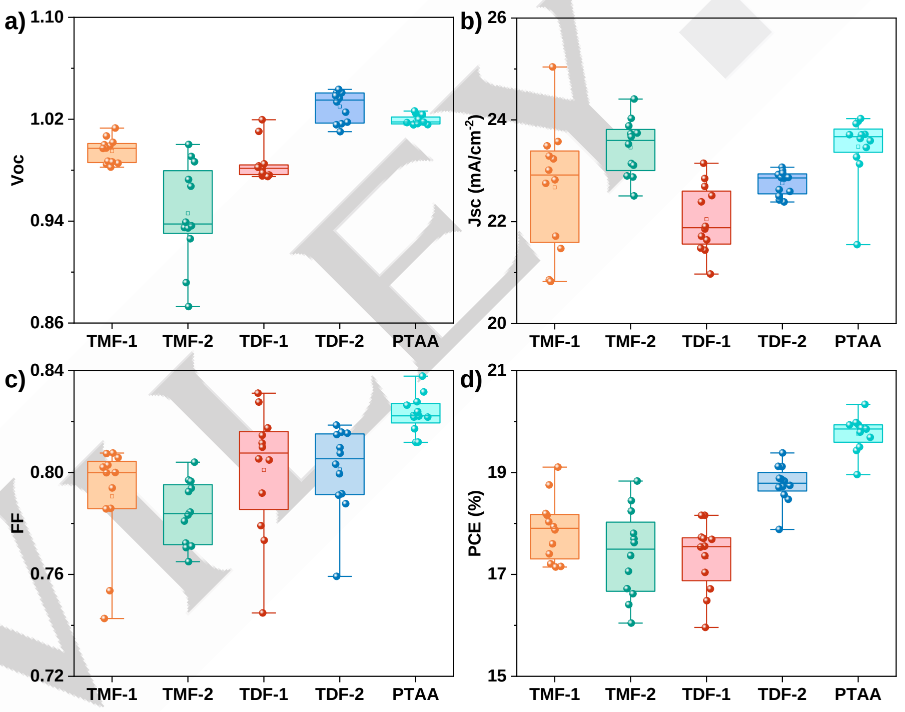

Figure 6. Statistical distribution of a) open circuit voltage (V_{OC}), b) short circuit current density (J_{SC}), c) Fill Factor (FF) and d) power conversion efficiency (PCE) for 12 devices incorporating **TMF-1-2** and **TDF-1-2** as HTMs in planar PSCs.

Conclusion

In this study, four new hole transport materials (HTMs), **TMF-1-2** and **TDF-1-2**, featuring triphenylamine and fluorene as the main core, and dimethoxydiphenylamine and dimethoxyiminodibenzyl as end-capping groups, were synthesized using Vielsmeyer-Hack, Knoevenagel, and Buchwald-Hartwig reactions. Their optical, electronic, and thermal properties were comprehensively characterized through UV-Vis spectroscopy, fluorescence, cyclic and differential pulse voltammetry, thermogravimetric analysis

(TGA), differential scanning calorimetry (DSC), and photodegradation studies, supplemented by theoretical HOMO and LUMO mapping. It was discovered that the iminodibenzyl moiety offers lower oxidation potentials, enhanced photostability, and a narrower bandgap compared to diphenylamine, making it a promising donor unit for HTMs. However, its inclusion was observed to reduce thermal resistance. Although none of the synthesized HTMs outperformed the reference material PTAA in terms of PCE (20.20 %) for $Cs_{0.17}FA_{0.83}PbI_3$-based perovskite solar cells (PSCs), all the materials demonstrated power conversion efficiency (PCE) values that were relatively close to one another. Notably, **TDF-2**, containing iminodibenzyl, exhibited the most promising performance with a PCE of 19.38 %, while **TDF-1**, with the lowest efficiency, recorded a PCE of 18.16 %. These findings highlight the potential of iminodibenzyl as a noteworthy contributor to the development of photovoltaic materials.

Supporting Information

The authors have cited additional references within the Supporting Information. [56–61]

Acknowledgements

M.C.R., J.S.R.O. and J.W. contributed equally to this work and shared first authorship. M.C.R., A.O. and B.I. thank the Universidad del Valle (C.I. 71366) and MINCIENCIAS for the Doctorados Nacionales 785/2017 Scholarship Program. J.S.R.O., J.W., V.A.M., J.A.H. and C.J.B. gratefully acknowledge the financial support from the Helmholtz Association in the framework of the innovation platform “Solar TAP”. J. W. acknowledges the financial support from the SinoGerman Postdoc Scholarship Program (CSC-DAAD). A.J.B., L.L. and C.J.B. gratefully acknowledge financial support from the German Research Foundation (DFG) (BR 4031/22-1). S.L. gratefully acknowledges the Universidad Politécnica de Madrid for providing computing resources on Magerit Supercomputer. V.A.M. thanks the Ministerio de Ciencia, tecnología e Innovación de Colombia, for the Doctorados en el Exterior 885 Scholarship Program. J.A.H. and C.J.B. gratefully acknowledge the grants “ELF-PVDesign and development of solution processed functional materials for the next generations of PV technologies” (No. 44-6521a/20/4) by the Bavarian State Government. C.J.B. gratefully acknowledges financial support through the “Aufbruch Bayern” initiative of the state of Bavaria (EnCN and “Solar Factory of the Future”), the Bavarian Initiative “Solar Technologies go Hybrid” (SolTech), and the German Research Foundation (DFG) SFB 953-No. 182849149, and GRK2495 (ITRG2495).

Keywords: Iminodibenzyl • Fluorene-based derivatives • Photostability • Hole Transport Materials • Perovskite Solar Cells

Conclusions

[1] A. Kojima, K. Teshima, Y. Shirai, T. Miyasaka, *J Am Chem Soc* **2009**, *131*, 6050–6051.
[2] W. S. Yang, J. H. Noh, N. J. Jeon, Y. C. Kim, S. Ryu, J. Seo, S. Il Seok, *Science (1979)* **2015**, *348*, 1234–1237.
[3] X. Fan, *Materials Today Sustainability* **2023**, *24*, 100603.
[4] H. Zhang, N.-G. Park, *DeCarbon* **2024**, *3*, 100025.
[5] Y. Yao, C. Cheng, C. Zhang, H. Hu, K. Wang, S. De Wolf, *Advanced Materials* **2022**, *34*, DOI 10.1002/adma.202203794.
[6] H. Chen, C. Liu, J. Xu, A. Maxwell, W. Zhou, Y. Yang, Q. Zhou, A. S. R. Bati, H. Wan, Z. Wang, L. Zeng, J. Wang, P. Serles, Y. Liu, S. Teale, Y. Liu, M. I. Saidaminov, M. Li, N. Rolston, S. Hoogland, T. Filleter, M. G. Kanatzidis, B. Chen, Z. Ning, E. H. Sargent, *Science* **2024**, *384*, 189–193.
[7] F. Ma, Y. Zhao, Z. Qu, J. You, *Acc Mater Res* **2023**, *4*, 716–725.
[8] H. Min, D. Y. Lee, J. Kim, G. Kim, K. S. Lee, J. Kim, M. J. Paik, Y. K. Kim, K. S. Kim, M. G. Kim, T. J. Shin, S. Il Seok, *Nature* **2021**, *598*, 444–450.
[9] J. Jeong, M. Kim, J. Seo, H. Lu, P. Ahlawat, A. Mishra, Y. Yang, M. A. Hope, F. T. Eickemeyer, M. Kim, Y. J. Yoon, I. W. Choi, B. P. Darwich, S. J. Choi, Y. Jo, J. H. Lee, B. Walker, S. M. Zakeeruddin, L. Emsley, U. Rothlisberger, A. Hagfeldt, D. S. Kim, M. Grätzel, J. Y. Kim, *Nature* **2021**, *592*, 381–385.
[10] C. Zhang, K. Wei, J. Hu, X. Cai, G. Du, J. Deng, Z. Luo, X. Zhang, Y. Wang, L. Yang, J. Zhang, *Materials Today* **2023**, *67*, 518–547.
[11] F. M. Rombach, S. A. Haque, T. J. Macdonald, *Energy Environ Sci* **2021**, *14*, 5161–5190.
[12] C. Aumaitre, J. Morin, *The Chemical Record* **2019**, *19*, 1142–1154.
[13] A. S. Das, A. R. Nair, A. Sreekumar, A. Sivan, *ChemistrySelect* **2022**, *7*, DOI 10.1002/slct.202201097.
[14] P. Mäkinen, F. Fasulo, M. Liu, G. K. Grandhi, D. Conelli, B. Al-Anesi, H. Ali-Löytty, K. Lahtonen, S. Toikkonen, G. P. Suranna, A. B. Muñoz-García, M. Pavone, R. Grisorio, P. Vivo, *Chemistry of Materials* **2023**, *35*, 2975–2987.
[15] R. Li, M. Liu, S. K. Matta, A. Almasri, J. Tian, H. Wang, H. P. Pasanen, S. P. Russo, P. Vivo, H. Zhang, *Solar RRL* **2023**, *7*, DOI 10.1002/solr.202300367.
[16] N. J. Jeon, H. Na, E. H. Jung, T.-Y. Yang, Y. G. Lee, G. Kim, H.-W. Shin, S. Il Seok, J. Lee, J. Seo, *Nat Energy* **2018**, *3*, 682–689.
[17] Z. Ning, H. Tian, *Chemical Communications* **2009**, 5483–5495.
[18] A. Mahmood, *Solar Energy* **2016**, *123*, 127–144.
[19] J. Wang, K. Liu, L. Ma, X. Zhan, *Chem Rev* **2016**, *116*, 14675–14725.
[20] R. Rybakiewicz, M. Zagorska, A. Pron, *Chemical Papers* **2017**, *71*, 243–268.
[21] P. Agarwala, D. Kabra, *J Mater Chem A Mater* **2017**, *5*, 1348–1373.
[22] J. S. Rocha-Ortiz, J. J. Montalvo-Acosta, Y. He, A. Insuasty, A. Hirsch, C. J. Brabec, A. Ortiz, *Dyes and Pigments* **2023**, *217*, 111445.
[23] J. S. Rocha-Ortiz, J. Wu, J. Wenzel, A. J. Bornschlegl, J. D. Perea, S. Leon, A. Barabash, A. S. Wollny, D. M. Guldi, J. Zhang, A. Insuasty, L. Lüer, A. Ortiz, A. Hirsch, C. J. Brabec, *Adv Funct Mater* **2023**, *33*, 2304262.
[24] I. Seoneray, J. Wu, J. S. Rocha-Ortiz, A. J. Bornschlegl, A. Barabash, Y. Wang, L. Lüer, J. Hauch, A. García, J. Zapata-Rivera, C. J. Brabec, A. Ortiz, *Solar RRL* **2024**, *8*, DOI 10.1002/solr.202400225.
[25] J. Wu, L. Torresi, M. Hu, P. Reiser, J. Zhang, J. S. Rocha-Ortiz, L. Wang, Z. Xie, K. Zhang, B. Park, A. Barabash, Y. Zhao, J. Luo, Y. Wang, L. Lüer, L.-L. Deng, J. A. Hauch, D. M. Guldi, M. E. Pérez-Ojeda, S. Il Seok, P. Friederich, C. J. Brabec, *Science (1979)* **2024**, *386*, 1256–1264.
[26] J. Wang, K. Liu, L. Ma, X. Zhan, *Chem Rev* **2016**, *116*, 14675–14725.
[27] A. Jegorovė, J. Xia, M. Steponaitis, M. Daskeviciene, V. Jankauskas, A. Gruodis, E. Kamarauskas, T. Malinauskas, K. Rakstys, K. A. Alamry, V. Getautis, M. K. Nazeeruddin, *Chemistry of Materials* **2023**, *35*, 5914–5923.
[28] T. Malinauskas, M. Saliba, T. Matsui, M. Daskeviciene, S. Urnikaite, P. Gratia, R. Send, H. Wonneberger, I. Bruder, M. Graetzel, V. Getautis, M. K. Nazeeruddin, *Energy Environ Sci* **2016**, *9*, 1681–1686.
[29] A. Arndt, C. W. Liria, J. K. U. Yokoyama-Yasunaka, M. T. Machini, S. R. B. Uliana, B. P. Espósito, *J Inorg Biochem* **2017**, *172*, 9–15.
[30] H. Shah, L. Pang, S. Qian, V. Sathish, *NPJ Breast Cancer* **2021**, *7*, 122.
[31] D. E. Loy, B. E. Koene, M. E. Thompson, *Adv Funct Mater* **2002**, *12*, 245–249.
[32] C. Wang, J. Li, S. Cai, Z. Ning, D. Zhao, Q. Zhang, J. H. Su, *Dyes and Pigments* **2012**, *94*, 40–48.
[33] Y. Liang, X. Xue, W. Zhang, C. Fan, Y. Li, B. Zhang, Y. Feng, *Dyes and Pigments* **2014**, *115*, 7–16.
[34] M. Caicedo-Reina, M. Pérez-Escribano, J. Urieta-Mora, I. García-Benito, J. Calbo, A. Ortiz, B. Insuasty, A. Molina-Ontoria, E. Ortí, N. Martín, *J Mater Chem C Mater* **2023**, *11*, 8223–8230.
[35] C. Wang, M. Liu, S. Rahman, H. P. Pasanen, J. Tian, J. Li, Z. Deng, H. Zhang, P. Vivo, *Nano Energy* **2022**, *101*, 107604.

WILEY VCH

[36] J. Z. Cheng, C. C. Lin, P. T. Chou, A. Chaskar, K. T. Wong, *Tetrahedron* **2011**, *67*, 734–739.
[37] Z. Liu, K. Li, Z. Hu, Z. Liu, D. Cui, *J Lumin* **2023**, *263*, 120006.
[38] W. Yan, Y. Li, S. Ye, Y. Li, H. Rao, Z. Liu, S. Wang, Z. Bian, C. Huang, *Nano Res* **2016**, *9*, 1600–1608.
[39] J. Cheng, F. Xie, Y. Liu, W. E. I. Sha, X. Li, Y. Yang, W. C. H. Choy, *J Mater Chem A Mater* **2015**, *3*, 23955–23963.
[40] A. J. Bornschlegl, P. Duchstein, J. Wu, J. S. Rocha-Ortiz, M. Caicedo-Reina, A. Ortiz, B. Insuasty, D. Zahn, L. Lüer, C. J. Brabec, *J Am Chem Soc* **2025**, DOI 10.1021/jacs.4c14824.
[41] J. Bertrandie, J. Han, C. S. P. De Castro, E. Yengel, J. Gorenflot, T. Anthopoulos, F. Laquai, A. Sharma, D. Baran, *Advanced Materials* **2022**, *34*, DOI 10.1002/adma.202202575.
[42] W. Yuan, H. Zhao, G. L. Baker, *Org Electron* **2014**, *15*, 3362–3369.
[43] T. Qin, F. Wu, L. Zhu, W. Chi, Y. Zhang, Z. Yang, J. Zhao, Z. Chi, *Org Electron* **2022**, *100*, 106325.
[44] P. Dhingra, P. Singh, P. J. S. Rana, A. Garg, P. Kar, *Energy Technology* **2016**, *4*, 891–938.
[45] J. Siekmann, A. Kulkarni, S. Akel, B. Klingebiel, M. Saliba, U. Rau, T. Kirchartz, *Adv Energy Mater* **2023**, *13*, DOI 10.1002/aenm.202300448.
[46] L. Krückemeier, B. Krogmeier, Z. Liu, U. Rau, T. Kirchartz, *Adv Energy Mater* **2021**, *11*, DOI 10.1002/aenm.202003489.
[47] T. Kirchartz, J. A. Márquez, M. Stolterfoht, T. Unold, *Adv Energy Mater* **2020**, *10*, DOI 10.1002/aenm.201904134.
[48] H. Tsai, C. Liu, E. Kinigstein, M. Li, S. Tretiak, M. Cotlet, X. Ma, X. Zhang, W. Nie, *Advanced Science* **2020**, *7*, 1–8.
[49] L. Krückemeier, B. Krogmeier, Z. Liu, U. Rau, T. Kirchartz, *Adv Energy Mater* **2021**, *11*, DOI 10.1002/aenm.202003489.
[50] V. Campanari, F. Martelli, A. Agresti, S. Pescetelli, N. Y. Nia, F. Di Giacomo, D. Catone, P. O'Keeffe, S. Turchini, B. Yang, J. Suo, A. Hagfeldt, A. Di Carlo, *Solar RRL* **2022**, *6*, DOI 10.1002/solr.202200049.
[51] F. Yang, G. Kapil, P. Zhang, Z. Hu, M. A. Kamarudin, T. Ma, S. Hayase, *ACS Appl Mater Interfaces* **2018**, *10*, 16482–16489.
[52] K. Wang, Y. Shi, B. Li, L. Zhao, W. Wang, X. Wang, X. Bai, S. Wang, C. Hao, T. Ma, *Advanced Materials* **2016**, *28*, 1891–1897.
[53] Q. Wali, M. Aamir, A. Ullah, F. J. Iftikhar, M. E. Khan, J. Akhtar, S. Yang, *Chemical Record* **2022**, *22*, DOI 10.1002/tcr.202100150.
[54] S. N. Habisreutinger, N. K. Noel, H. J. Snaith, *ACS Energy Lett* **2018**, *3*, 2472–2476.
[55] G. A. Nemnes, C. Besleaga, A. G. Tomulescu, A. Palici, L. Pintilie, A. Manolescu, I. Pintilie, *Solar Energy* **2018**, *173*, 976–983.
[56] C. A. Echeverry, A. Insuasty, M. Á. Herranz, A. Ortíz, R. Cotta, V. Dhas, L. Echegoyen, B. Insuasty, N. Martín, *Dyes and Pigments* **2014**, *107*, 9–14.
[57] R. Pashazadeh, P. Pander, A. Bucinskas, P. J. Skabara, F. B. Dias, J. V. Grazulevicius, *Chemical Communications* **2018**, *54*, 13857–13860.
[58] T. Tromholt, M. Manceau, M. Helgesen, J. E. Carlé, F. C. Krebs, *Solar Energy Materials and Solar Cells* **2011**, *95*, 1308–1314.
[59] J. A. Röhr, D. Moia, S. A. Haque, T. Kirchartz, J. Nelson, *Journal of Physics Condensed Matter* **2018**, *30*, DOI 10.1088/1361-648X/aaabad.
[60] V. M. Le Corre, E. A. Duijnstee, O. El Tambouli, J. M. Ball, H. J. Snaith, J. Lim, L. J. A. Koster, *ACS Energy Lett* **2021**, *6*, 1087–1094.
[61] J. Wang, H. Zhang, B. Wu, Z. Wang, Z. Sun, S. Xue, Y. Wu, A. Hagfeldt, M. Liang, *Angewandte Chemie - International Edition* **2019**, *58*, 15721–15725.

WILEY ◆ VCH

Entry for the Table of Contents

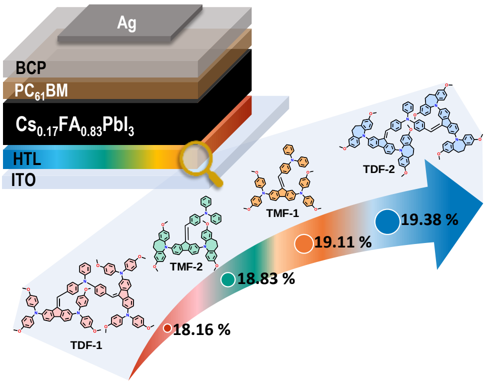

This study introduces novel fluorene-based hole transport materials (HTMs) for perovskite solar cells (PSCs), integrating iminodibenzyl and diphenylamine moieties. These materials exhibit remarkable photostability, optical, and thermal properties. Among them, TDF-2 achieved a power conversion efficiency (PCE) of 19.38%. The findings highlight iminodibenzyl's potential to address stability and cost challenges, paving the way for next-generation HTMs in PSCs.

WILEY-VCH

Supporting Information

Comparative Study of Iminodibenzyl and Diphenylamine Derivatives as Hole Transport Materials in Inverted Perovskite Solar Cells

Mauricio Caicedo-Reina[a]†, *Juan S. Rocha-Ortiz*[b,c]*†, *Jianchang Wu*[b,c]†, *Andreas J. Bornschlegl*[c], *Salvador Leon*[d], *Anastasia Barabash*[c], *Jose Dario Perea*[e], *Yunuo Wang*[c], *Vanessa Arango-Marín*[b,c], *Alejandro Ortiz*[a], *Larry Lüer*[c], *Jens Hauch*[b], *Braulio Insuasty*[a], *Christoph J. Brabec*[b,c]*.

[a]Universidad del Valle, Departamento de Química, Grupo de Investigación de Compuestos Heterocíclicos, Calle 13 #100-00, 760032 Cali, Colombia.

[b]Helmholtz-Institute Erlangen-Nürnberg (HI-ERN), Immerwahrstraße 2, 91058 Erlangen, Germany.

[c]Friedrich-Alexander-Universität Erlangen-Nürnberg, Department of Materials Science and Engineering, Institute of Materials for Electronics and Energy Technology (i-MEET), Martensstraße 7, 91058 Erlangen, Germany

[d]Universidad Politécnica de Madrid, Departamento de Ingeniería Química, ETSIIM, José Gutiérrez Abascal 2, 28006 Madrid, Spain.

[e]Universidad Icesi, Departamento de Ingeniería Bioquímica, Calle 18 No. 122-135, 760031 Cali, Colombia.

†These authors contributed equally to this work.

*Corresponding authors: juan.sebastian.rocha@fau.de; christoph.brabec@fau.de

WILEY-VCH

1. Materials and Methods

All chemicals were purchased from Sigma Aldrich (SA), VWR, BLDpharm or TCI and were used without any further purification. Solvents were distilled before usage. Dichloromethane was neutralized with K_2CO_3 before distillation. Thin-layer chromatography (TLC) was performed on Merck silica gel 60 F254, detected by UV light (254 or 366 nm) or by developing. Column chromatography was performed on MachereyNagel silica gel 60 M (deactivated, 230−400 mesh). MALDI-ToF (nitrogen UV laser, 337 nm) mass spectra were obtained by using a Bruker ultrafleXreme spectrometer with 2,5-dihydroxybenzoic acid (DHB) or (*E*)-2-(3-(4-(tert-butyl)phenyl)-2-methylallylidene)- malononitrile (dctb) used as matrices. ESI/APPI-ToF mass spectrometry was carried out on a Bruker maXis 4G UHR TOF MS/MS spectrometer or a Bruker micrOTOF II focus TOF MS spectrometer. Elemental analyses were performed using a UNICUBE elemental analyser and the values are within ±0.4% of the theoretical values. Materials **TMF-1**, **TMF-2**, **TDF-1** and **TDF-2** were dissolved in chloroform to acquire solutions of a concentration of 3 mg/mL. The solutions were stirred overnight at room temperature and 250 rpm. Quartz substrates were cleaned sequentially in detergent (Hellmanex), DI water, acetone, and isopropanol in an ultrasonic bath for 10 minutes each. Static spincoats of 50 uL solution were performed with a spincoat robot (Sciprios, SpinBot One) at 5000 acceleration and 600 rpm for 30s. The so-created thin films were subsequently characterized and degraded. The samples were placed inside a sealed chamber with a quartz window and degraded under a 254 nm UVC lamp (Philips, TUV PL-L 35W/4P HO 1CT/25) at an irradiance of 22.3 mW/cm^2 for 50 hours. The exact UVC irradiance on each sample was recorded with a UVC photodetector (sglux TOCON_C8) to allow for an accurate calculation of the number of absorbed UVC photons (see Figure S15). A constant nitrogen flow of 2 lpm (< 1 ppm of oxygen) through the chamber reduces photooxidation effects to a minimum.

A water-cooled (18°C) aluminum board under the chamber and good thermal contact guarantee low temperatures in the samples. UV-Vis spectra were recorded between 200 nm and 800 nm with an Ulbricht-sphere (Perkin Elmer, Lambda 950). PL spectra were recorded by exciting the films at 375 nm with a diode laser (Vortran, Stradus 375-60) and detecting the PL emission with the CCD camera (Horiba, Syncerity) of the spectrometer (Horiba, iHR 320). Electrochemical measurements were conducted in a classical three-electrode cell from Deutsche Metrohm GmbH & Co. KG, which was connected to Metrohm Autolab PGSTAT 101, controlled by NOVA 2.1 software, running on a personal computer. As a working

WILEY-VCH

electrode, a motionless gold electrode tip (0.03 cm^2) was used combined with a platinum sheet (1.0 cm^2) that served as a counter electrode. All potentials are presented relative to an Ag/AgCl (2 M lithium chloride in ethanol) reference electrode with a potential of 0.164 V *vs* SHE at 21 ± 1 °C. Spectra were recorded in anhydrous *o*-dichlorobenzene (HPLC grade) at 21 ± 1 °C with 0.1 M n-Bu_4NPF_6 as a supporting electrolyte. For cyclic voltammetry, two different scan rates of ν = 50 and 100 mVs^{-1} were chosen, whereas differential pulse voltammetry was conducted with a scan rate of ν = 10 mVs^{-1}. *o*-dichlorobenzene was deoxygenated with nitrogen (2 min/mL) before each measurement. The nitrogen atmosphere was maintained during all measurements. Thermogravimetric analysis (TGA) was performed using a Perkin Elmer Pyris 1 TGA Thermogravimetric Analyzer with a ramp of 10 °C min^{-1} under N_2 from 100 to 1000 °C. Differential scanning calorimetry (DSC) was run on a Toledo Mettler DSC822e Differential Scanning Calorimeter, heating at 20 °C min^{-1} of scanning rate. The J–V curves of solar cells were measured by Oriel Sol 1A Solar simulator under AM 1.5 G irradiation (100 mW cm^{-2}) at ambient conditions, calibrated with a standard Si solar cell and obtained by the linear sweep voltammetry (LSV) method using a Keithley 2400 source-measure unit. The light intensity was calibrated with a crystalline Si cell. The J-V characteristics were performed from -0.2 to 1.4 V (forward scan) and from 1.4 to -0.2 V (reverse scan) at a scan rate of 20 ms/step, scan step of 40 mV and delay time of 100 ms. The EQE spectra were recorded on a commercial EQE measurement system (Taiwan, Enlitech, QE-R) under ambient conditions and the light intensity at each wavelength was calibrated with a standard single-crystal Si photovoltaic cell. TRPL and STPL curves were recorded using a Fluotime 300 spectrometer. The samples were excited with the wavelength of 402 nm by PDL 820 picosecond diode laser with an average incident power of 4 μW at a frequency of 20000 kHz. A cutting-off filter (435 nm) was adopted to block the excitation laser. EasyTau software was used to control the measurement. An FEI Helios NanoLab 660 apparatus was used to acquire Scanning electron microscopy (SEM) images. The SEM micrographs of samples were recorded at an acceleration voltage of 5 kV. X-ray powder diffraction (XRD) measurements were performed by classical ex-situ Bragg-Brentano geometry using a PANalytical X'pert powder diffractometer with filtered Cu-Kα radiation and an X'Celerator solid-state stripe detector operated at 40 kV and 30 mA under ambient condition, with a step size of 0.04, from 3° to 60°. All samples were deposited on Glass/ITO substrates and measured under identical conditions. The contact angle measurements were carried out in an OCA 20 device from DataPhysics.

WILEY-VCH

2. Synthetic details and characterization.

Compounds 2, 3[1] and 8[2] were prepared according to previously reported synthetic procedures and showed identical spectroscopic properties to those reported therein.

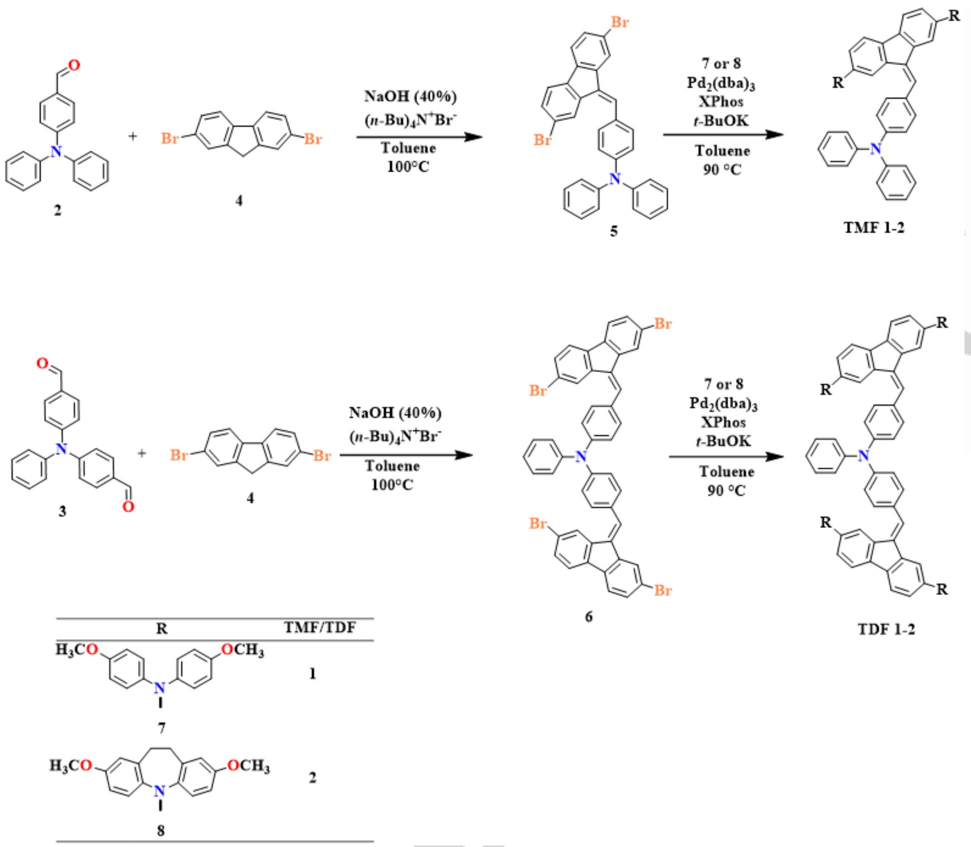

Scheme S1. Synthetic methodology for the preparation of **TMF 1-2** and **TDF 1-2.**

Compound 5. Aqueous 40 % NaOH solution (20 mL) was added to a solution of compound **2** (362 mg, 1.32 mmol), 2,7-dibromofluorene (642 mg, 1.98 mmol) and tetrabutylammonium bromide (155 mg, 0.48 mmol) in toluene (20 mL). Then, the resultant mixture was heated to 100 °C for 2 hours. After cooling to room temperature, water was added and the product was extracted in dicloromethane. The organic phase was dried over anhydrous sodium sulfate and the solvent was removed under reduced pressure to afford a yellow solid (500 mg, 65 %). ^{1}H NMR (400 MHz, $CDCl_3$, 25°C, TMS, ppm): δ= 7.88 (d, J = 8.0 Hz, 2H), 7.63 (s, 1H), 7.53 (d, J = 8.0 Hz, 2H), 7.48-7.42 (m, 4H), 7.34-7.30 (m, 4H), 7.21 (d, J = 8.0 Hz, 4H), 7.15 (d, J = 8.0 Hz, 2H), 7.08-7.11 (m, 2H); ^{13}C NMR (100 MHz, $CDCl_3$, 25°C, TMS, ppm): δ= 148.8, 147.3, 141.5, 139.0, 138.3, 136.8, 133.4, 131.2, 130.9, 130.7, 130.4, 129.6, 128.9, 127.4, 125.2, 123.8, 123.6, 122.4, 121.3, 121.1, 121.0, 120.8; MS (EI) calcd for $C_{32}H_{21}Br_2N$ [M^+] 579.34;

found 579.10. Elemental analysis calcd for $C_{32}H_{21}Br_2N$: C 66.34, H 3.65, N 2.42, found: C 66.61, H 3.47, N 2.53,

Compound 6. Aqueous 40 % NaOH solution (20 mL) was added to a solution of compound **3** (800 mg, 2.65 mmol), 2,7-dibromofluorene (2.60 g, 7.95 mmol) and tetrabutylammonium bromide (308 mg, 0.95 mmol) in toluene (20 mL). Then, the resultant mixture was heated to 100 °C for 2 hours. After cooling to room temperature, water was added and the product was extracted in dichloromethane. The organic phase was dried over anhydrous sodium sulfate and the solvent was removed under reduced pressure to afford a yellow solid (1.10 g, 45 %). ^{1}H NMR (400 MHz, $CDCl_3$, 25°C, TMS, ppm): δ= 7.87 (dd, J_1 = 4.0 Hz, J_2 = 8.0 Hz, 4H), 7.64 (s, 2H), 7.54-741 (m, 12H), 7.38 (d, J = 8.0 Hz, 2H), 7.33-7.27 (m, 6H), 7.17 (t, J = 8.0 Hz, 2H); ^{13}C NMR (100 MHz, $CDCl_3$, 25°C, TMS, ppm): δ= 148.0, 146.8, 141.3, 139.0, 138.2, 136.9, 133.9, 131.3, 131.0, 130.8, 130.1, 130.0, 129.9, 127.5, 125.7, 124.5, 123.6, 121.3, 121.1, 121.0, 120.9; MS (EI) calcd for $C_{46}H_{27}Br_4N$ [M$^+$] 913.35; found 913.05. Elemental analysis calcd for $C_{46}H_{27}Br_4N$: C 60.49, H 2.98, N 1.53, found: C 60.64, H 3.10, N 1.41

TMF 1. A solution of compound **5** (100 mg, 0.17 mmol), 4,4′-dimethoxydiphenylamine **(7)** (123 mg, 0.41 mmol), $Pd_2(dba)_3$ (5 % mol), XPhos (10 % mol), *t*-BuOK (76 mg, 0.68 mmol) in dry toluene was degassed for 30 minutes. The reaction was heated to 80 °C for 18 hours under argon atmosphere. Then, the mixture was cooled to room temperature and filtered through silica. The crude product was purified by column chromatography on silica (dichloromethane/hexane, 3:2) to afford **TMF 1** as a red wine solid (116 mg, yield: 78 %). ^{1}H NMR (400 MHz, [D$_8$]THF, 25°C, TMS, ppm): δ= 7.58 (d, J = 4.0 Hz, 1H), 7.43 (dd, J_1 = 1.0 Hz, J_2 = 8.0 Hz, 2H), 7.38 (d, J = 4.0 Hz, 1H), 7.33 (s, 1H), 7.26-7.22 (m, 6H), 7.02-6.98 (m, 10H), 6.96-6.92 (m, 4H), 6.91-6.87 (m, 2H), 6.84-6.79 (m, 5H), 6.77-6.72 (m, 5H), 3.74 (s, 6H), 3.59 (s, 6H); ^{13}C NMR (100 MHz, [D$_8$]THF, 25°C, TMS, ppm): δ= 156.8 (C), 156.7 (C), 148.7 (C), 148.5 (C), 148.4 (C), 148.2 (C), 142.6 (C), 142.4 (C), 142.1 (C), 138.5 (C), 136.0 (C), 135.9 (C), 134.1 (C), 131.7 (C), 131.3 (CH), 130.1 (CH), 127.7 (CH), 126.6 (CH), 126.5 (CH), 125.2 (CH), 124.8 (CH), 123.7 (CH), 123.5 (CH), 123.1 (CH), 120.0 (CH), 119.9 (CH), 115.4 (CH), 115.0 (CH), 55.6 (CH_3); FTIR (neat, cm^{-1}): 2929, 2831, 1587, 1600, 1498, 1456, 1439, 1319, 1271, 1234, 1174, 1105, 10.33, 821, 752, 696; HRMS calcd for $C_{60}H_{49}N_3O_4$ [M$^+$] 876.0690; found 875.4861.

TMF 2. A solution of compound **5** (100 mg, 0.17 mmol), 2,8-dimethoxy-10,11-dihydro-5H-dibenzo[b,f]azepine (**8**) (105 mg, 0.41 mmol), $Pd_2(dba)_3$ (5 % mol), XPhos (10 % mol), *t*-BuOK

(76 mg, 0.68 mmol) in dry toluene was degassed for 30 minutes. The reaction was heated to 80 °C for 18 hours under argon atmosphere. Then, the mixture was cooled to room temperature and filtered through silica. The crude product was purified by column chromatography on silica (dichloromethane/hexane, 7:3) to afford **TMF 2** as an orange solid (116 mg, yield: 73 %). ^{1}H NMR (400 MHz, $CDCl_3$, 25°C, TMS, ppm): δ= 7.35-7.26 (m, 9H), 7.21 (d, J = 4H), 7.14-7.10 (m, 5H), 7.07-7.04 (m, 2H), 6.89 (d, J = 8.0 Hz, 2H), 6.84 (s, 1H), 6.81-6.79 (m, 4H), 6.69-6.67 (m, 4H), 6.54-6.46 (br, 2H), 3.81 (s, 6H), 3.65 (s, 6H), 2.96 (s, 4H), 2.90 (s, 4H); ^{13}C NMR (100 MHz, $CDCl_3$, 25°C, TMS, ppm): δ= 158.3 (C), 158.1 (C), 148.7 (C), 148.5 (C), 147.7 (C), 147.1 (C), 141.0 (C), 137.3 (C), 131.3 (CH), 131.1 (CH), 130.6 (C), 130.4 (CH), 129.4 (CH), 125.9 (CH), 124.5 (CH), 123.7 (CH), 123.1 (CH), 118.6 (CH), 115.7 (CH), 115.3 (CH), 113.8 (CH), 112.9 (CH), 112.6 (CH), 109.4 (CH), 104.3 (CH), 55.5 (CH_3), 55.4 (CH_3), 31.2 (CH_2), 31.2 (CH_2); FTIR (neat, cm^{-1}): 2951, 2930, 2904, 2831, 1598, 1589, 1490, 1460, 1425, 1325, 1267, 1224, 1147, 1037, 873, 806, 754, 692; HRMS calcd for $C_{64}H_{53}N_3O_4$ [M$^+$] 928.1450; found 927.2820.

TDF 1. A solution of compound **6** (100 mg, 0.11 mmol), 4,4′-dimethoxydiphenylamine **(7)** (122 mg, 0.53 mmol), $Pd_2(dba)_3$ (10 % mol), XPhos (20 % mol), *t*-BuOK (100 mg, 0.88 mmol) in dry toluene was degassed for 30 minutes. The reaction was heated to 80 °C for 18 hours under argon atmosphere. Then, the mixture was cooled to room temperature and filtered through silica. The crude product was purified by column chromatography on silica (dichloromethane/hexane, 4:1) to afford **TDF 1** as a black solid (90 mg, yield: 55 %). ^{1}H NMR (400 MHz, $CDCl_3$, 25°C, TMS, ppm): δ= 7.57 (s, 2H), 7.40 (d, J = 8.0 Hz, 4H), 7.33 (s, 2H), 7.28-7.22 (m, 9H), 7.06 (d, J = 8.0 Hz, 9H), 6.94 (d, J = 8.0 Hz, 13H), 6.82 (d, J = 8.0 Hz, 8H), 6.72-6.68 (m, 12H), 3.79 (s, 12H), 3.58 (s, 12H); ^{13}C NMR (100 MHz, $CDCl_3$, 25°C, TMS, ppm): δ= 155.5, 155.3, 147.1, 147.1, 141.7, 141.6, 141.1, 137.6, 135.2, 135.0, 133.0, 130.6, 129.5, 125.9, 125.5, 124.8, 123.8, 119.7. 119.5, 114.7, 114.7, 114.2, 55.6, 55.5; FTIR (neat, cm^{-1}): 2947, 2900, 2831, 1587, 1500, 1456, 1498, 1319, 1269, 1232, 1174, 1031, 819; HRMS calcd for $C_{102}H_{83}N_5O_8$ [M$^+$] 1506.8130; found 1506.4602.

TDF 2. A solution of compound **6** (100 mg, 0.11 mmol), 2,8-dimethoxy-10,11-dihydro-5H-dibenzo[b,f]azepine **(8)** (122 mg, 0.53 mmol), $Pd_2(dba)_3$ (10 % mol), XPhos (20 % mol), *t*-BuOK (100 mg, 0.88 mmol) in dry toluene was degassed for 30 minutes. The reaction was heated to 80 °C for 18 hours under argon atmosphere. Then, the mixture was cooled to room temperature and filtered through silica. The crude product was purified by column

WILEY-VCH

chromatography on silica (dichloromethane/hexane, 4:1) to afford **TDF 2** as a brown solid (95 mg, yield: 54 %). ^{1}H NMR (400 MHz, $CDCl_3$, 25°C, TMS, ppm): δ= 7.38-7.34 (m, 12H), 7.26-7.09 (m, 14H), 6.93 (d, J = 8.0 Hz, 4H), 6.86 (s, 2H), 6.80 (d, J = 8.0 Hz, 8H), 6.68 (d, J = 8.0 Hz, 8H), 6.55-6.38 (br, 3H), 3.80 (s, 12H), 3.64 (s, 12H), 2.96 (s, 8H), 2.90 (s, 8H); ^{13}C NMR (100 MHz, $CDCl_3$, 25°C, TMS, ppm): δ= 158.3, 158.2, 147.4, 146.7, 137.6, 137.3, 130.9, 130.5, 129.5, 129.5, 124.8, 123.8, 115.7, 115.3, 113.0, 112.6, 55.5, 55.4, 31.2, 31.2; FTIR (neat, cm^{-1}): 2929, 2905, 2831, 1602, 1494, 1454, 1317, 1265, 1220, 1147, 1097, 1039, 871, 804; HRMS calcd for $C_{110}H_{91}N_5O_8$ [M^+] 1610.9650; found 1610.7005.

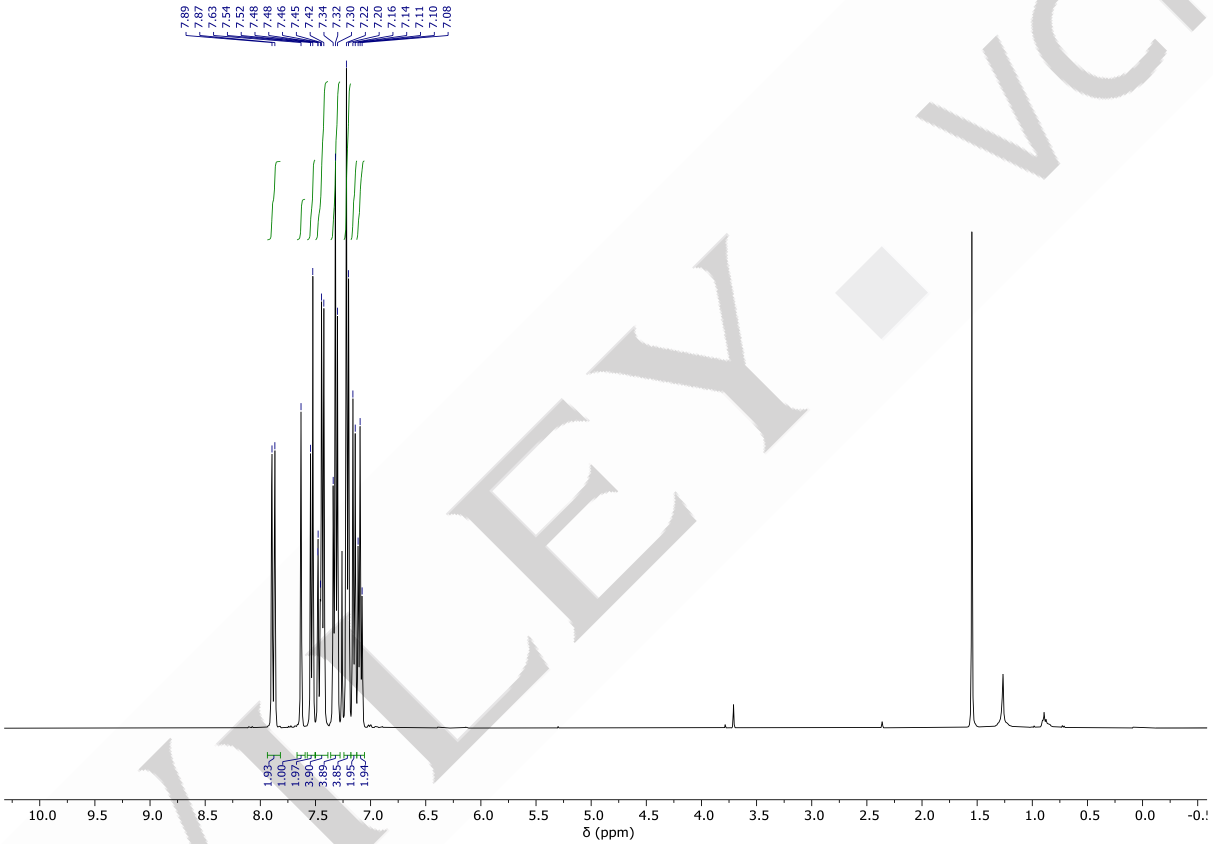

Figure S1. ^{1}H NMR (400 MHz, $CDCl_3$, 298 K) of compound **5.**

WILEY • VCH

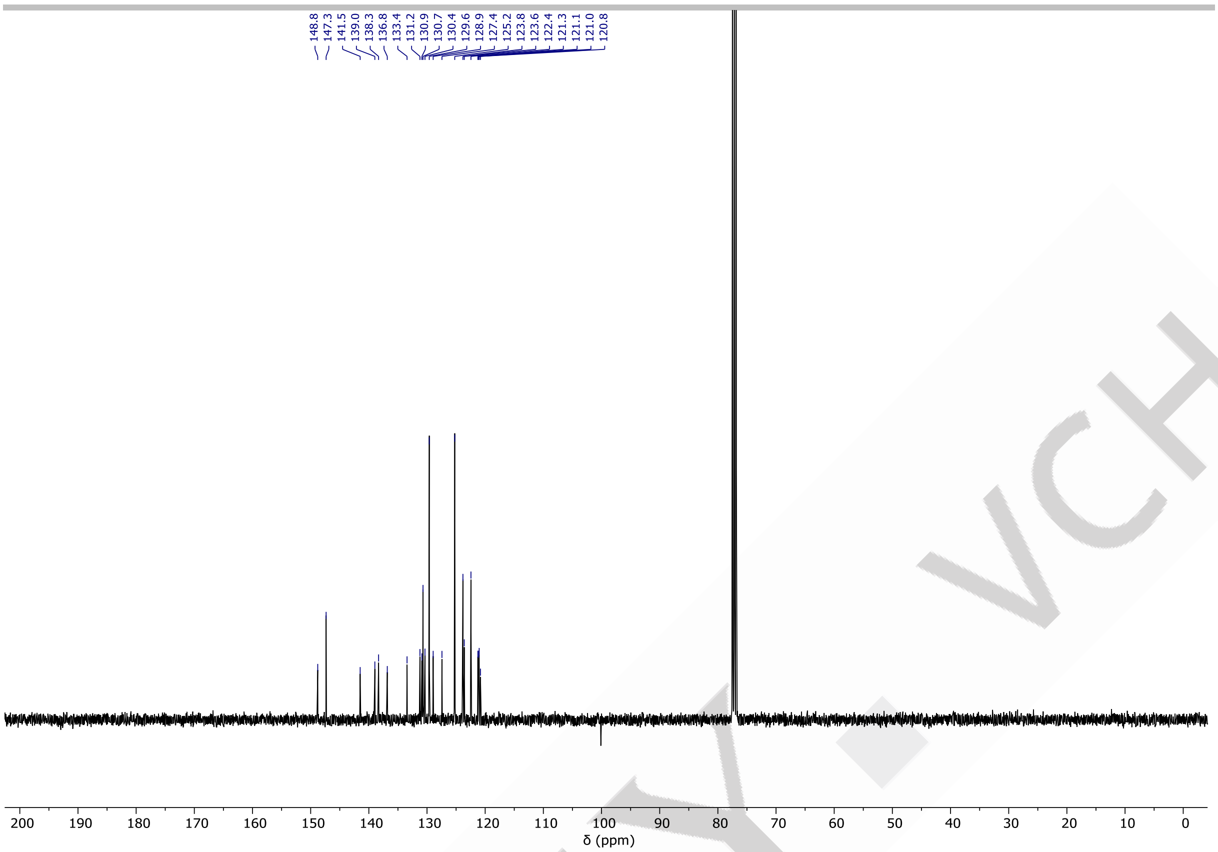

Figure S2. ^{13}C NMR (100 MHz, $CDCl_3$, 298 K) of compound **5.**

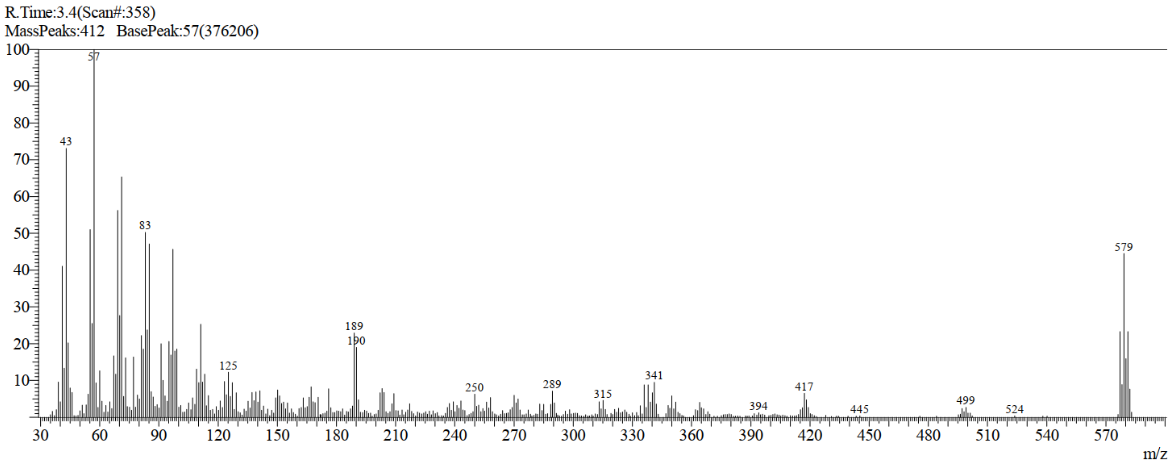

Figure S3. EI mass spectrum of compound **5.**

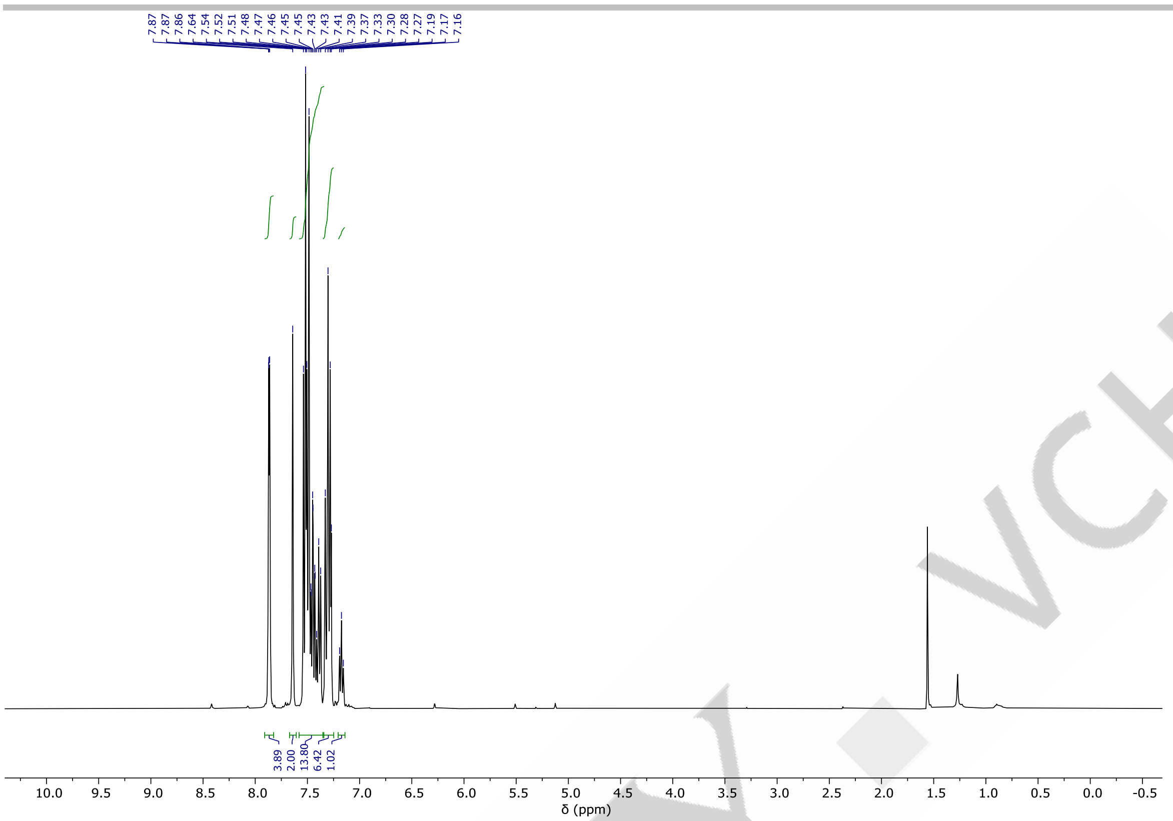

Figure S4. ^{1}H NMR (400 MHz, $CDCl_3$, 298 K) of compound **6.**

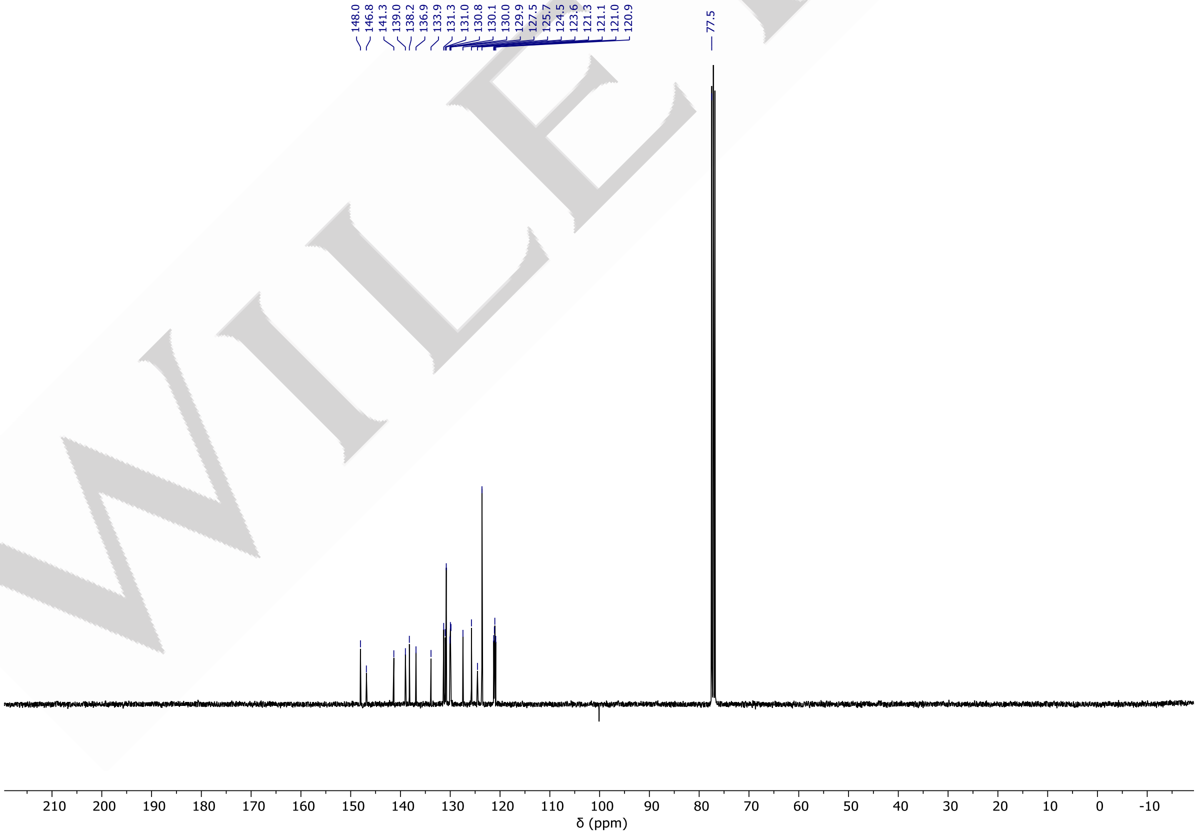

Figure S5. ^{13}C NMR (100 MHz, $CDCl_3$, 298 K) of compound **5.**

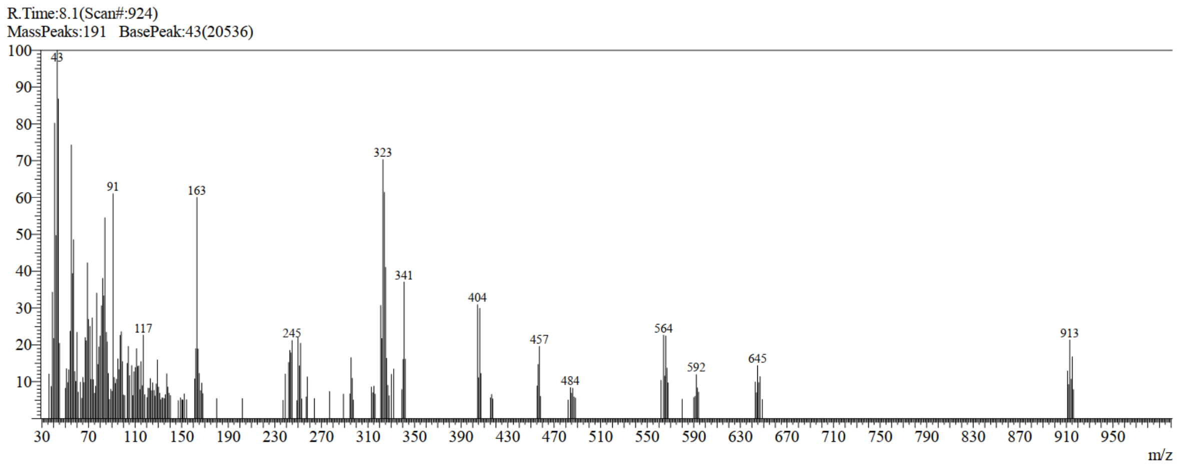

Figure S6. EI mass spectrum of compound **5.**

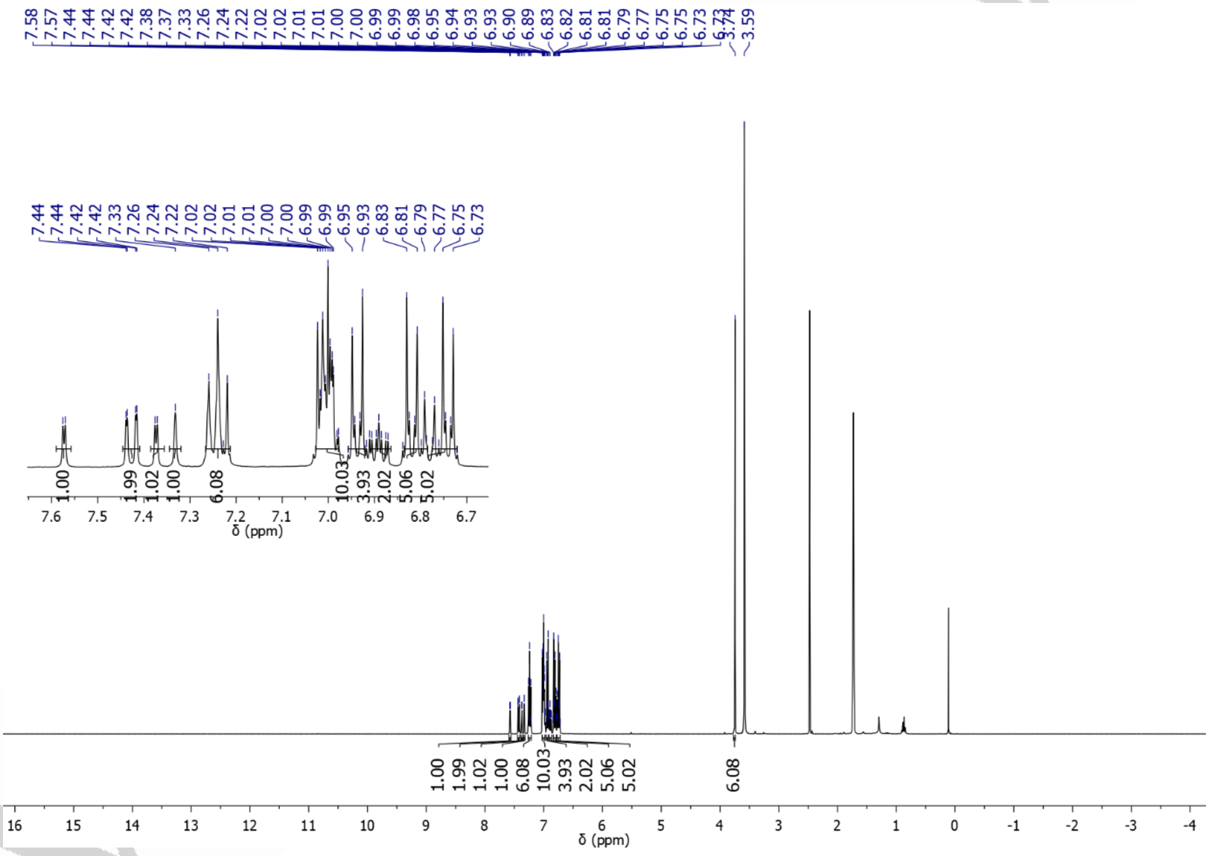

Figure S7. ^{1}H NMR (400 MHz, THF-d_8, 298 K) of compound **TMF 1.**

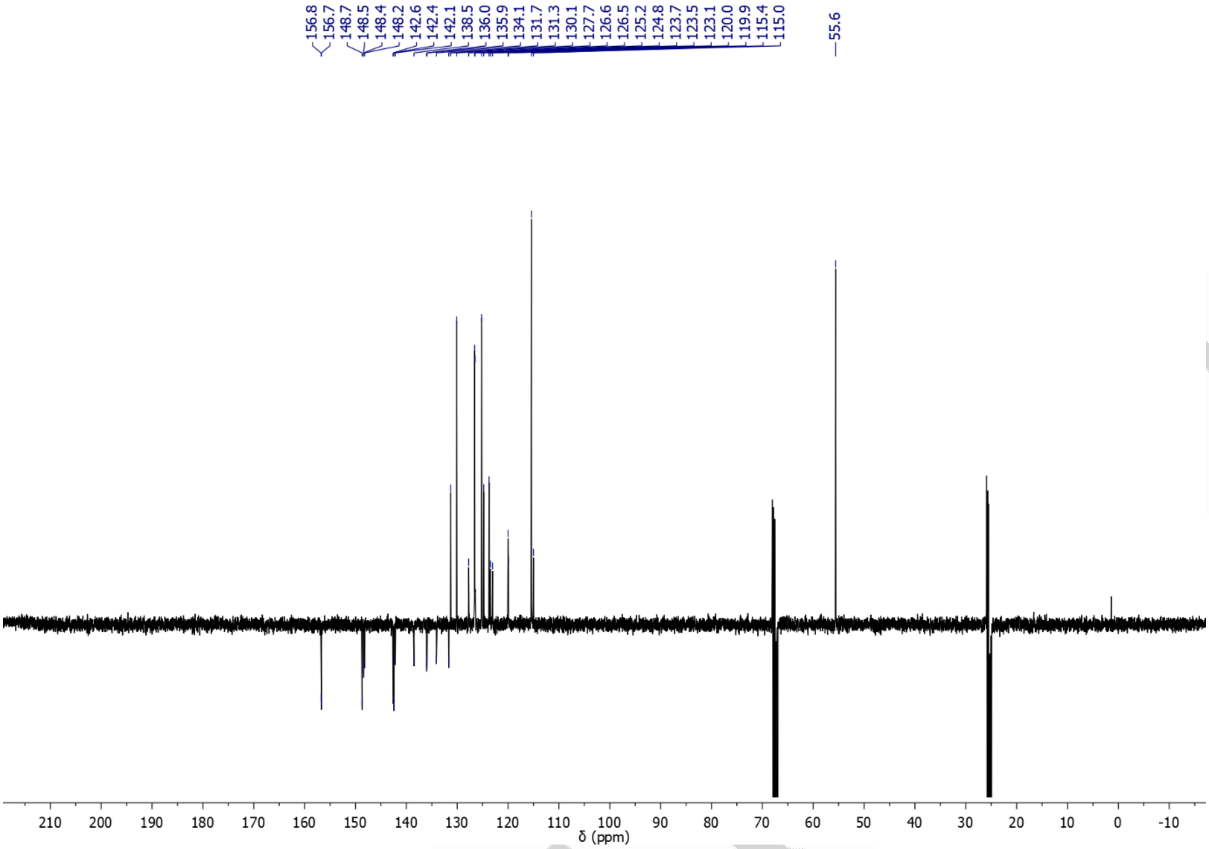

Figure S8. DEPTQ 135 (100 MHz, THF-d_8, 298 K) of compound **TMF 1.**

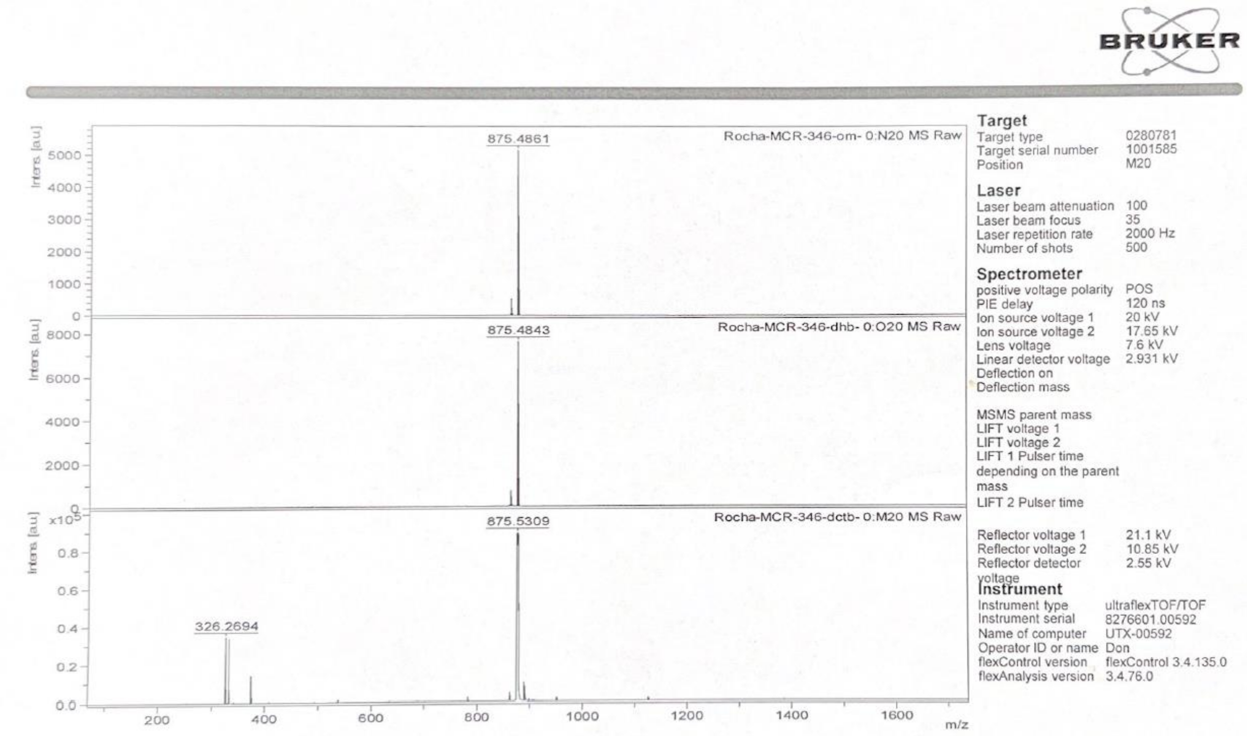

Figure S9. HR-MALDI-TOF mass spectrum of compound **TMF 1.**

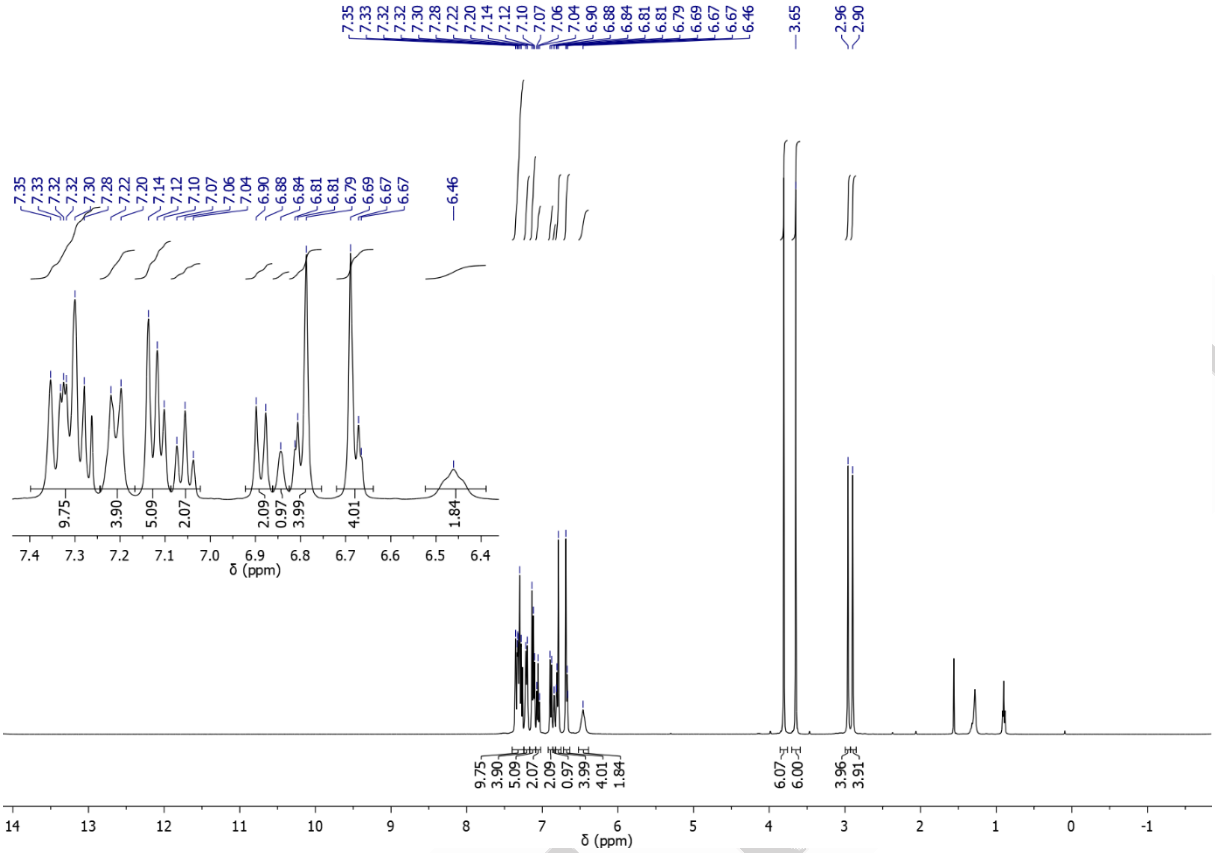

Figure S10. ^{1}H NMR (400 MHz, $CDCl_3$, 298 K) of compound **TMF 2.**

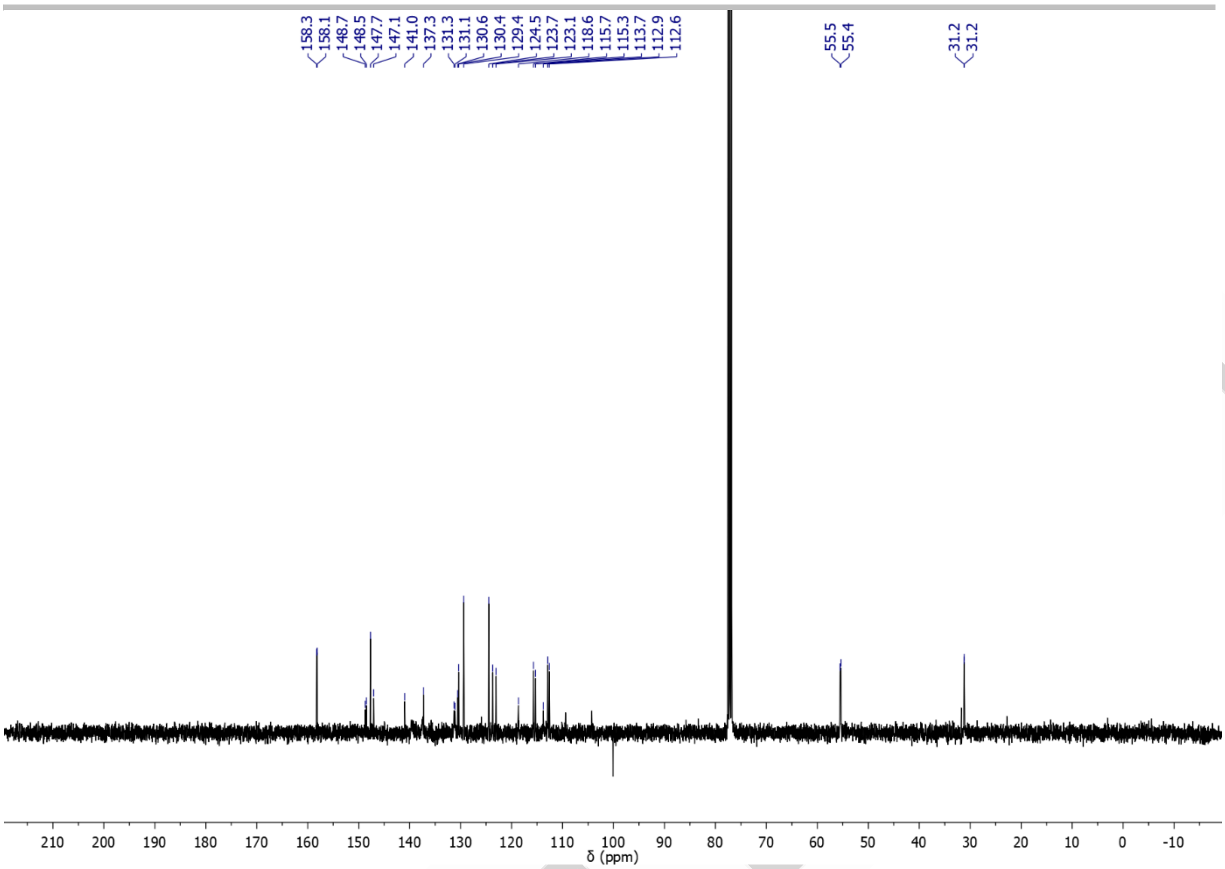

Figure S11. ^{13}C NMR (100 MHz, $CDCl_3$, 298 K) of compound **TMF 2.**

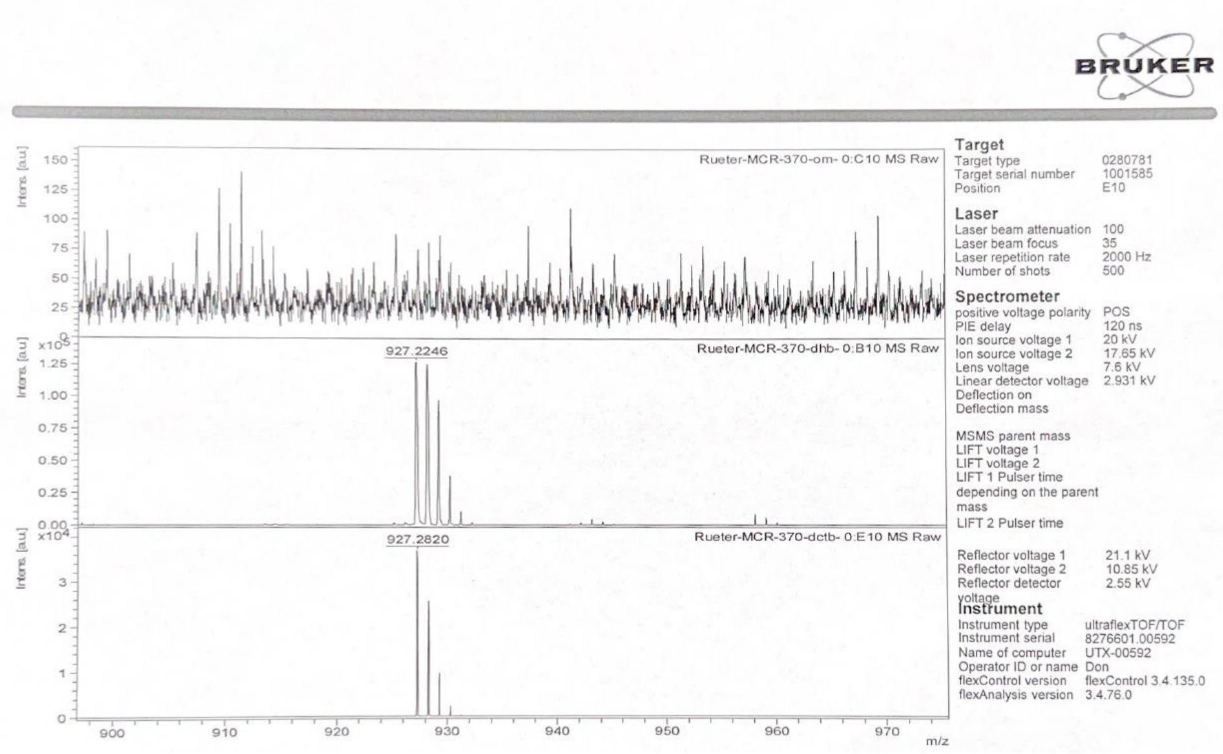

Figure S12. HR-MALDI-TOF mass spectrum of compound **TMF 2.**

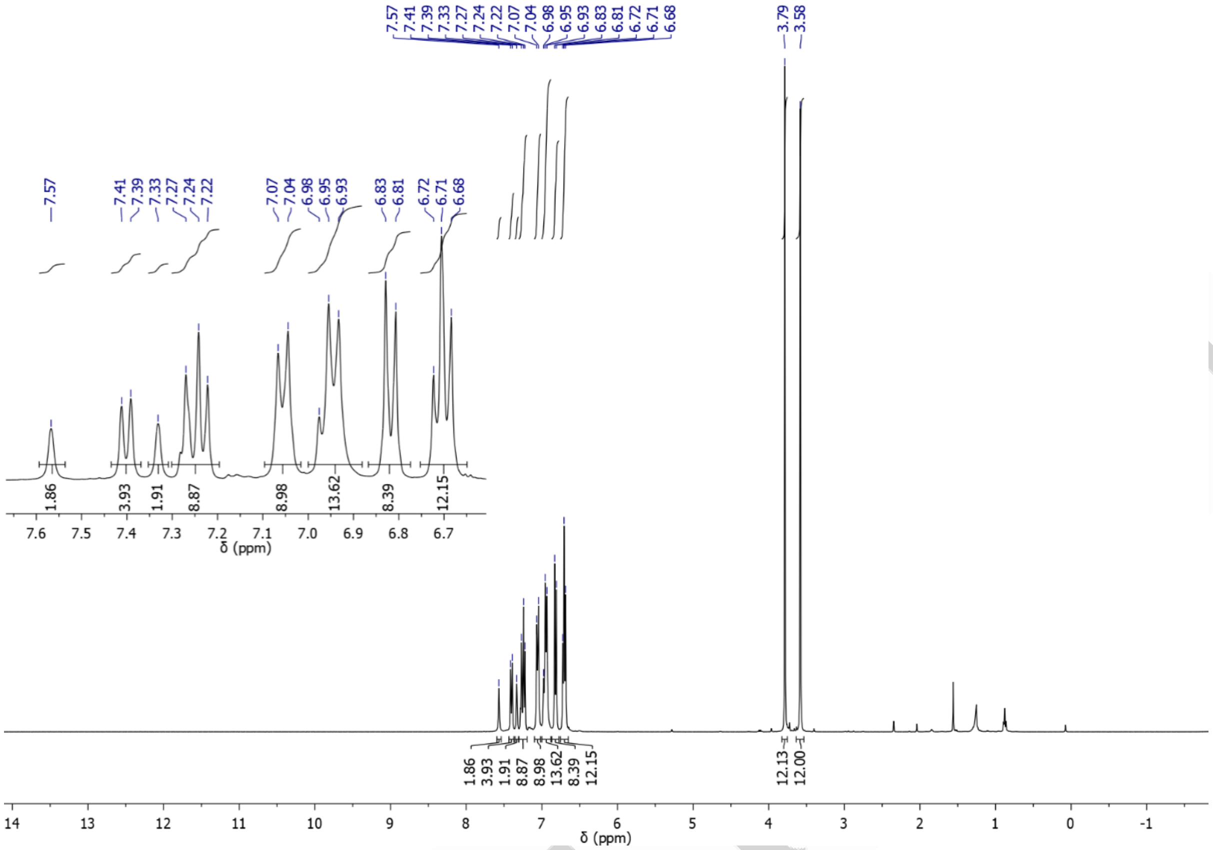

Figure S13. ^{1}H NMR (400 MHz, $CDCl_3$, 298 K) of compound **TDF 1.**

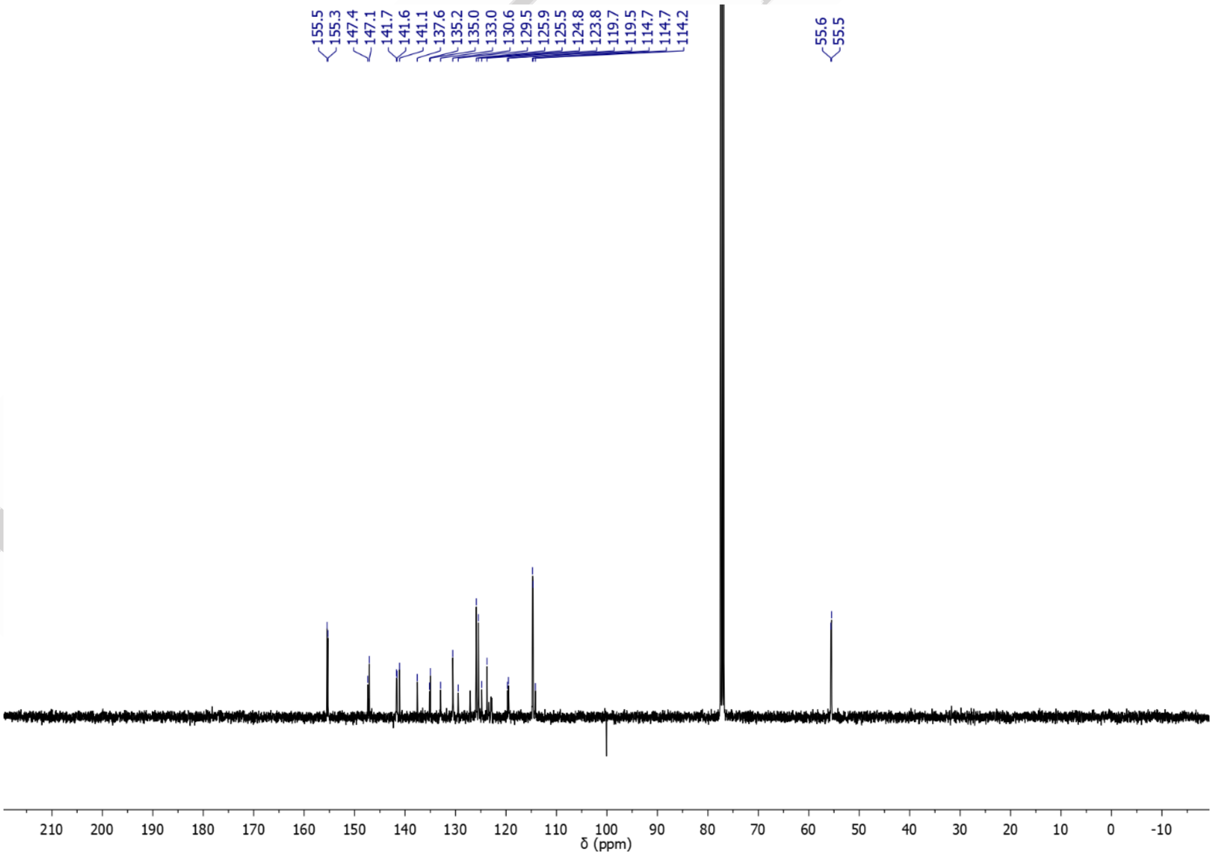

Figure S14. ^{13}C NMR (100 MHz, $CDCl_3$, 298 K) of compound **TDF 1.**

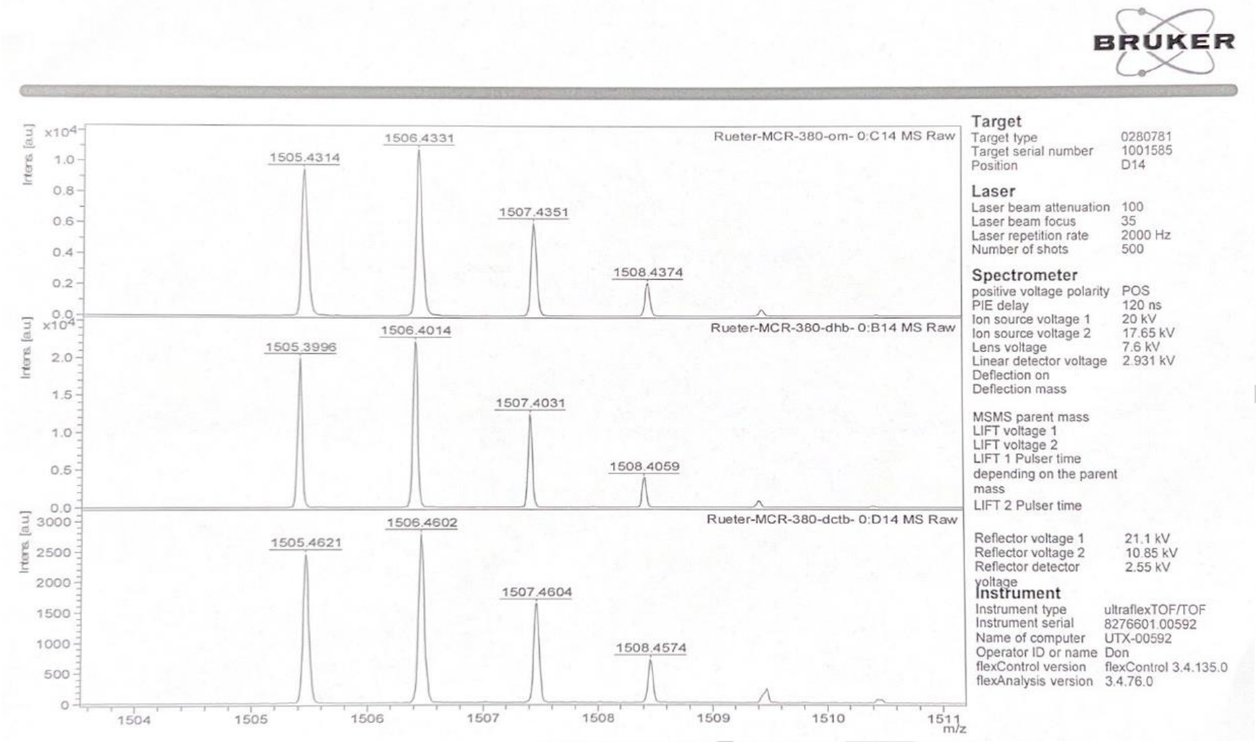

Figure S15. HR-MALDI-TOF mass spectrum of compound **TDF 1.**

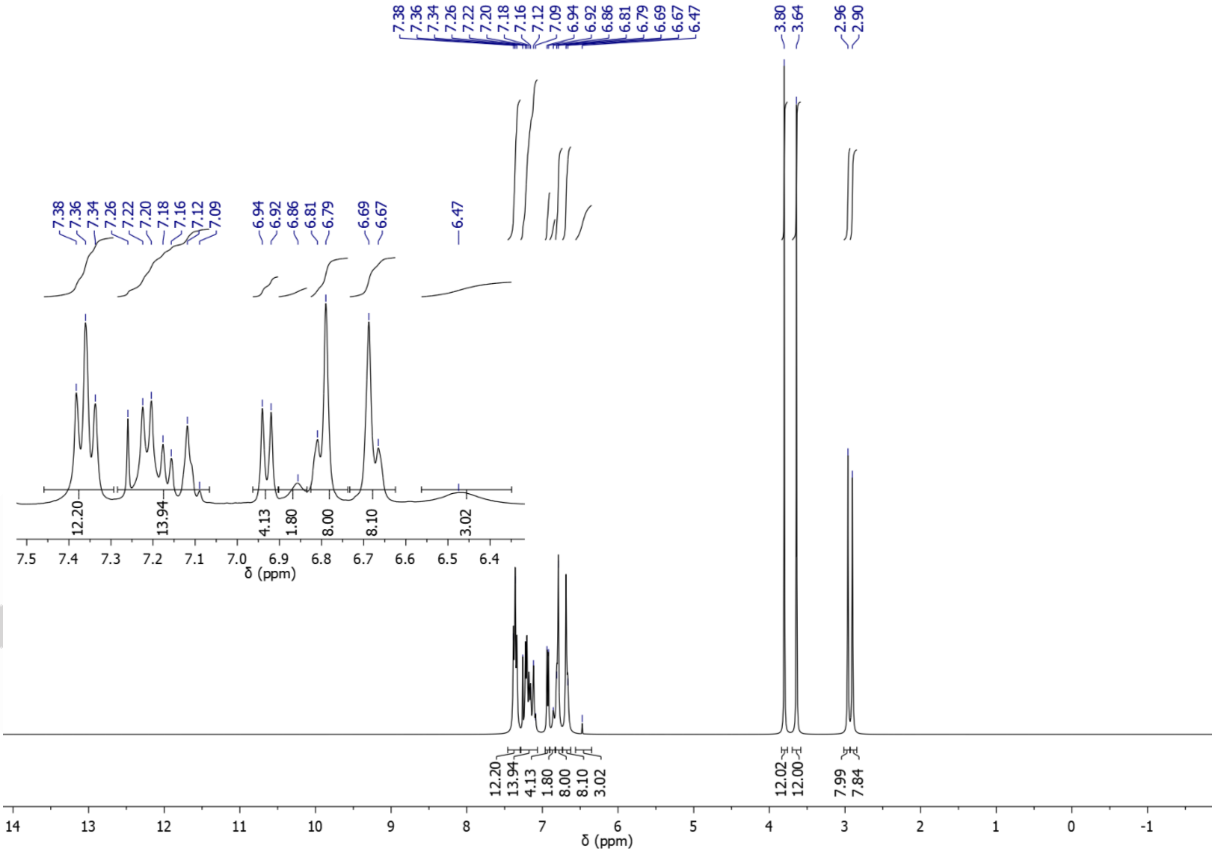

Figure S16. ^{1}H NMR (400 MHz, $CDCl_3$, 298 K) of compound **TDF 2.**

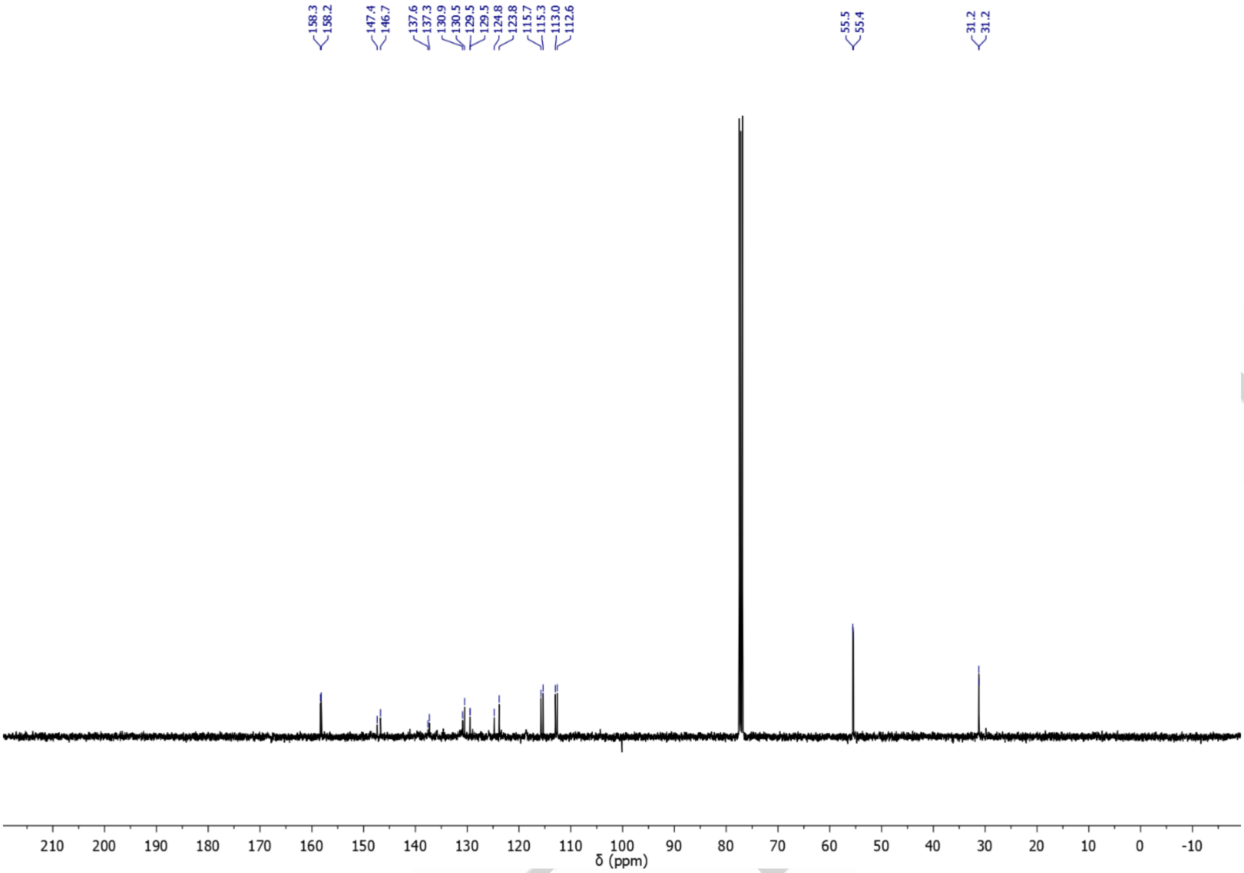

Figure S17. ^{13}C NMR (100 MHz, $CDCl_3$, 298 K) of compound **TDF 2.**

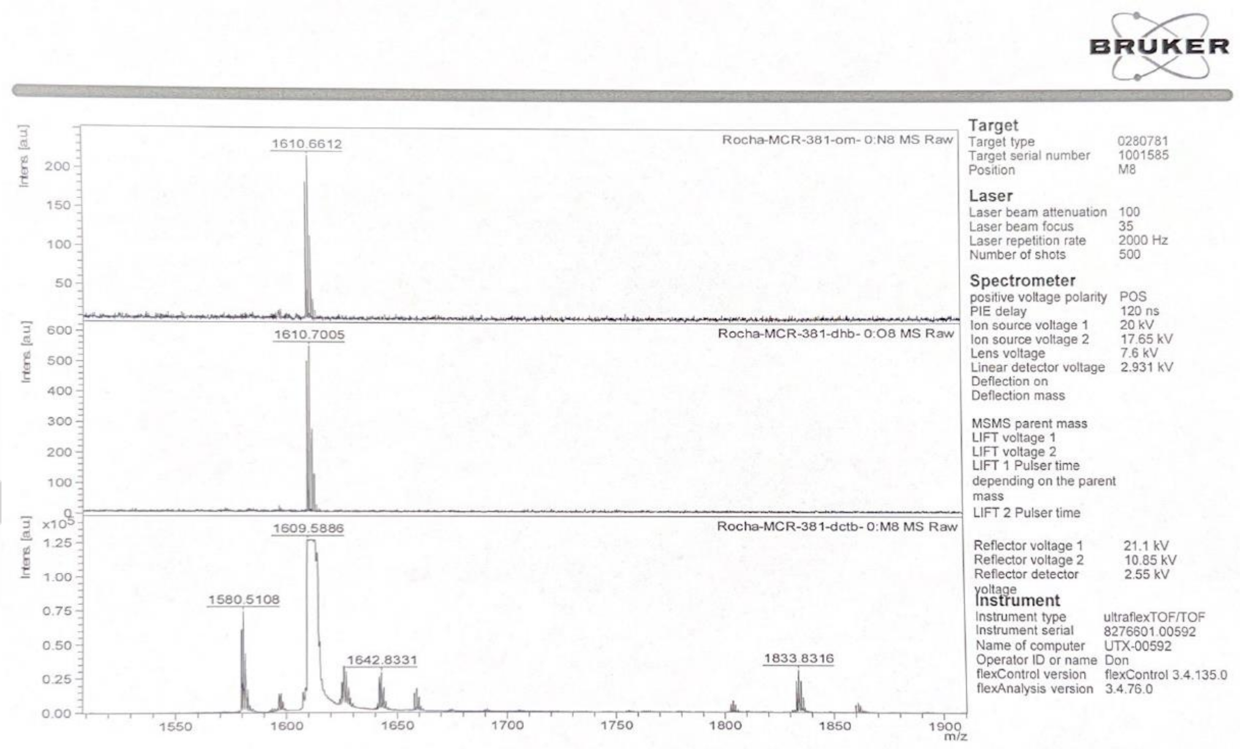

Figure S18. HR-MALDI-TOF mass spectrum of compound **TDF 2.**

3. Synthetic cost analysis for HTMs

Table S1. Materials quantities and cost for the synthesis of **5**.

**Chemicals for compound 5**	**CAS**	**Weight Reagent g**	**Weight Solvent g**	**Weight Workup g**	**Chemical´s Price $/g**	**Chemical Cost $**	**Target product $/g**
Compound 2	4181-05-9	0.362			7	2.534	
NaOH	1310-73-2	0.008			0.167	0.001336	
2,7-dibromofluorene	16433-88-8	0.642			5.36	3.44112	
Tetrabutylammonium bromide	1643-19-2	0.155			0.14	0.0217	21.866312
Toluene	108-88-3		17.338		0.055081324	0.955	
Dicloromethane	75-09-2			79,8	0.042105263	3.36	
anhydrous sodium sulfate				5	0.124	0.62	

Table S2. Materials quantities and cost for the synthesis of **TMF-1**.

**Chemicals for compound TMF-1**	**CAS**	**Weight Reagent g**	**Weight Solvent g**	**Weight Workup g**	**Chemical´s Price $/g**	**Chemical Cost $**	**Target product $/g**
Compound 5		0.1			10.933156	1.0933156	
4,4′-dimethoxydiphenylamine	101-70-2	0.123			76.7	9.4341	142.6687991
$Pd_2(dba)_3$	51364-51-3	0.00778175			23	0.17898025	
Xphos	564483-18-7	0.00810424			35.2	0.285269248	
Potassium tert-butoxide	865-47-4	0.076			12.6	0.9576	
Toluene	108-88-3		17.338		0.055081324	0.955	
Silica gel	112926-00-8			100	0.02552	2.552	

Table S3. Materials quantities and cost for the synthesis of 2,8-dibromo-10,11-dihydro-5H-dibenzo[b,f]azepine.

**Chemicals for 2,8-dibromo-10,11-dihydro-5H-dibenzo[b,f]azepine**	**CAS**	**Weight Reagent g**	**Weight Solvent g**	**Weight Workup g**	**Chemical´s Price $/g**	**Chemical Cost $**	**Target product $/g**
Iminodibenzyl	494-19-9	0.9763			2.12	2.069756	
DCM	75-09-2		19.899		0.042213177	0.84	
Silica gel	112926-00-8			2	0.02552	0.05104	
N-bromosuccinimid	128-08-5	1.78			0.416	0.74048	41.4239635
DCM	75-09-2		33.165		0.042213177	1.4	
DMF	68-12-2		14.25		0.107592105	1.5331875	
DCM	75-09-2			66.33	0.042213177	2.8	
n-hexane	110-54-3			198	0.140151515	27.75	
EtOAc	141-78-6			27	0.0625	1.6875	
Silica gel	112926-00-8			100	0.02552	2.552	

Table S4. Materials quantities and cost for the synthesis of compound **8**.

Chemicals for compound 8	**CAS**	**Weight Reagent g**	**Weight Solvent g**	**Weight Workup g**	**Chemical´s Price $/g**	**Chemical Cost $**	**Target product $/g**
Na	7440-23-5	3.2			1.1	3.52	
2,8-dibromo-10,11-dihydro-5H-dibenzo[b,f]azepine		1.1			41.4239635	45.56635985	
CuI	7681-65-4	1.18			0.992	1.17056	
MeOH	67-56-1		19.8		0.095959596	1.9	85.95529485
DMF	68-12-2		28.5		0.107592105	3.066375	
EtOAc	141-78-6		54		0.0625	3.375	
Na_2SO_4	7757-82-6			5	0.071	0.355	
n-hexane	110-54-3		158.4		0.140151515	22.2	

EtOAc	141-78-6		36		0.0625	2.25	
Silica gel	112926-00-8			100	0.02552	2.552	

Table S5. Materials quantities and cost for the synthesis of **TMF-2**.

Chemicals for TMF 2	CAS	Weight Reagent g	Weight Solvent g	Weight Workup g	Chemical´s Price $/g	Chemical Cost $	Target product $/g
Compound 5		0.1			10.933156	1.0933156	
Compound 8		0.105			85.95529485	9.025305959	312.2912643
$Pd_2(dba)_3$	51364-51-3	0.00778175			23	0.17898025	
Xphos	564483-18-7	0.00810424			35.2	0.285269248	
Potassium tert-butoxide	865-47-4	0.076			12.6	0.9576	
Toluene	108-88-3		17.338		0.055081324	0.955	
DCM	75-09-2		278.586		0.042213177	11.76	
n-hexane	110-54-3		59.4		0.140151515	8.325	
Silica gel	112926-00-8			100	0.02552	2.552	

Table S6. Materials quantities and cost for the synthesis of Compound **6**.

Chemicals for compound 6	CAS	Weight Reagent g	Weight Solvent g	Weight Workup g	Chemical´s Price $/g	Chemical Cost $	Target product $/g
Compound 3	53566-95-3	0.8			37	29.6	
NaOH	1310-73-2	0.008			0.167	0.001336	44.10496
2,7-dibromofluorene	16433-88-8	2.6			5.36	13.936	
Tetrabutylammonium bromide	1643-19-2	0.308			0.14	0.04312	
Toluene	108-88-3		17.338		0.055081324	0.955	
DCM	75-09-2			79.8	0.042105263	3.36	
anhydrous sodium sulfate				5	0.124	0.62	

Table S7. Materials quantities and cost for the synthesis of Compound **TDF-1**.

Chemicals for TDF-1	CAS	Weight Reagent g	Weight Solvent g	Weight Workup g	Chemical´s Price $/g	Chemical Cost $	Target product $/g
compound 6		0.1			48.515456	4.8515456	
4,4′-dimethoxydiphenylamine	101-70-2	0.122			76.7	9.3574	
$Pd_2(dba)_3$	51364-51-3	0.0100705			23	0.2316215	
Xphos	564483-18-7	0.01048784			35.2	0.369171968	
Potassium tert-butoxide	865-47-4	0.1			12.6	1.26	423.6187719
Toluene	108-88-3		17.338		0.055081324	0.955	
DCM	75-09-2		318.384		0.042213177	13.44	
n-hexane	110-54-3		39.6		0.140151515	5.55	
Silica gel	112926-00-8			100	0.02552	2.552	

Table S8. Materials quantities and cost for the synthesis of Compound **TDF-2**.

Chemicals for TDF 2	CAS	Weight Reagent g	Weight Solvent g	Weight Workup g	Chemical´s Price $/g	Chemical Cost $	Target product $/g
Compound 6		0.1			48.515456	4.8515456	
Compound 8		0.122			85.95529485	10.48654597	
$Pd_2(dba)_3$	51364-51-3	0.0100705			23	0.2316215	413.2087941
Xphos	564483-18-7	0.01048784			35.2	0.369171968	
Potassium tert-butoxide	865-47-4	0.1			12.6	1.26	

WILEY-VCH

Toluene	108-88-3	17.338		0.055081324	0.955
DCM	75-09-2	318.384		0.042213177	13.44
n-hexane	110-54-3	39.6		0.140151515	5.55
Silica gel	112926-00-8		100	0.02552	2.552

4. Kelvin probe measurement

The sample was prepared on ITO substrate that was treated by oxygen-plasma for 3 min before use. The processing of each layer was the same as the device fabrication. WF was measured by a SPS040 system. Before the measurements, a highly oriented pyrolytic graphite (HPOG) with a fresh surface was used to calibrate the probe before every measurement, and all measurements were carried on in air at room temperature and humidity around 35%.

The work function of the tip (WF_{Tip}) was calculated as follows:

$$WF_{Tip}=4500 - CPD_{HPOG}$$

$$WF_{Tip}= 4500-(48) = 4452meV$$

For the measurements of the work function it could be taken the ITO as an example:

$$WF_{ITO}= 4452+CPD_{ITO}$$

$$WF_{ITO}= 4452+298 = 4750 meV$$

The CPD were taken from the mean value of the measurements from each sample as shown in the following table.

Table S9. Statistics on the values for the contact potential difference measurement.

Sample	N total	Mean	Standard Deviation	Sum	Minimum	Median	Maximum
**ITO**	410	298.575	2.188	122415.9	292.2	298.7	304.0
**TMF-1/ITO**	637	95.867	5.895	62026.0	73.6	95.0	110.7
**TMF-2/ITO**	733	-11.717	1.999	-6854.5	-17.7	-11.6	-4.6
**TDF-1/ITO**	585	12.158	2.235	8510.3	1.5	12.2	18.4
**TDF-2/ITO**	606	61.799	2.003	44989.5	55.9	61.8	68.6

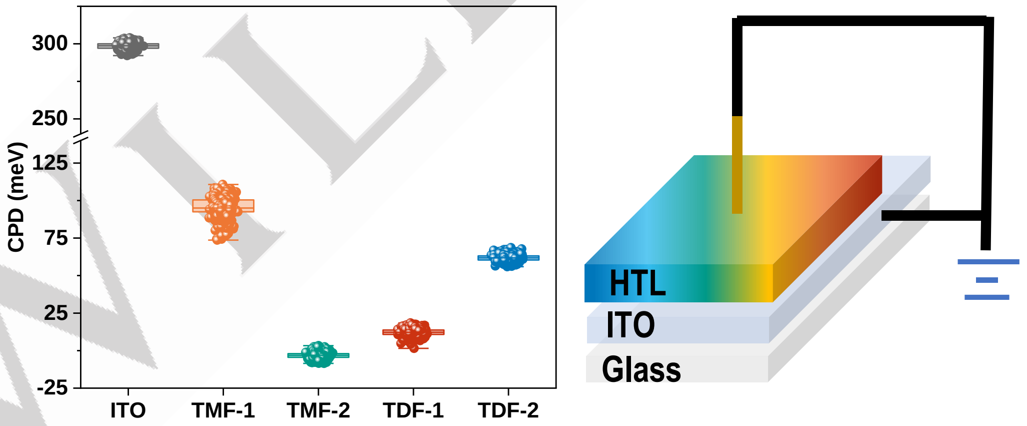

Figure S19. Contact potential difference measurement on ITO substrates with a layer of **TMF** and **TDF** series.

5. UVC degradation measurements

WILEY-VCH

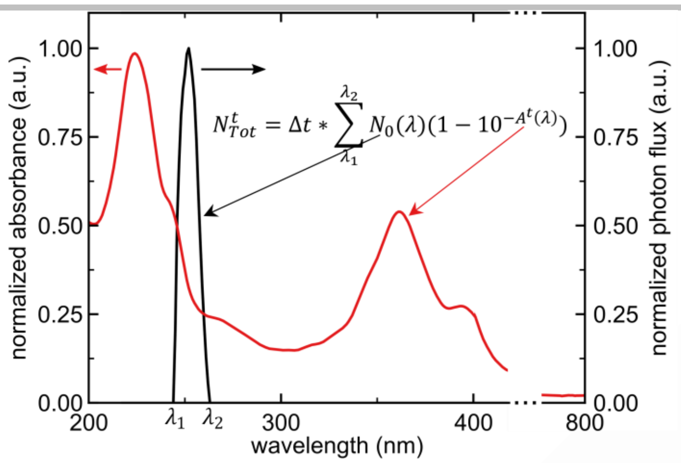

6.

Figure S20. Schematic showing how the absorbed photon flux N_{Tot}^{t} for a time t can calculated from the photon flux $N_0(\lambda)$ at UVC wavelengths ($\lambda_1 = 244$ nm, $\lambda_2 = 263$ nm) and the absorbance $A^t(\lambda)$ at time t[3]. The black curve refers to the emission spectrum of the UVC lamp and the red curve is an exemplary UV-Vis spectrum of a thin film on a quartz substrate.

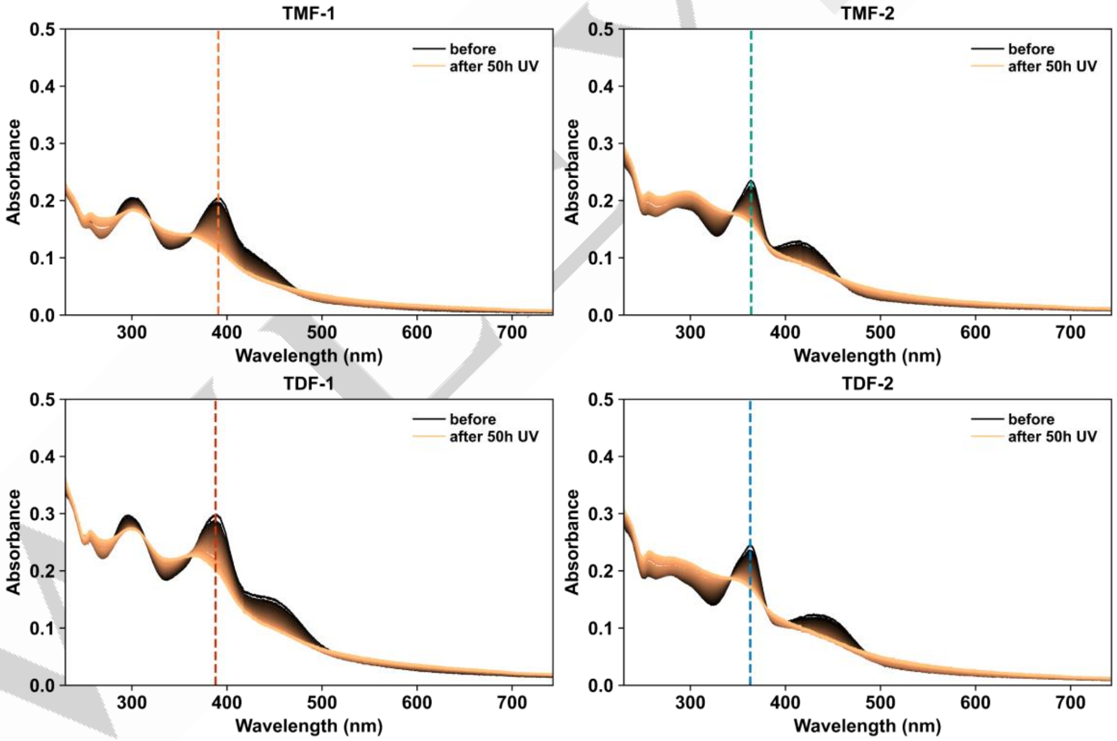

Fig. S21. UV-Vis spectra of **TMF-1-2** and **TDF-1-2** in thin films before and during 50 h of UVC exposure on quartz substrates. The dashed lines highlight the peaks whose normalized absorbance was tracked over number of absorbed UVC photons in Figure 2e.

7. Contact angle measurements

WILEY-VCH

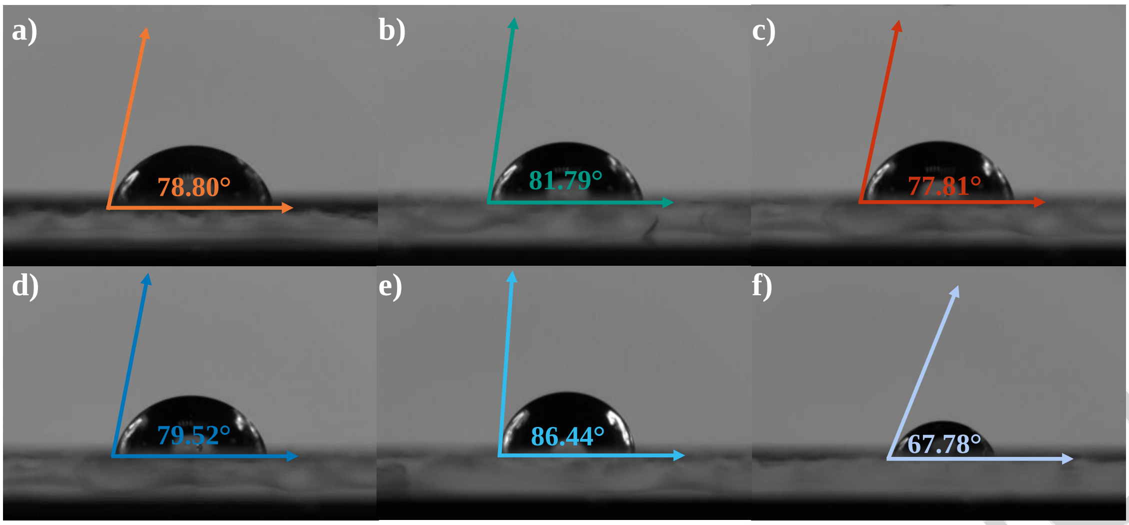

Figure S22. Snapshots of water contact angles (CAs) on various substrates: (a) TMF-1/ITO, (b) TMF-2/ITO, (c) TDF-1/ITO, (d) TDF-2/ITO, (e) PTAA/ITO, and (f) ITO. Each CA was measured using a 12 μL water droplet.

8. Theoretical Calculations

WILEY - VCH

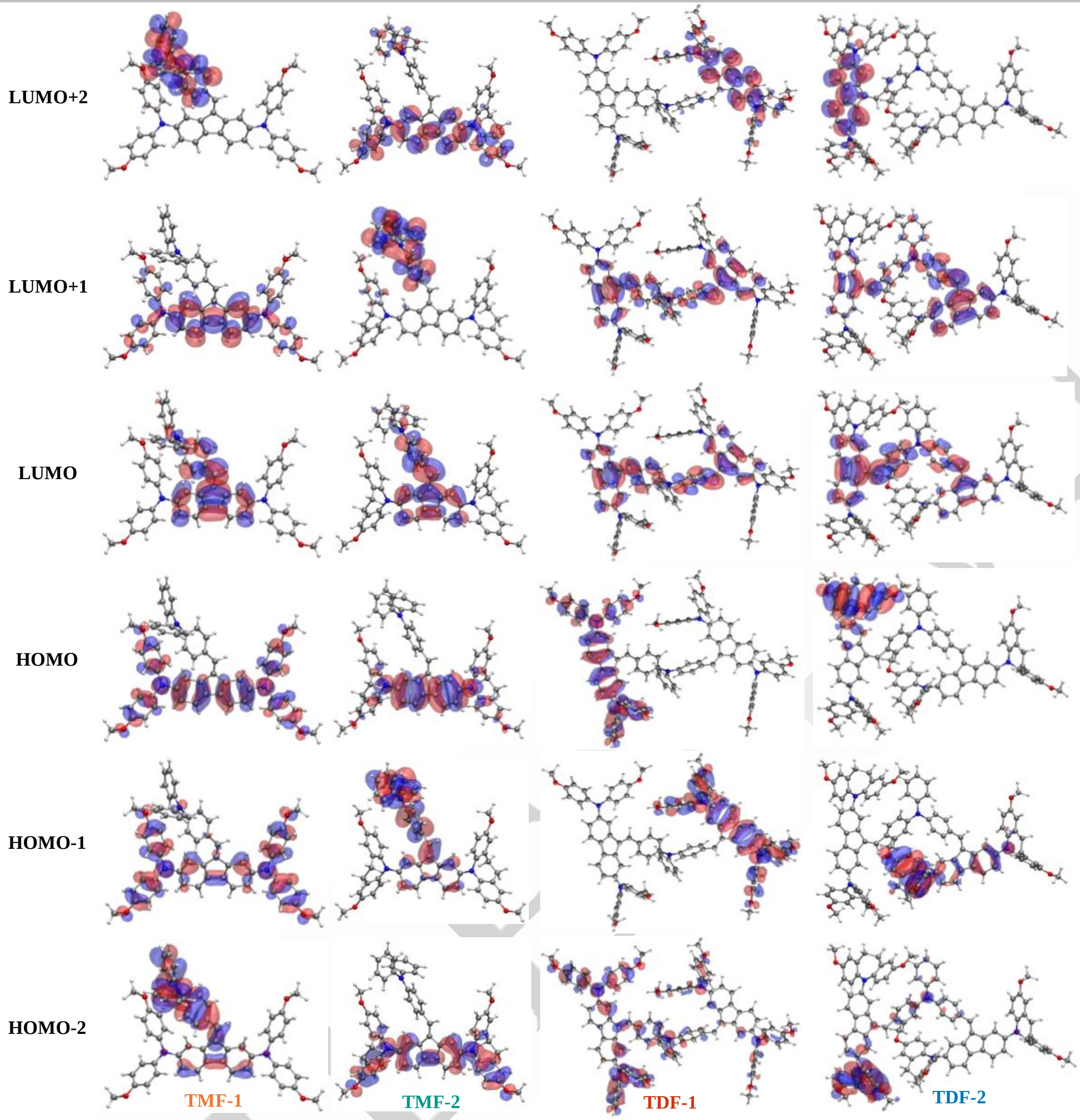

Figure S23. Electron density maps of the frontier molecular orbitals HOMOs and LUMOs of **TMF-1-2** and **TDF-1-2**.

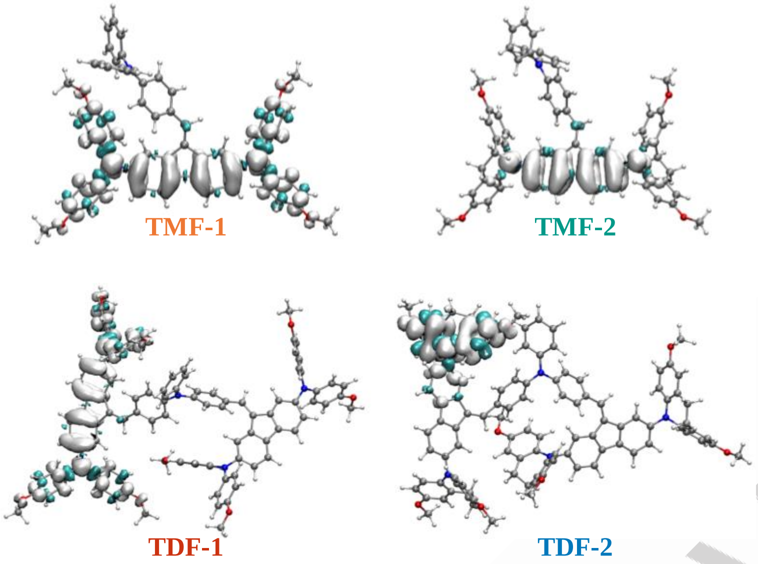

Figure S24. Unpaired-electron spin densities (isovalue = 0.001) calculated for the most stable spin configuration of the cation species of **TMF-1-2** and **TDF-1-2** at the CAM-B3LYP/6-31G(d,p) level in THF.

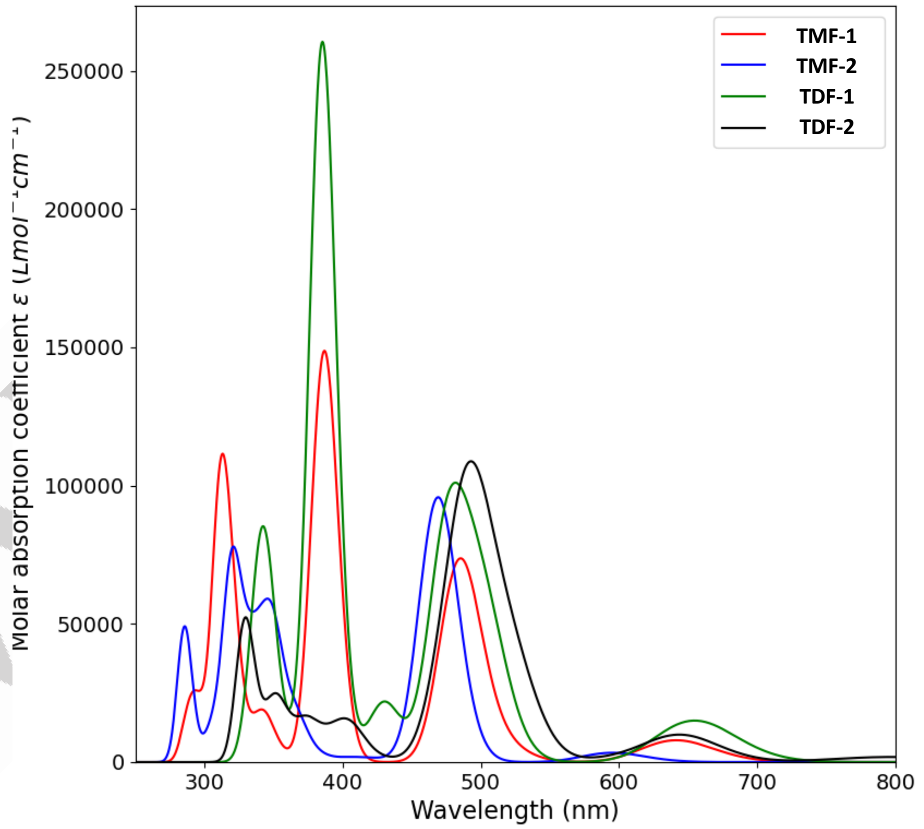

Figure S25. Simulated UV spectra for **TMF-1-2** and **TDF-1-2** from TD-DFT calculations (B3LYP/6-31G(d) level of calculation) in THF solution.

9. SEM measurements

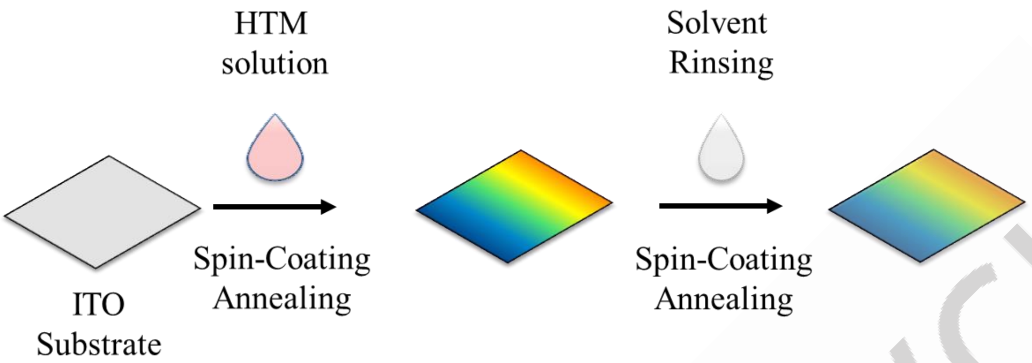

Figure S26. Diagram for HTMs deposition on ITO substrates and its rinsing process simulating the perovskite deposition with DMF/DMSO mixed solution (4:1/v:v).

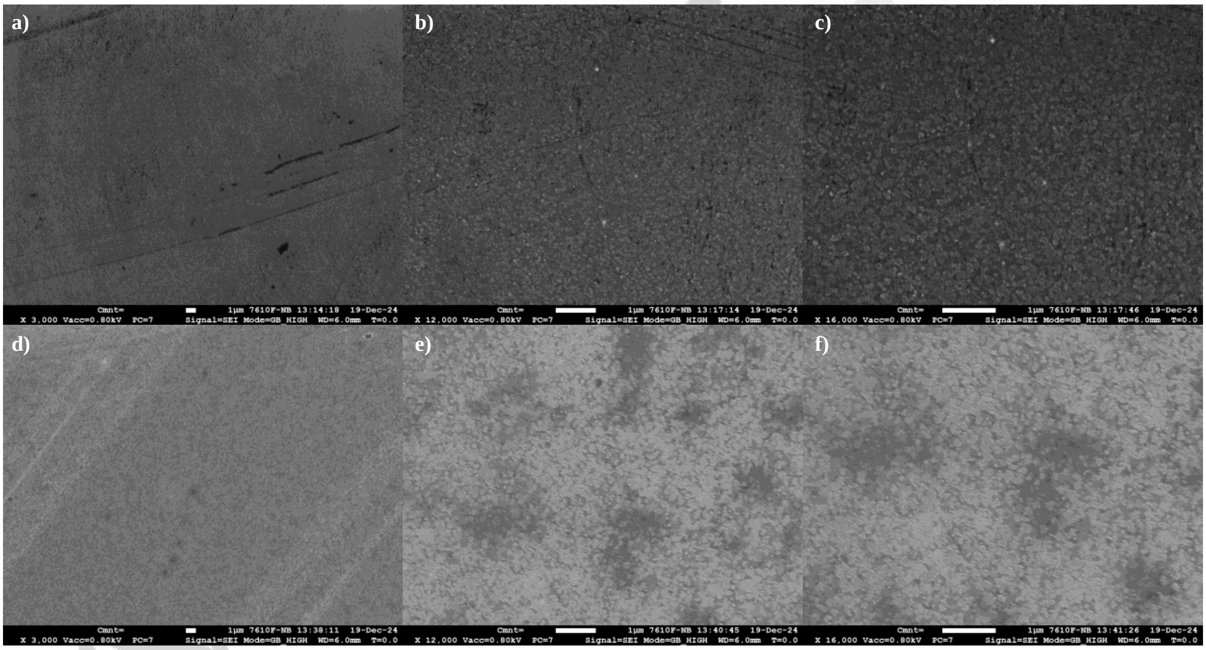

Figure S27. Top-view SEM images of TMF-1 film over an ITO/glass substrate before rinsing with a) 3000, b) 12000 and c) 16000, and after rinsing with d) 3000, f) 12000 and g) 16000 times magnification.

WILEY-VCH

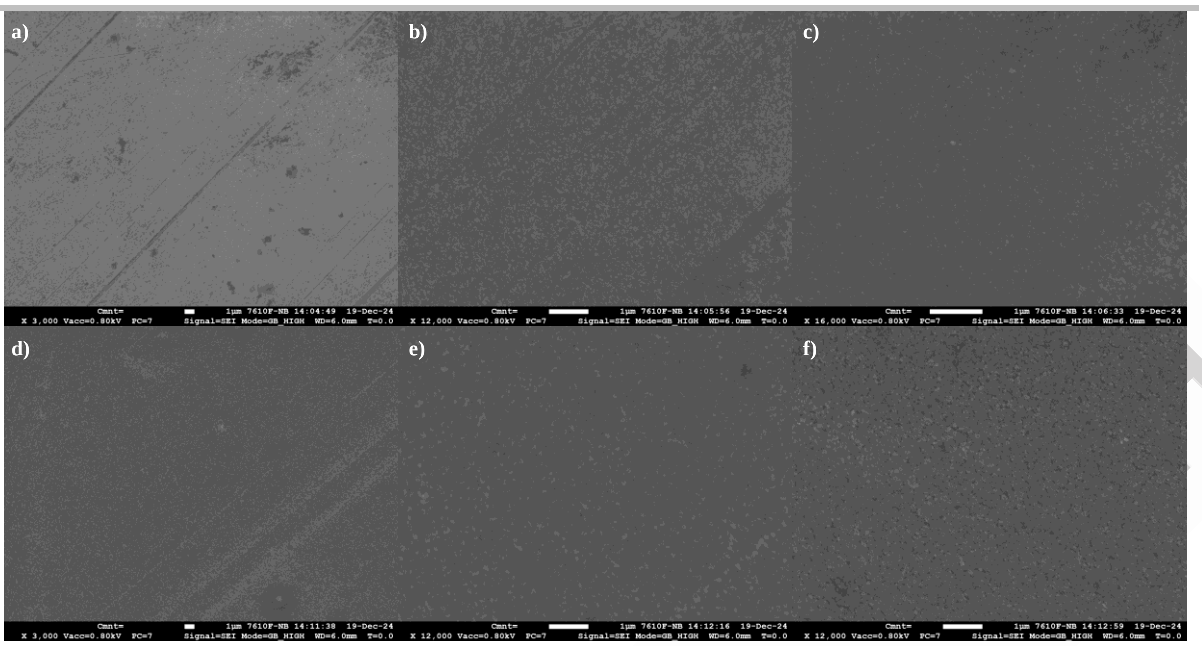

Figure S28. Top-view SEM images of **TMF-2** film over an ITO/glass substrate before rinsing with a) 3000, b) 12000 and c) 16000, and after rinsing with d) 3000, f) 12000 and g) 16000 times magnification.

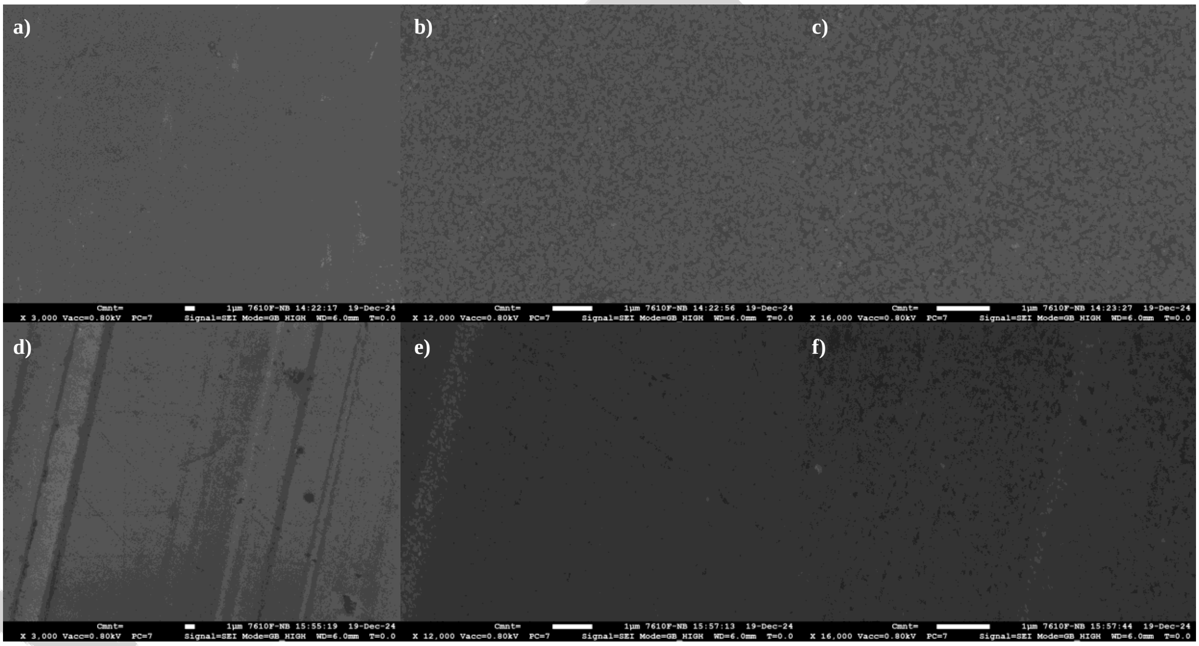

Figure S29. Top-view SEM images of **TDF-1** film over an ITO/glass substrate before rinsing with a) 3000, b) 12000 and c) 16000, and after rinsing with d) 3000, f) 12000 and g) 16000 times magnification.

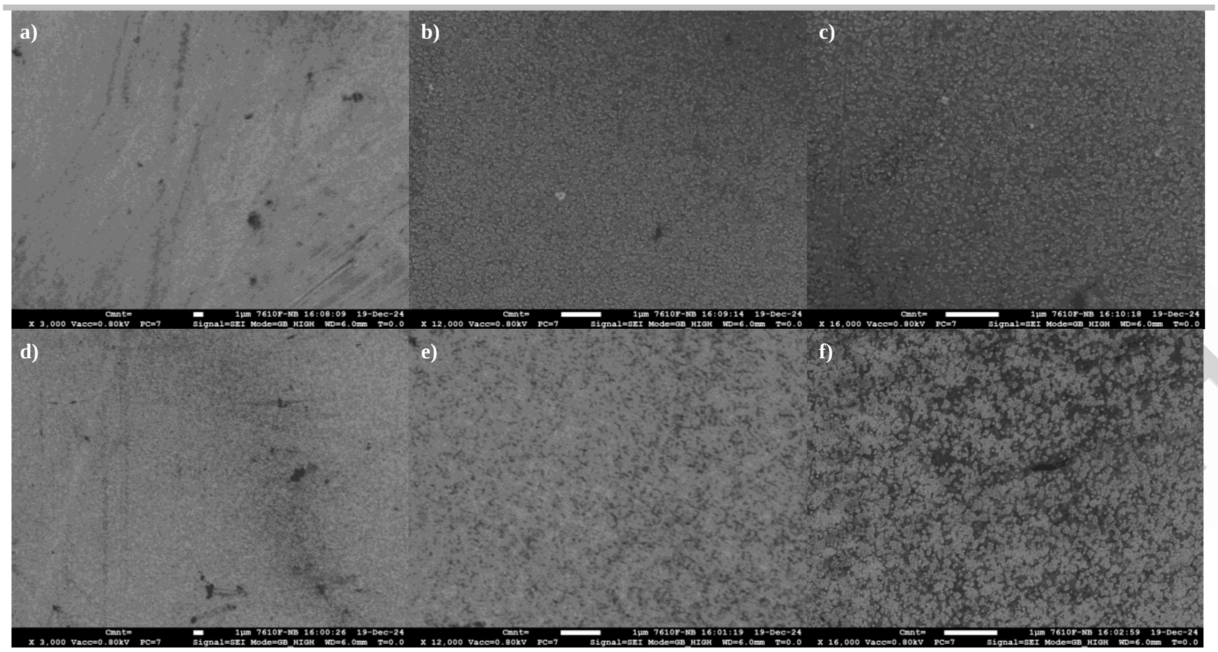

Figure S30. Top-view SEM images of **TDF-2** film over an ITO/glass substrate before rinsing with a) 3000, b) 12000 and c) 16000, and after rinsing with d) 3000, f) 12000 and g) 16000 times magnification.

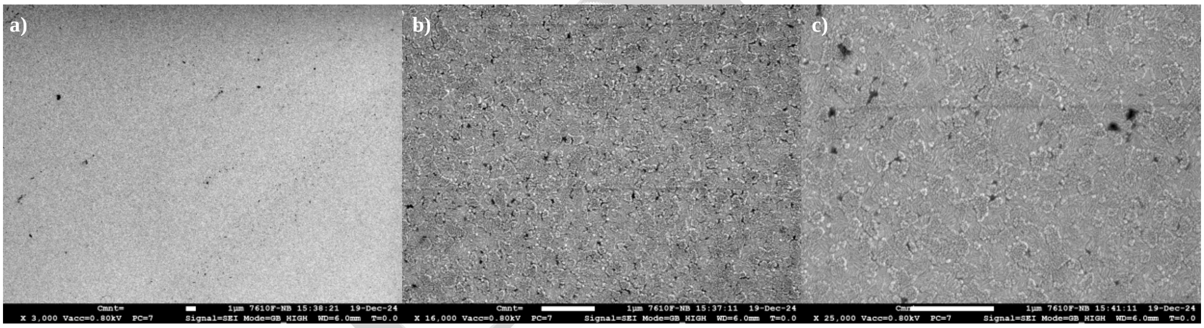

Figure S31. Top-view SEM images of an ITO/glass substrate with a) 3000, b) 16000 and c) 25000 times magnification

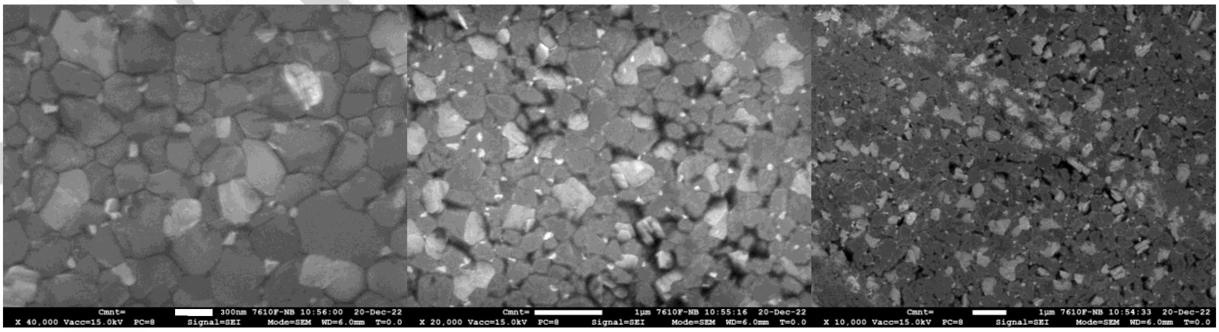

Figure S32. Top-view SEM images of perovskite film over a **TMF-1** layer with a) 10000-, b) 20000- and c) 40000-times magnification.

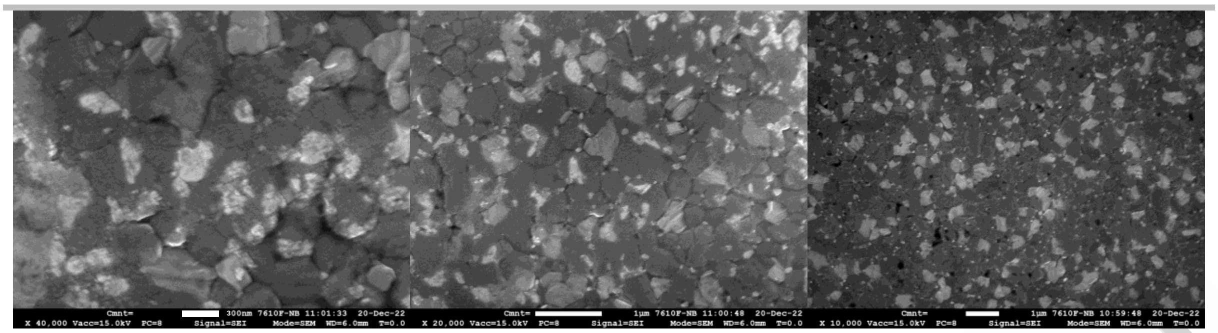

Figure S33. Top-view SEM images of perovskite film over a **TMF-2** layer with a) 10000-, b) 20000- and c) 40000-times magnification.

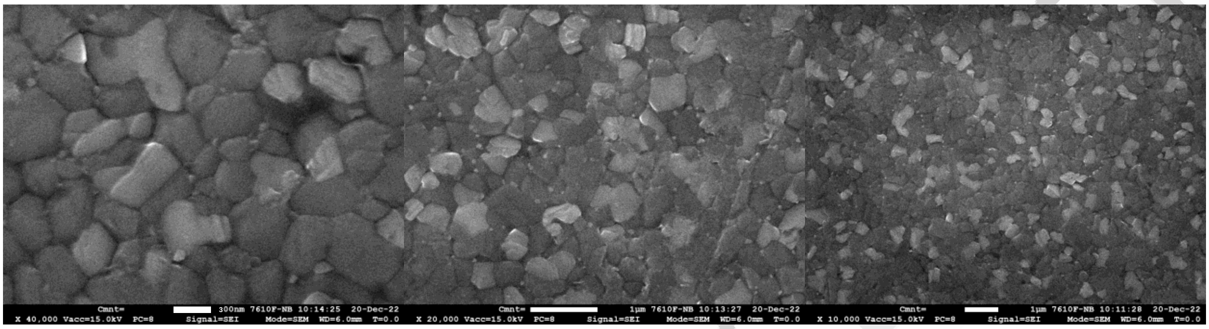

Figure S34. Top-view SEM images of perovskite film over a **TDF-1** layer with a) 10000-, b) 20000- and c) 40000-times magnification.

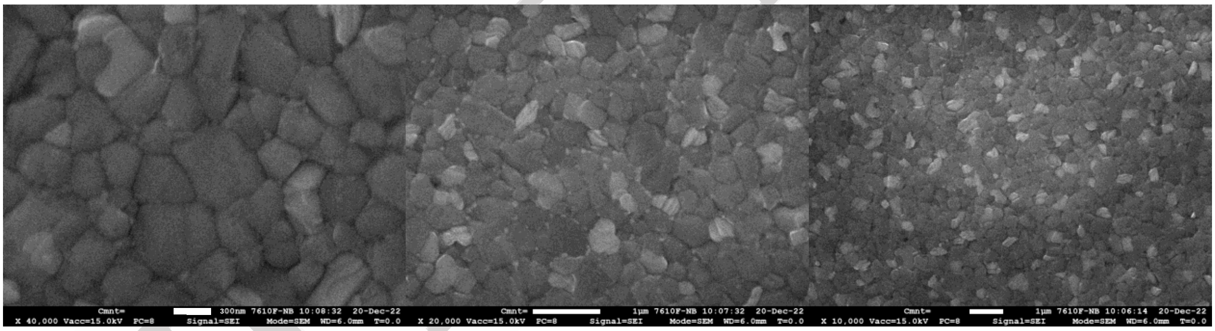

Figure S35. Top-view SEM images of perovskite film over a **TDF-2** layer with a) 10000-, b) 20000- and c) 40000-times magnification.

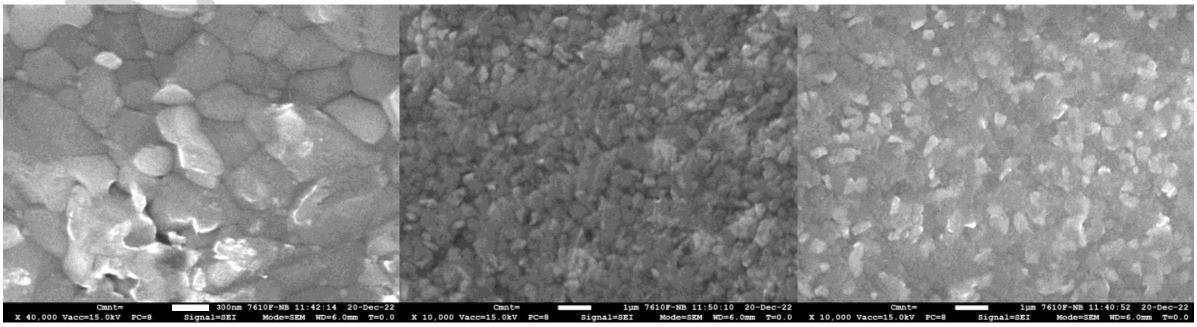

Figure S36. Top-view SEM images of perovskite film over a **PTAA** layer with a) 10000-, b) 20000- and c) 40000-times magnification.

Table S10. Lifetime and weight fractions fitted from TRPL decay curves.

Compound	τ_1 (ns)	A_1	τ_2 (ns)	A_2	τ_{avg} (ns)[a]
**PVK**	8.53179 ± 0.2001	58.39239 ± 1.16956	378.9472 ± 1.12608	58.20819 ± 0.12034	370.7658
**TMF-1/PVK**	6.50351 ± 0.10992	61.24575 ± 0.8551	152.36555 ± 0.55337	51.40511 ± 0.13127	145.3067
**TMF-2/PVK**	7.20008 ± 0.08995	52.5372 ± 0.53387	221.36106 ± 0.38682	61.82808 ± 0.07315	215.6011
**TDF-1/PVK**	8.63113 ± 0.31574	34.8573± 1.11538	219.44103 ± 0.74338	71.9196 ± 0.14492	215.4974
**TDF-2/PVK**	6.66182 ± 0.11517	53.69803± 0.71786	115.19755 ± 0.3547	60.04435 ± 0.14789	109.8603
**PTAA/PVK**	5.37203 ± 0.03009	103.79179 ± 0.4504	68.53654 ± 0.34822	22.63827 ± 0.10985	51.8382

a) The τ_{avg} is calculated from the following equation: $\tau_{avg} = (A_1\tau_1^2 + A_2\tau_2^2)/(A_1\tau_1 + A_2\tau_2)$, representing the average lifetime of carriers.

10. Hole mobility measurements

To investigate the charge-carrier mobilities of synthesized materials, hole-only devices were fabricated with device structure of Indium tin oxide (ITO) glasses/(poly(3,4-ethylenedioxythiophene):poly(styrenesulfonate), PEDOT:PSS)/ HTM/MoO_3/Ag. Hole mobilities of were measured via space charge limitation of current (SCLC) under dark conditions according to previous literature.[3,4] The mobility was obtained by fitting the SCLC curve.[5] The applied bias was varied from 0 to 6 V.

11. Device Fabrication

ITO glass substrates(2.5cm×2.5cm) were cleaned by sonication with acetone and isopropanol for 30 min, sequentially, the substrates were dried with N_2 and cleaned by UV ozone for 15 min. The cleaned glass substrates were transferred into the glove box for the following film preparation. 70 µL of **TM-01**, **TM-02**, **TM-03**, **TM-04** and PTAA solutions in chlorobenzene (CB, 3 mg/mL) were spin-coated on the ITO at 5000 rpm for 30 s as the HTLs. The fabricated HTLs were then thermally annealed on the hotplate at 100 °C for 10 min. The perovskite precursor solution was prepared by dissolving FAI, PbI_2, CsI, MACl in a molar ratio of 1.2, 1.5, 0.25 and 0.07, respectively, in a 1 mL DMF/DMSO mixed solution (V/V= 4/1). 70 µL of the perovskite solution was spin-coated on the HTL at 1000 rpm for 5 s and 5000 rpm for 30 s, respectively. Note that 300 µL of anti-solvent (CB) was quickly dropped at the last 5 s of the

WILEY-VCH

film fabrication, followed by a two-step thermal annealing at 100 °C and 150 °C for 10 min each. Afterward, 80 µL of the phenethylammonium iodide (PEAI) solution in isopropanol (1 mg/mL) was spin-coated on the perovskite layer at 5000 rpm for 30 s, followed by thermal annealing at 100 °C for 10 mins. Subsequently, 50 µL of $PC_{61}BM$ solution (20 mg/mL in CB) was spin-coated on the PEAI layer at 1000 rpm for 60 s, followed by thermal annealing at 80 °C for 10 mins, then 60 µL of BCP solution (0.5 mg/mL in IPA) was spin-coated on $PC_{61}BM$ layer at 5000 rpm for the 30 s, followed by thermal annealing at 80 °C for 5 mins. Finally, the 100 nm Ag electrode was evaporated under a high vacuum. The active area was controlled using a shadow mask with an area of 0.06 cm^2.

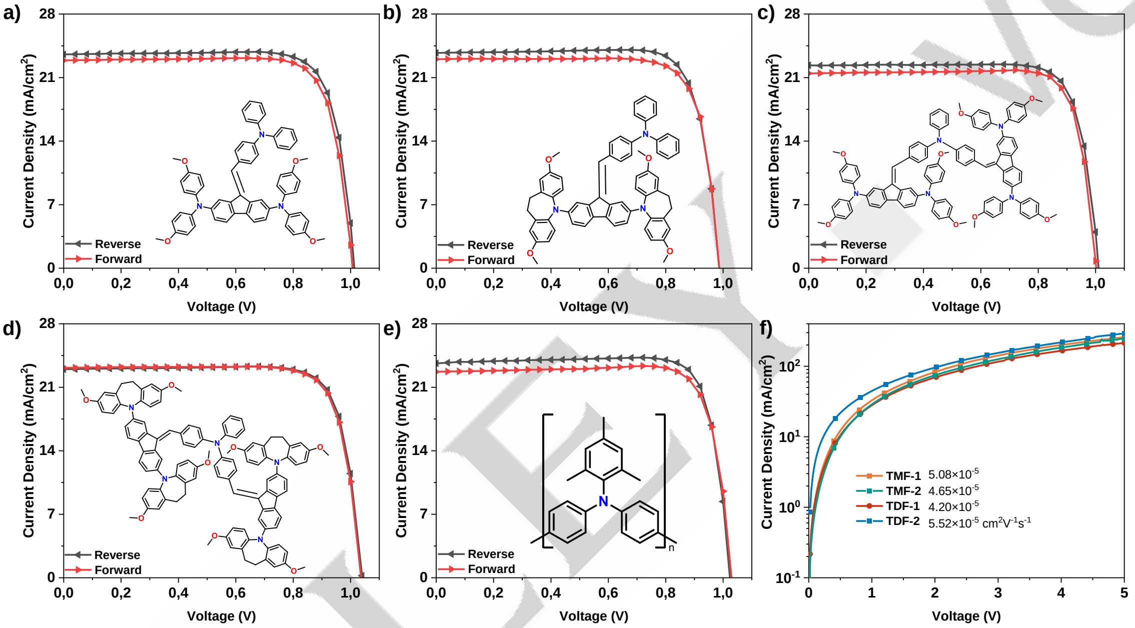

Figure S37. *J-V* curves of the a) **TMF-1**, b) **TMF-2**, c) **TDF-1**, d) **TDF-2** and e) **PTAA** champion devices measured using both forward and reverse scan mode under the simulation of AM 1.5G, 100 mW cm $^{-2}$ and f) *J−V* plots of hole-only devices based on **TMF-1-2**, **TDF-1-2** and **PTAA**.

WILEY-VCH

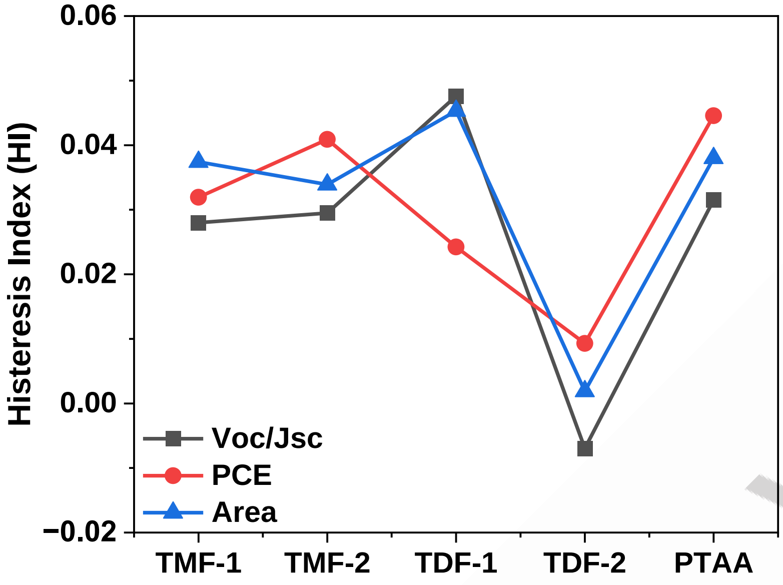

Fig. S38. Hysteresis index calculated for the devices based on **TMF-1-2** and **TDF-1-2** series and PTAA.

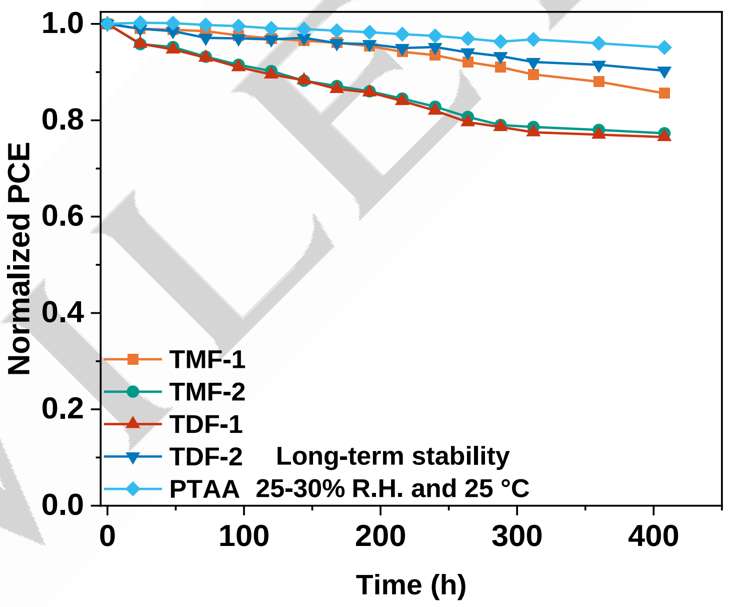

Fig. S39. Stability test for unencapsulated PSC devices in ambient condition with a relative humidity of 25–30% for **TMF-1-2** and **TDF-1-2** series and PTAA in combination with the $Cs_{0.17}FA_{0.83}PbI_3$ perovskite in planar PSC devices.

12. **References**

[1] C. A. Echeverry, A. Insuasty, M. Á. Herranz, A. Ortíz, R. Cotta, V. Dhas, L. Echegoyen, B. Insuasty, N. Martín, *Dyes and Pigments* **2014**, *107*, 9–14.
[2] R. Pashazadeh, P. Pander, A. Bucinskas, P. J. Skabara, F. B. Dias, J. V. Grazulevicius, *Chemical Communications* **2018**, *54*, 13857–13860.
[3] J. A. Röhr, D. Moia, S. A. Haque, T. Kirchartz, J. Nelson, *Journal of Physics Condensed Matter* **2018**, *30*, DOI 10.1088/1361-648X/aaabad.

[4] V. M. Le Corre, E. A. Duijnstee, O. El Tambouli, J. M. Ball, H. J. Snaith, J. Lim, L. J. A. Koster, *ACS Energy Lett* **2021**, *6*, 1087–1094.
[5] J. Wang, H. Zhang, B. Wu, Z. Wang, Z. Sun, S. Xue, Y. Wu, A. Hagfeldt, M. Liang, *Angewandte Chemie - International Edition* **2019**, *58*, 15721–15725.

WILEY-VCH
